# BET inhibition reforms the immune microenvironment and alleviates T cell dysfunction in chronic lymphocytic leukemia

**DOI:** 10.1172/jci.insight.177054

**Published:** 2024-05-22

**Authors:** Audrey L. Smith, Sydney A. Skupa, Alexandria P. Eiken, Timothy E. Reznicek, Elizabeth Schmitz, Nolan Williams, Dalia Y. Moore, Christopher R. D’Angelo, Avyakta Kallam, Matthew A. Lunning, R. Gregory Bociek, Julie M. Vose, Eslam Mohamed, Anna R. Mahr, Paul W. Denton, Ben Powell, Gideon Bollag, M. Jordan Rowley, Dalia El-Gamal

**Affiliations:** 1 Eppley Institute for Research in Cancer and Allied Diseases,; 2 Department of Genetics, Cell Biology and Anatomy,; 3 Division of Hematology and Oncology, Department of Internal Medicine, and; 4 Fred & Pamela Buffett Cancer Center (FPBCC), University of Nebraska Medical Center (UNMC), Omaha, Nebraska, USA.; 5 College of Medicine and College of Graduate Studies, California Northstate University, Elk Grove, California, USA.; 6 Department of Biology, University of Nebraska at Omaha, Omaha, Nebraska, USA.; 7 Plexxikon Inc., South San Francisco, California, USA.; 8 Opna Bio LLC, South San Francisco, California, USA.

**Keywords:** Immunology, Oncology, Drug therapy, Leukemias, T cells

## Abstract

Redundant tumor microenvironment (TME) immunosuppressive mechanisms and epigenetic maintenance of terminal T cell exhaustion greatly hinder functional antitumor immune responses in chronic lymphocytic leukemia (CLL). Bromodomain and extraterminal (BET) proteins regulate key pathways contributing to CLL pathogenesis and TME interactions, including T cell function and differentiation. Herein, we report that blocking BET protein function alleviates immunosuppressive networks in the CLL TME and repairs inherent CLL T cell defects. The pan-BET inhibitor OPN-51107 reduced exhaustion-associated cell signatures resulting in improved T cell proliferation and effector function in the Eμ-TCL1 splenic TME. Following BET inhibition (BET-i), TME T cells coexpressed significantly fewer inhibitory receptors (IRs) (e.g., PD-1, CD160, CD244, LAG3, VISTA). Complementary results were witnessed in primary CLL cultures, wherein OPN-51107 exerted proinflammatory effects on T cells, regardless of leukemic cell burden. BET-i additionally promotes a progenitor T cell phenotype through reduced expression of transcription factors that maintain terminal differentiation and increased expression of TCF-1, at least in part through altered chromatin accessibility. Moreover, direct T cell effects of BET-i were unmatched by common targeted therapies in CLL. This study demonstrates the immunomodulatory action of BET-i on CLL T cells and supports the inclusion of BET inhibitors in the management of CLL to alleviate terminal T cell dysfunction and potentially enhance tumoricidal T cell activity.

## Abstract 2

The BET inhibitor OPN-51107 alleviates immunosuppressive networks in the chronic lymphocytic leukemia tumor microenvironment and directly improves exhausted T cell progenitor potential and function.

## Graphical abstract

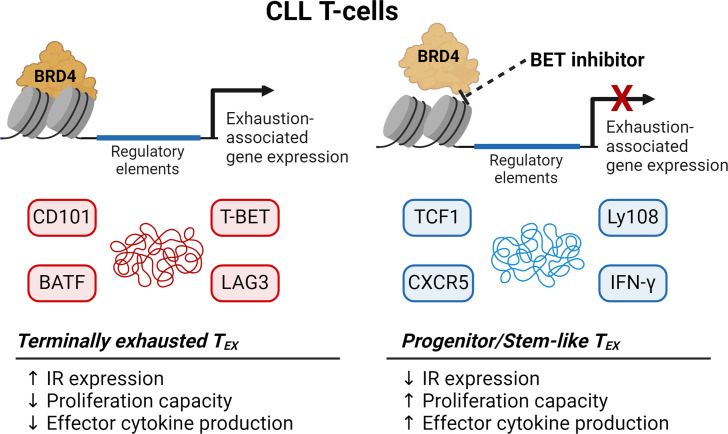

## Introduction

Chronic lymphocytic leukemia (CLL) B cells and tumor microenvironment (TME) bystander cells incite a tolerogenic environment through soluble factors (e.g., IL-10) and immune inhibitory molecules (e.g., PD-L1, LAG3) (1). Chronic antigen exposure in the TME promotes activation-induced T cell exhaustion accompanied by sustained immune inhibitory receptor (IR) expression and impaired proliferation, cytokine release, and immune synapse formation (2, 3). Consequently, CLL patient effector T cells are often hyporesponsive, permitting disease persistence and increased susceptibility to infection (4). Immunotherapies aimed to reinvigorate dysfunctional antitumor T cells have been minimally effective in CLL (5–7), potentially owing to long-lasting epigenetic scars associated with T cell exhaustion (8). Therefore, epigenetic interventions may be necessary to reprogram T cell exhaustion–associated circuits in CLL (9, 10).

Herein we investigate targeting bromodomain and extraterminal (BET) proteins (11) to alleviate CLL T cell dysfunction. BET proteins (e.g., BRD2, BRD3, BRD4) are chromatin readers that bind to acetylated lysine residues on histones and are enriched at active enhancer and promoter regions of target genes. CLL B cells overexpress BRD4 and have an increased BRD4 load at superenhancers of key genes involved in CLL pathogenesis and TME interactions (12–14). Notably, BRD4 regulates members of the B cell receptor (BCR) pathway (e.g., BTK, PI3K), oncogenic drivers (e.g., TCL1, MYC), mediators of immune function (e.g., PD-L1, LAG3), and TME interactions (e.g., CCR7, IL2R) in malignant B cells (12–14). Novel BET inhibitors have demonstrated selective anticancer activity and therapeutic efficacy in a variety of lymphoid malignancies, including CLL (12, 13, 15, 16).

In this study, we demonstrate that the pan-BET inhibitor, OPN-51107 (previously known as PLX51107) (12), reshapes the leukemia TME and alleviates CLL-induced T cell dysfunction. These data support the use of epigenetic modulation to simultaneously reduce leukemic cell burden and reinvigorate suppressed T cells in CLL.

## Results

### BET inhibition relieves immunosuppressive networks in the CLL microenvironment.

**Figure 1:**
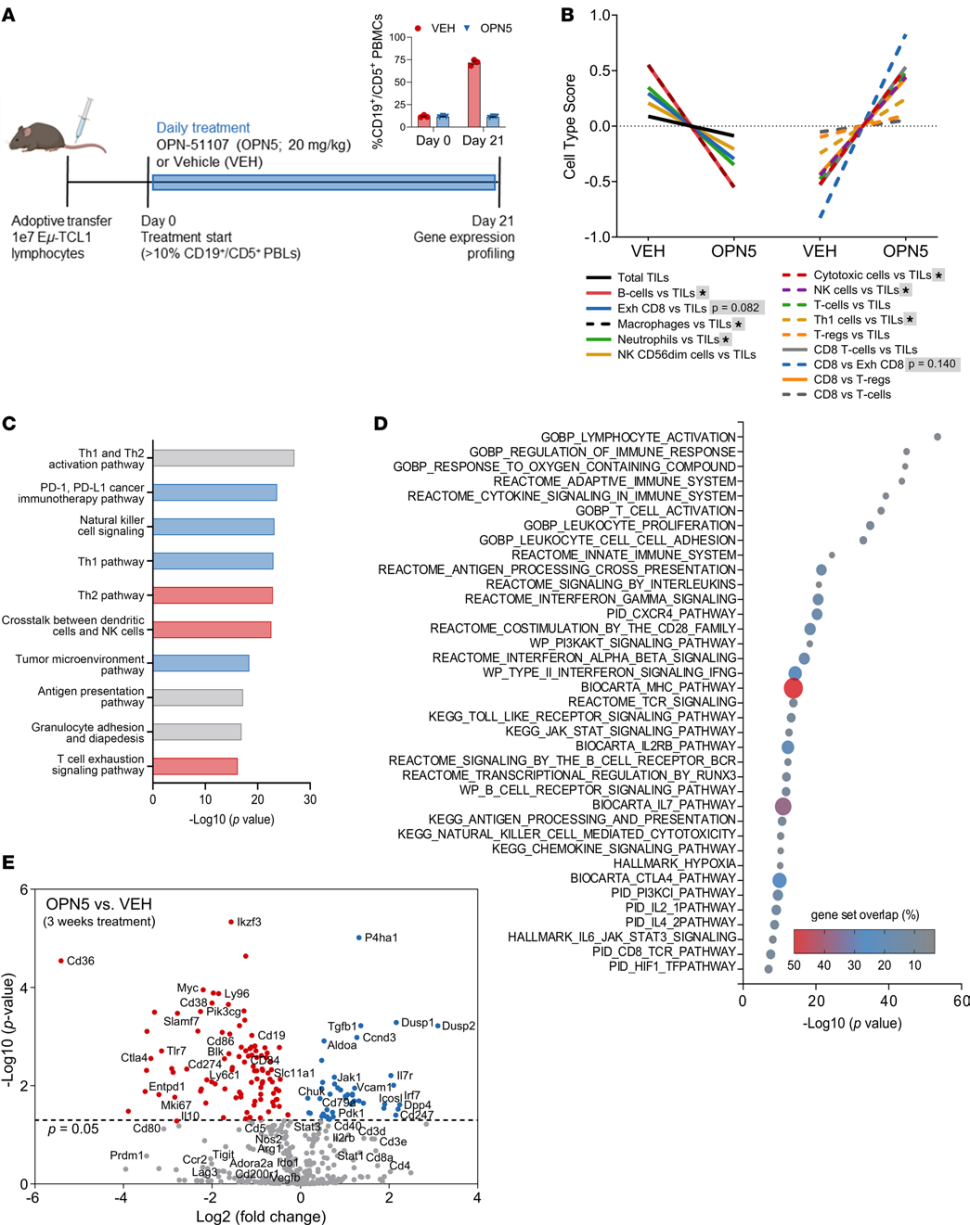
BET inhibition alters the CLL microenvironment immune profile. (**A**) Schematic of study design. WT B6 mice were engrafted via tail vein injection with lymphocytes derived from heavily diseased Eμ-TCL1 mice. Upon disease onset (≥10% CD45^+^CD19^+^CD5^+^ PBLs), recipient mice were randomly assigned to treatment with OPN-51107 (OPN5) or vehicle equivalent (VEH) for 21 days (*n* = 3/treatment group). Disease burden in the peripheral blood is shown. (**B**–**E**) NanoString PanCaner iO360 target gene expression analysis conducted on splenic tissue from treated mice. (**B**) NanoString cell type scoring indicating differences in immune cell populations after treatment. TILs, tumor-infiltrating lymphocytes; Exh, exhausted. Unpaired, 2-tailed Mann-Whitney *U* tests were used to determine significant differences between VEH and OPN5 groups for each cell type. *P < 0.05. (**C**) Significantly modulated canonical pathways identified by IPA following BET inhibition with OPN5. The direction of *Z* score is indicated by bar coloring: activated (blue), inhibited (red), no activity pattern available (gray). (**D**) MSigDB analysis of genes significantly modulated by OPN5 treatment (*P* < 0.05). Gene set overlap is defined as (the number of significantly modulated genes that fall in a pathway gene set/the total number of genes in that gene set) × 100. (**E**) Volcano plot of differentially expressed genes following BET inhibitor treatment.

OPN-51107 (OPN5) is a nonbenzodiazepine BET inhibitor, distinct from other available BET inhibitors in its capacity to engage the ZA channel (a specificity loop defined by αZ and αA helices) of BRD4, yielding structural plasticity (12). Through potent inhibition of BET-bromodomain (BET-BD) interactions, we previously reported that OPN5 significantly impairs CLL B cell proliferation and demonstrates profound antitumor effects in Eμ*-*TCL1 mice (12). However, the effect of this BET inhibitor on the CLL TME and immune dysfunction was not explored. To investigate broad effects of BET inhibition (BET-i) on the CLL TME, we employed the aggressive Eμ-TCL1 adoptive transfer (AT) model (17) (Figure 1A). Resembling the transgenic Eμ-TCL1 model, Eμ-TCL1 AT mice accumulate tumor cells in the spleen, generating an immunosuppressive microenvironment that is the primary site of T cell activation and subsequent exhaustion (18–20). Following engraftment with Eμ*-*TCL1 spleen-derived lymphocytes, mice were monitored until tumor expansion was witnessed (≥10% CD45^+^CD19^+^CD5^+^ peripheral blood lymphocytes [PBLs]), then randomly assigned to receive OPN5 or vehicle equivalent (VEH). After 21 days of treatment, mice displayed disparate disease levels in the peripheral blood (OPN5 [11.2% ± 0.5%] versus VEH [71.8 % ± 1.9%] CD19^+^CD5^+^ PBLs; Figure 1A). Total splenic tissue and peripheral blood mononuclear cells (PBMCs) revealed differentially expressed genes (DEGs) indicative of significant changes in cell composition (NanoString cell-type scores; Figure 1B and Supplemental Figure 1A; supplemental material available online with this article; https://doi.org/10.1172/jci.insight.177054DS1). OPN5-treated mice had a reduced splenic B cell score, likely due to reduced disease burden. Relative scores for cytotoxic, NK, and Th1 cells (spleen) or total T cells (PBMCs) increased, while the exhausted CD8^+^ T cell score decreased with OPN5 treatment (Figure 1B and Supplemental Figure 1A). Pathway analysis of splenic cells identified inhibition of T cell exhaustion signaling and activation of Th1, PD-1/PD-L1 cancer immunotherapy, NK cell, and IL-7 signaling pathways among the most significant effects predicted with BET-i (Figure 1C and Supplemental Table 2). Gene set enrichment analysis (GSEA) indicated that numerous immune signatures were modulated with OPN5 treatment, including IL-2, IL2RB, IFN-γ, T cell receptor (TCR) signaling, CD8^+^ TCR, T cell activation, and transcriptional regulation by RUNX3 pathways (Figure 1D and Supplemental Figure 1C).

We identified 244 splenic DEGs and 236 PBMC DEGs following OPN5 treatment (Figure 1E and Supplemental Figure 1D). As expected, genes highly expressed by CLL B cells and those driving their proliferation were downregulated in OPN5-treated mice (e.g., *Cd19*, *Mki67*, *Myc*, *Pik3cg*). Multiple genes contributing to T cell activation, including *Cd3e*, *Cd8a*, and *Icosl*, were upregulated in the spleen but not in PBMCs with BET-i (Supplemental Figure 2A and Supplemental Figure 3A). However, genes involved in impaired T cell activation like *Ctla4*, *Cd274* (PD-L1)*,* and *Tigit* were downregulated with BET-i in both tissues (Supplemental Figure 2B and Supplemental Figure 3B). Of note, genes that affect T cell differentiation (e.g., *Prmd1*, *Il7r*) (21–23) were significantly modulated with treatment (Supplemental Figure 2C and Supplemental Figure 3C). BET-i additionally altered genes commonly utilized by other TME immunosuppressive cells, such as myeloid-derived suppressor cells (MDSCs) (24, 25) (Supplemental Figure 2D and Supplemental Figure 3D). Correspondingly, MDSC populations were reduced in the peripheral blood and spleen of OPN5-treated mice and exhibited lower expression of inhibitory molecules (Supplemental Figure 4, A–D). Within the spleen, antiinflammatory “patrolling” monocytes and Tregs were also reduced with BET-i (Supplemental Figure 4, E and F). Together, these findings suggest that BET-i can influence the immune TME to reduce T cell suppression in CLL.

### BET-i rescues exhausted T cell function in vivo.

**Figure 2:**
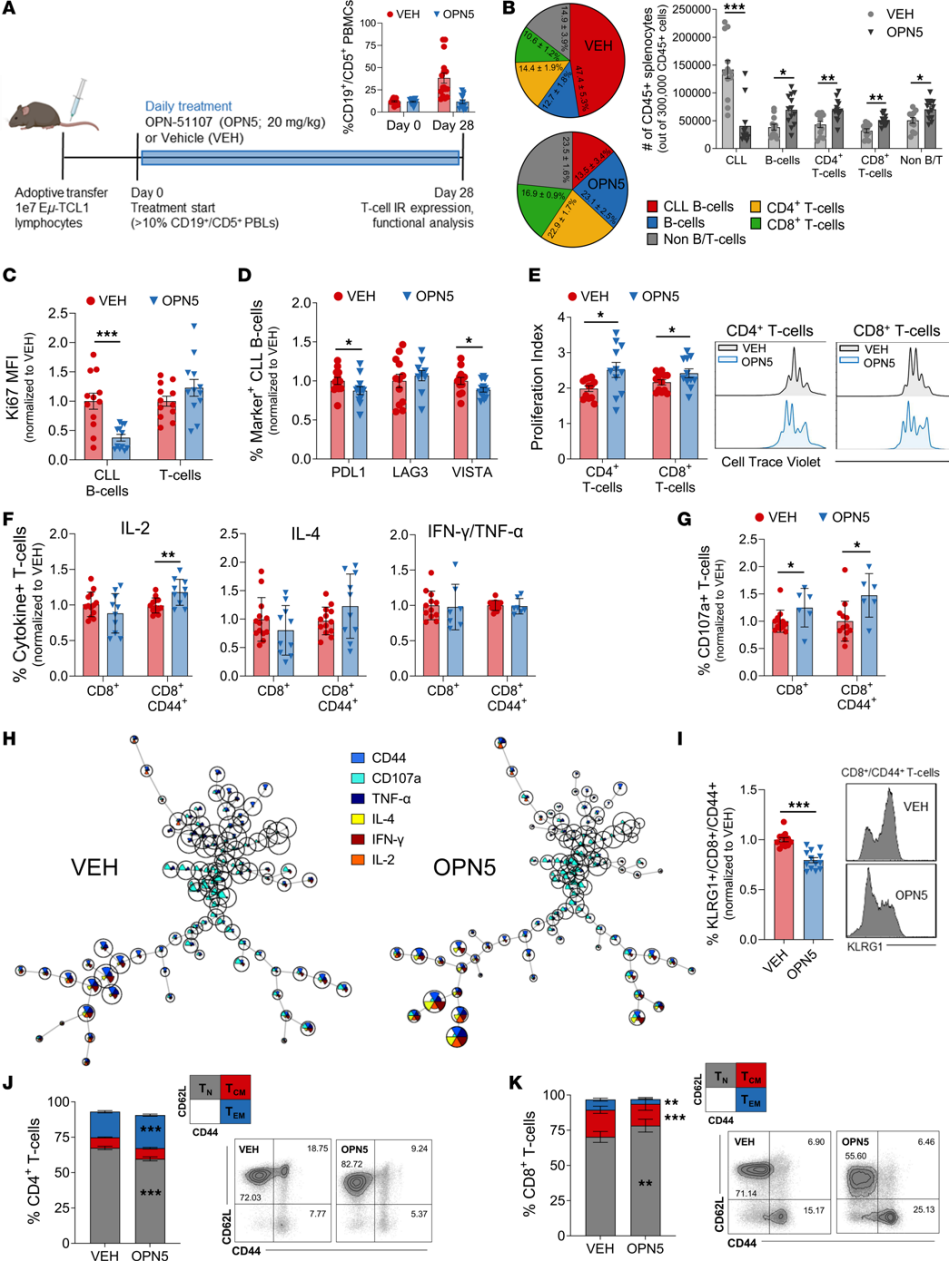
BET inhibition improves T cell function in an aggressive murine model of CLL. (**A**) Schematic of study design. Upon disease onset, recipient mice were randomly assigned to treatment with OPN-51107 (OPN5, *n* = 13) or vehicle equivalent (VEH, *n* = 12) for 28 days. Disease burden in the peripheral blood is shown. (**B**) Abundances of cell types found in the spleen. CLL B cells were gated as CD45^+^CD19^+^CD5^+^, other B cells were gated as CD45^+^CD19^+^CD5^–^, T cells were gated as CD45^+^CD19^–^/CD4^+^ or CD8^+^. (**C**) Ki67 expression in splenic CLL B cells and T cells evaluated by flow cytometry. MFI, median fluorescent intensity. (**D**) Percentages of CLL B cells expressing immune inhibitory receptors, normalized to the average of VEH-treated mice. (**E**) Proliferation indices of Cell Trace Violet–stained splenic T cells, stimulated ex vivo with anti-CD3/anti-CD28 for 72 hours. (**F** and **G**) Splenic T cells stimulated ex vivo for 6 hours with PMA/ionomycin and then evaluated by flow cytometry for percentages of T cells expressing intracellular cytokines (**F**) or membrane localized CD107a (**G**). (**H**) Representative FlowSOM clustering of splenic CD8^+^ T cells. Clustering is based on expression of CD44, CD107a, TNF-α, IL-4, IFN-γ, and IL-2. Relative expression is illustrated as the size of colored pie slices within each cluster. The relative abundance of each cluster is represented by cluster size. (**I**) Percentage of antigen-experienced (CD44^+^) CD8^+^ splenic T cells expressing KLRG1. (**J** and **K**) Distribution of CD4^+^ (**J**) and CD8^+^ (**K**) splenic T cells into naive (TN; CD44^–^CD62L^+^), central memory (TCM; CD44^+^CD62L^+^), and effector memory (TEM; CD44^+^CD62L^–^) subsets with representative flow cytometry plots. Asterisks denote significant differences between VEH and OPN5 for each T cell subset. Summary data are represented as mean ± SEM. Unpaired, 2-tailed Mann-Whitney *U* tests were used to determine significant difference between VEH and OPN5 groups. *P < 0.05, **P < 0.01, ***P < 0.001.

We conducted a second AT study to assess the function and differentiation status of splenic T cells from mice treated with OPN5. After 28 days of treatment, OPN5-treated mice displayed significantly less disease in the peripheral blood than VEH-treated mice (11.6% ± 2.1% versus 38.3% ± 5.8% CD19^+^CD5^+^ PBLs, respectively; Figure 2A). Spleens from OPN5-treated mice harbored markedly smaller proportions of CLL B cells and greater proportions of T cells (Figure 2B). Leukemic B cells from OPN5-treated mice were less proliferative (Figure 2C) and displayed reduced expression of PD-L1 and VISTA (Figure 2D), suggesting a diminished capacity to inhibit T cell function. Both CD4^+^ and CD8^+^ T cells from OPN5-treated mice demonstrated greater ex vivo proliferative capacity compared with VEH-treated mice (Figure 2E). Antigen-experienced CD8^+^ T cells from OPN5-treated mice produced more IL-2 upon mitogenic stimulation (Figure 2F) (26). Total and antigen-experienced CD8^+^ T cells from OPN5-treated mice additionally displayed increased CD107a membrane localization, indicative of cytotoxic degranulation (Figure 2G). Total CD4^+^ T cells from OPN5-treated mice had enhanced expression of both IFN-γ/TNF-α (Th1 cytokines) and IL-4 (Th2 cytokine) upon stimulation (Supplemental Figure 5A). To determine changes in cytokine coexpression with BET-i, stimulated T cells were analyzed using the FlowSOM algorithm, which clusters high-dimensional cytometry data through self-organizing maps (27). OPN5 treatment significantly increased the percentage of polyfunctional, antigen-experienced CD4^+^ and CD8^+^ T cells following ex vivo stimulation (i.e., T cells coexpressing CD44, TNF-α, IL-4, IFN-γ, and IL-2, plus CD107a for CD8^+^ cells) (Figure 2H; Supplemental Figure 5, B–D; and Supplemental Figure 6).

Patients with CLL progressively accumulate highly differentiated effector and memory T cell populations as well as short-lived effector cells (SLECs) with minimal memory capacity (28). Following BET-i, antigen-experienced CD8^+^ T cells expressed less KLRG1 (Figure 2I), a highly upregulated marker on SLECs (28) that mediates effector memory T cell (Tem) senescence (29). A lesser proportion of CD4^+^ splenic T cells from OPN5-treated mice were identified as naive (Tn) and a greater proportion were identified as Tem (Figure 2J). However, CD8^+^ T cells displayed obverse shifts in leukemic mice, with more Tn cells and less central memory (Tcm) and Tem cells following BET-i (Figure 2K), potentially due to reduced tumor antigen presence and/or clearance of SLECs (21).

**Figure 3:**
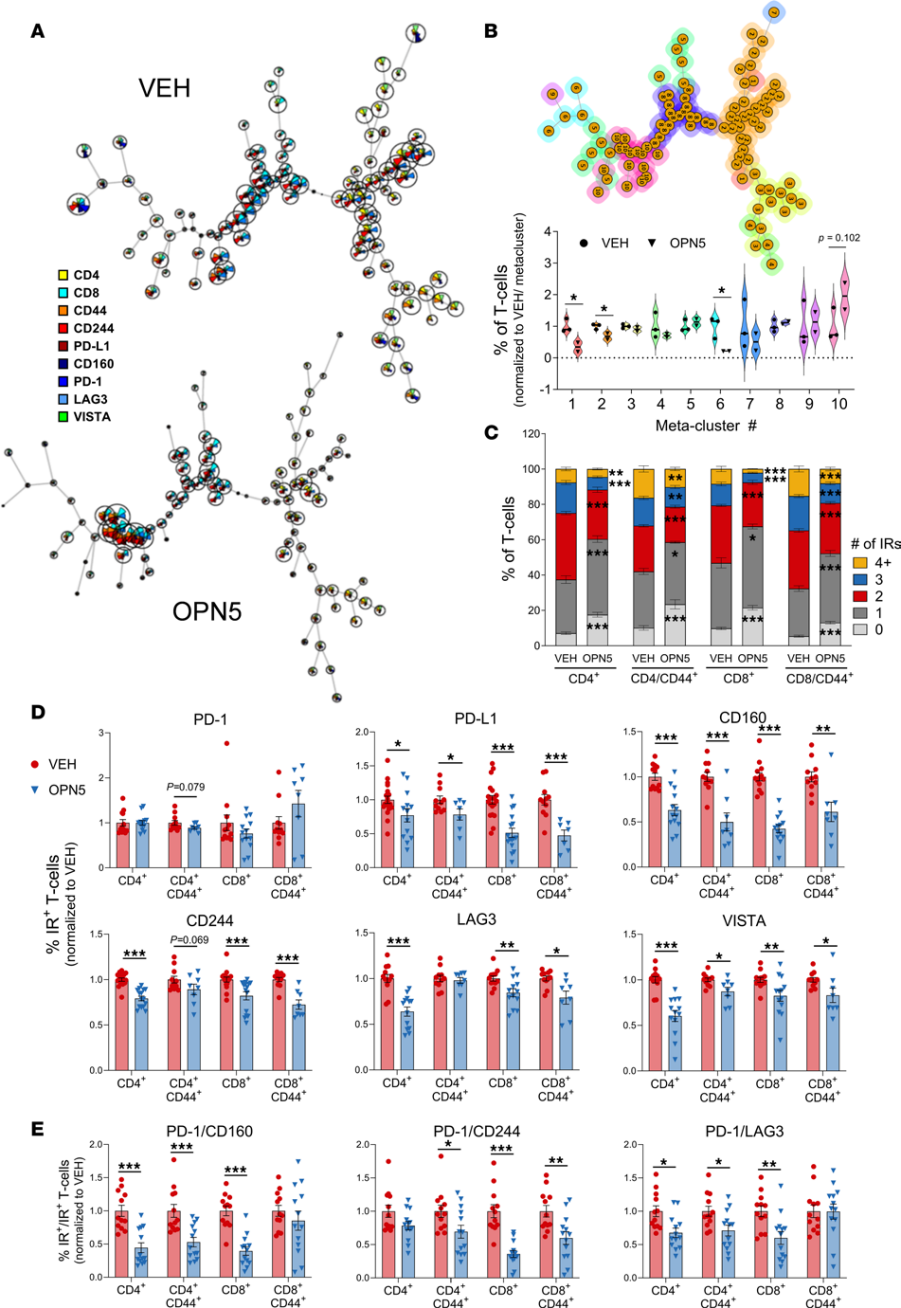
BET inhibition alleviates T cell exhaustion in an aggressive murine model of CLL. (**A**) Representative FlowSOM clustering of splenic T cells from adoptive transfer Eμ-TCL1 mice treated for 28 days with OPN-51107 (OPN5; *n* = 2) or vehicle equivalent (VEH; *n* = 3). Clustering is based on the expression of CD4, CD8, CD44, CD244, PD-L1, CD160, PD-1, LAG3, and VISTA detected via flow cytometry. CD4^+^ T cells are clustered on the right branch and CD8^+^ T cells on the left branch of representative star plots. Relative expression is illustrated as the size of colored pie slices within each cluster. The relative abundance of each cluster is represented by cluster size. (**B**) Violin plot demonstrating fold change in the percentage of T cells found in each FlowSOM-identified metacluster (line at median). Top insert illustrates representative metacluster numbering (#1–#10). (**C**) Number of evaluated immune inhibitory receptors (IRs) (PD-1, PD-L1, CD160, CD244, LAG3, VISTA) coexpressed on splenic T cells. Asterisks denote significant differences between VEH (*n* = 12) and OPN5 (*n* = 13) for each no. of IRs per T cell subset. (**D**) Percentages of splenic T cells expressing the indicated individual immune IRs, normalized to the average of VEH-treated mice. (**E**) Percentages of splenic T cells coexpressing PD-1 and CD160, CD244, or LAG3, normalized to the average of VEH-treated mice. Summary data are represented as mean ± SEM. Unpaired, 2-tailed Mann-Whitney *U* tests were used to determine significant difference between VEH and OPN5 groups. *P < 0.05, **P < 0.01, ***P < 0.001.

High IR expression in patients with CLL is associated with T cell exhaustion, advanced disease, and therapy resistance (30, 31). OPN5 treatment reduced the overt severity of T cell dysfunction in mice, evidenced by the diminished percentage of splenic CD8^+^ T cells coexpressing CD44, PD-1, PD-L1, VISTA, CD244 (SLAMF4), CD160, and LAG3 (FlowSOM-identified metacluster #6; Figure 3, A and B, and Supplemental Figure 7). CD4^+^ T cell populations coexpressing CD44, PD-L1, and CD244 or VISTA were also markedly lessened with BET-i (metaclusters #1 and #2; Figure 3, A and B, and Supplemental Figure 7). Across T cell subsets, significantly more T cells from OPN5-treated mice expressed zero or 1 IR, while fewer coexpressed 2 or more (Figure 3C). When interrogating individual markers, BET-i reduced PD-L1, CD160, CD244, LAG3, and VISTA expression on most T cell subsets (Figure 3D). PD-1 expression alone was not modulated with OPN5 treatment in AT mice (Figure 3D), yet its coexpression with other IRs is commonly a prerequisite for classifying exhausted T cells (32). OPN5-treated mice had fewer T cells coexpressing PD-1 with CD160, CD244, or LAG3 (Figure 3E), suggesting increased potential to mount effective antitumor responses following BET-i.

We confirmed these results in the Eμ-TCL1 transgenic mouse model, which spontaneously develops CD19^+^CD5^+^ CLL-like disease and recapitulates immune dysfunction witnessed in patients (17, 18). Diseased Eμ-TCL1 mice (average age 11.6 months) were treated with OPN5 or VEH for 7 days (Supplemental Figure 8A). After this short treatment, OPN5 reduced leukemic burden in the peripheral blood and spleen (Supplemental Figure 8, B and C). Splenic CLL B cells from OPN5-treated mice displayed reduced PD-L1 expression (Supplemental Figure 8D). Like the syngeneic AT model, fewer Tn CD4^+^ T cells and more Tem CD4^+^ T cells were observed in spleens of OPN5-treated Eμ-TCL1 mice (Supplemental Figure 8E). No subset differences were observed for CD8^+^ T cells in this short-term study (Supplemental Figure 8F). In OPN5-treated mice, a greater proportion of T cells expressed zero or 1 IR, while significantly fewer coexpressed 3 or more (Supplemental Figure 8, G and H), suggesting that BET proteins tightly regulate IR expression on T cells in the leukemic microenvironment. Overall, BET-i alleviated signs of T cell exhaustion in both Eμ-TCL1 AT mice and aged Eμ-TCL1 transgenic mice with advanced disease.

### BET-i repairs T cell dysfunction induced by patient-derived CLL cells.

**Figure 4:**
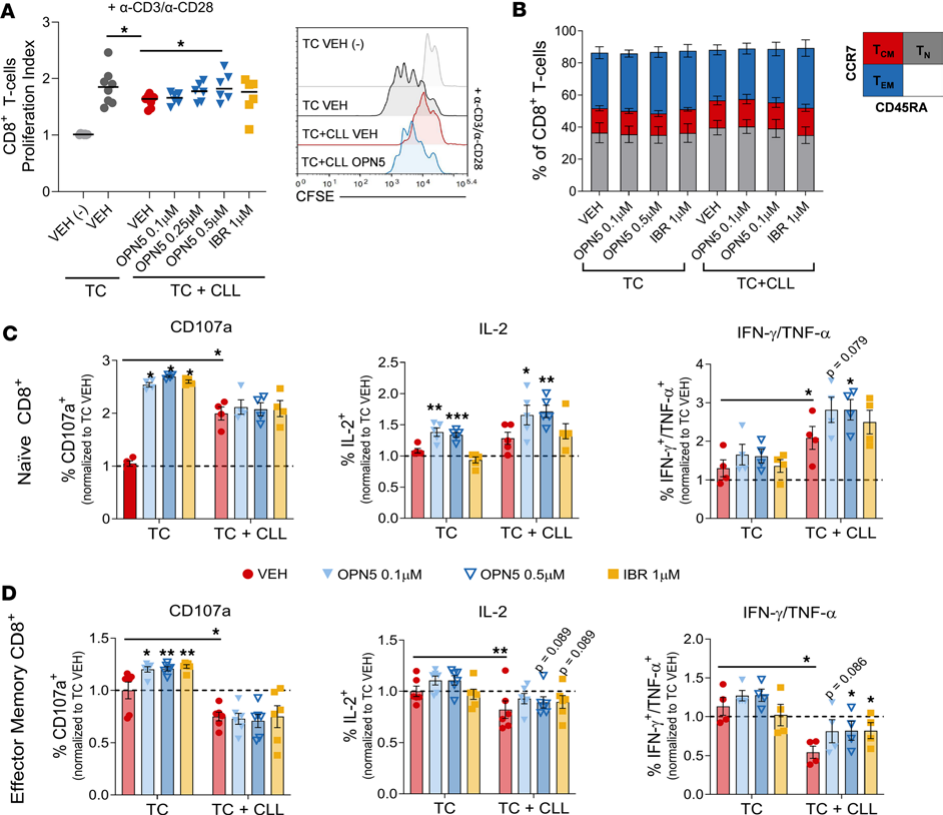
BET inhibition enhances the functionality of CLL-suppressed T cells ex vivo. (**A**) Flow cytometry proliferation analysis of CFSE-stained healthy donor CD8^+^ T cells cultured alone (TC) or cocultured with patient-derived CLL B cells (TC + CLL) in the presence of CXCL12, stimulated with anti-CD3/anti-CD28, and treated with the indicated inhibitors for 96 hours (*n* = 6–8). (**B**) Distribution of basal CD8^+^ T cells into naive (Tn; CD45RA^+^CCR7^+^), central memory (Tcm; CD45RA^–^CCR7^+^), and effector memory (Tem; CD45RA^–^CCR7^–^) subsets. TC + CLL = 48-hour coculture (*n* = 4–6). (**C** and **D**) Healthy donor T cells cultured alone or cocultured with patient-derived CLL B cells and treated with the indicated inhibitors for 48 hours in the presence of 3.2 μM CpG ODN 2006, with PMA/ionomycin stimulation for the final 6 hours (*n* = 4–6). Percentages of CD107a^+^, IL-2^+^ and IFN-γ^+^TNF-α^+^ Tn (**C**) and Tem (**D**) CD8^+^ T cells upon PMA/ionomycin stimulation, normalized to VEH-treated T cell monoculture average. Summary data are represented as mean ± SEM. VEH, vehicle (DMSO); OPN5, OPN-51107; IBR, ibrutinib. Significant difference from VEH was calculated using 1-way ANOVA. *P < 0.05, **P < 0.01, ***P < 0.001.

To validate the ability of BET-i to reverse T cell dysfunction elicited by patient CLL B cells, we utilized an ex vivo coculture system as previously described (3). The BTK inhibitor, ibrutinib (IBR), acted as a control CLL therapeutic, given its known ability to repair T cell defects in CLL (33). To evaluate T cell proliferation, cultures were stimulated with anti-CD3/anti-CD28 in the presence of CXCL-12, which is known to induce CLL B cell IL-10 production (34). Cocultures treated with 0.5 μM OPN5 displayed marked alleviation of CLL-induced immunosuppression with improved CD8^+^ T cell proliferation (Figure 4A).

To investigate T cell effector function, cocultures were treated with OPN5 or IBR in the presence of CpG ODN, a TLR9 agonist known to mimic proliferation and immunostimulatory signals observed in CLL proliferation centers (35). In the last 6 hours of culture, cells were additionally stimulated with phorbol 12-myristate 13-acetate (PMA) and ionomycin, an experimental cocktail commonly used to incite T cell cytokine production but not indicative of physiologic antigen simulation. No changes in T cell subset distribution were witnessed in 48-hour cocultures (Figure 4B). Unsupervised clustering of T cell cytokine expression demonstrated that CLL coculture impaired polyfunctional CD8^+^ T cells, and this effect was alleviated with OPN5 treatment (Supplemental Figure 9). Upon mitogenic stimulation, monocultured Tn cells displayed increased CD107a membrane localization with OPN5 or IBR, but only OPN5 treatment increased IL-2 and dual INF-γ/TNF-α expression in mono- and cocultured Tn cells (Figure 4C). CLL-induced suppression of Tem IL-2 and dual IFN-γ/TNF-α expression was additionally assuaged with OPN5 (Figure 4D). Although not altered on cocultured Tem cells, CD107a membrane localization was significantly enhanced on monocultured Tem cells with either treatment (Figure 4D). These results attest that OPN5 is not detrimental to healthy T cell function and that its proinflammatory effect on CLL T cells is not solely dependent on reducing leukemic B cell burden.

BET-i also lessened the percentage of minimally functional CD4^+^ T cells and increased the percentage of CD4^+^ T cells exhibiting a polyfunctional cytokine response to stimuli (Supplemental Figure 10). CD4^+^ T cells from CLL cocultures treated with OPN5 displayed enhanced proliferation and production of IL-2 and IFN-γ/TNF-α (Supplemental Figure 11). These findings indicate that BET-i can impart a proinflammatory effect on both CD8^+^ and CD4^+^ T cells.

### BET-i promotes a stem-like T cell phenotype in ex vivo CLL models.

**Figure 5:**
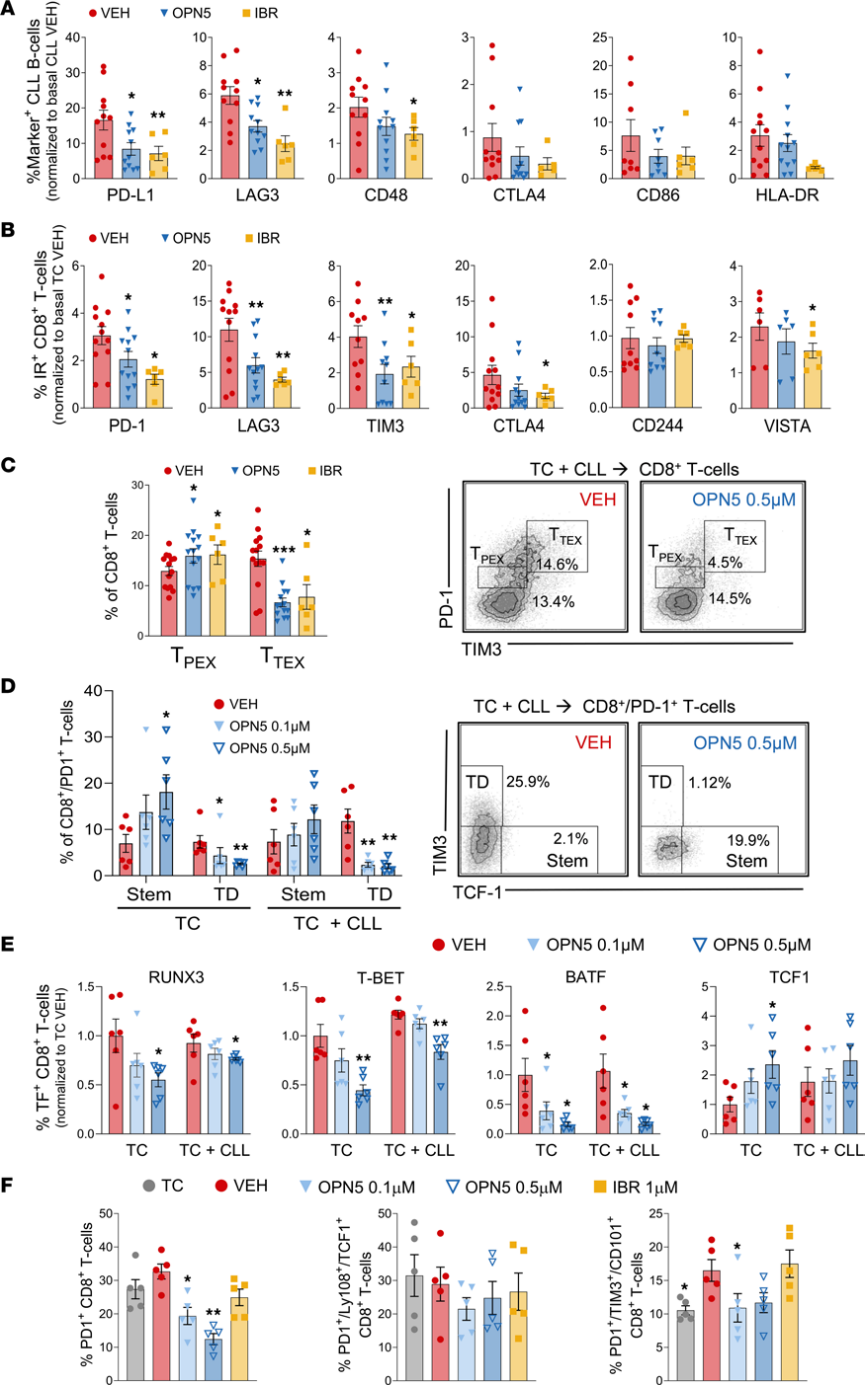
BET inhibition reduces the expression of inhibitory immune molecules on CLL B cells and T cells ex vivo. CLL B cell/healthy donor T cell cocultures were incubated for 48 hours with anti-CD3/anti-CD28 stimuli and OPN-51107 (OPN5; 0.1 μM or 0.5 μM), ibrutinib (IBR; 1 μM), or vehicle equivalent (VEH; DMSO); they were then evaluated by flow cytometry for expression of the indicated markers. (**A**) Percentages of cocultured CLL B cells expressing immune inhibitory or stimulatory molecules or receptors, normalized to VEH-treated CLL B cell monoculture average (*n* = 6–11). (**B**) Percentages of cocultured CD8^+^ T cells expressing the indicated immune inhibitory receptors (IRs), normalized to VEH-treated T cell monoculture average (*n* = 6–12). (**C**) Distribution of cocultured CD8^+^ T cells into progenitor exhausted (T_PEX_; PD-1^int^/TIM3^lo/–^) and terminally exhausted (T_TEX_; PD-1^hi^/TIM3^hi^) T cell subsets (*n* = 6–14). Data are represented as mean ± SEM. (**D**) Percentages of stem-like (Stem; PD-1^+^TIM3^–^TCF1^+^) and terminally differentiated (TD; PD-1^+^TIM3^+^TCF1^–^) CD8^+^ T cells in treated T cell monocultures (TC) and CLL B cell cocultures (TC + CLL) (*n* = 6). (**E**) Percentages of CD8^+^ T cells expressing RUNX3, T-BET, BATF, and TCF1, normalized to TC VEH average (*n* = 6). (**F**) Percentages of PD-1^+^, PD-1^+^TCF1^+^Ly108^+^ (progenitor/stem-like exhausted), and PD-1^+^TIM3^+^CD101^+^ (terminally exhausted) CD8^+^ T cells (*n* = 5). Significant difference from VEH was calculated using 1-way ANOVA. *P < 0.05, **P < 0.01, ***P < 0.001.

We next evaluated IR expression in ex vivo cocultures following BET-i. Coculture of healthy donor T cells with CLL patient B cells evoked elevated IR expression within 48 hours (Supplemental Figure 12A). Surface levels of PD-L1, LAG3, CD48 (SLAMF2), and CTLA4 were reduced on CLL B cells in cocultures treated with OPN5 or IBR (Figure 5A). HLA-DR expression on CLL B cell was markedly decreased by IBR but not OPN5 (Figure 5A). Notably, BET-i has recently been shown to enhance HLA expression in other settings (36). OPN5 significantly downregulated PD-1, LAG3, and TIM3 expression on CD8^+^ and CD4^+^ T cells akin to IBR (Figure 5B and Supplemental Figure 10A) and evoked a nonsignificant decrease in CTLA4, CD244 (SLAMF4), and VISTA expression (Figure 5B and Supplemental Figure 13A).

There are 2 major subsets of exhausted T cells with distinct potential for future immune responses; PD-1^int^/TIM3^lo/-^ T cells are referred to as progenitor exhausted T cells (T_PEX_), and PD-1^hi^/TIM3^hi^ T cells are deemed terminal exhausted T cells (T_TEX_) (32, 37, 38). Both OPN5 and IBR significantly reduced the percentage of CD4^+^ and CD8^+^ T_TEX_ cells, while increasing the percentage of CD8^+^ T_PEX_ cells in ex vivo cocultures (Figure 5C and Supplemental Figure 13B). We reasoned that the immunomodulatory effects of BET-i may be partially due to altered expression of key transcription factors (TFs) dictating T cell stemness (TCF1) versus terminal differentiation (BATF, RUNX3, T-BET). TCF1 is a critical TF for maintenance of T_PEX_ cells, which have potential for reinvigoration upon checkpoint blockade (38–40). When TCF1 expression was incorporated to define stem-like versus terminally differentiated T cells (8, 38), we found that mono- or cocultured healthy donor CD8^+^ T cells treated with OPN5 were more stem-like and less terminally differentiated (Figure 5D). Moreover, OPN5 reduced expression of TFs driving terminal effector differentiation and increased TCF1 expression in mono- and cocultured CD8^+^ T cells (Figure 5E). Similar trends were witnessed for CD4^+^ T cells (Supplemental Figure 13, C and D). To confirm our results from allogenic cocultures, we evaluated the immunomodulatory capacity of BET-i in patient-derived PBMCs. Herein, OPN5 treatment analogously enriched for stem-like CD8^+^ T cells and reduced expression of terminal differentiation-associated TFs (Supplemental Figure 14). Moreover, single-agent IBR was incapable of alleviating CD8^+^ T cell differentiation skewing (Supplemental Figure 14), indicating that improved stem-like function may be a T cell–specific effect of BET-i.

Exhausted T cell state can be further detailed by the expression of Ly108 (SLAMF6) and CD101 (41–43). Ly108^+^TCF1^+^ progenitor/stem-like T cells were relatively unchanged with OPN5 or IBR treatment, yet TIM3^+^CD101^+^ T_TEX_ cells were increased with CLL coculture and reduced with OPN5 but not with IBR treatment (Figure 5F). Maintained GZMB production is also associated with late exhaustion differentiation (38, 43). CD8^+^ T cell GZMB expression was consistently reduced in OPN5-treated cocultures (Supplemental Figure 12G). Notably, extended stimulation of healthy donor T cells resulted in the accumulation of terminally differentiated CD8^+^ T cells (Supplemental Figure 12C), negating the initial immunosuppressive effect of CLL coculture. However, OPN5 still reduced the percentage of terminally differentiated CD8^+^ T cells in cocultures stimulated and treated for 7 days (Supplemental Figure 12C). Pretreatment of CLL cells alone with OPN5 (no drug during coculture) was not sufficient to maintain OPN5’s effects on T cell differentiation, further implicating direct T cell effects of OPN5 treatment in addition to reduced CLL immunosuppressive signals (Supplemental Figure 12, E and F). This result was supported by FlowSOM clustering of live CD8^+^ T cells from cocultures based on expression of CD44, CD62L, CD127, KLRG1, IFN-γ, TNF-α, GZMB, PD-1, TIM3, TCF1, Ly108, and CD101 (Supplemental Figure 15). Metaclusters rich in CD44, Ly108, CD101, and GZMB were enriched with CLL coculture and depleted with continuous OPN5 treatment (Supplemental Figure 15B), but this effect was not maintained with CLL cell OPN5 pretreatment (Supplemental Figure 15C).

### BET-i directly resolves exhaustion in CLL patient T cells.

**Figure 6:**
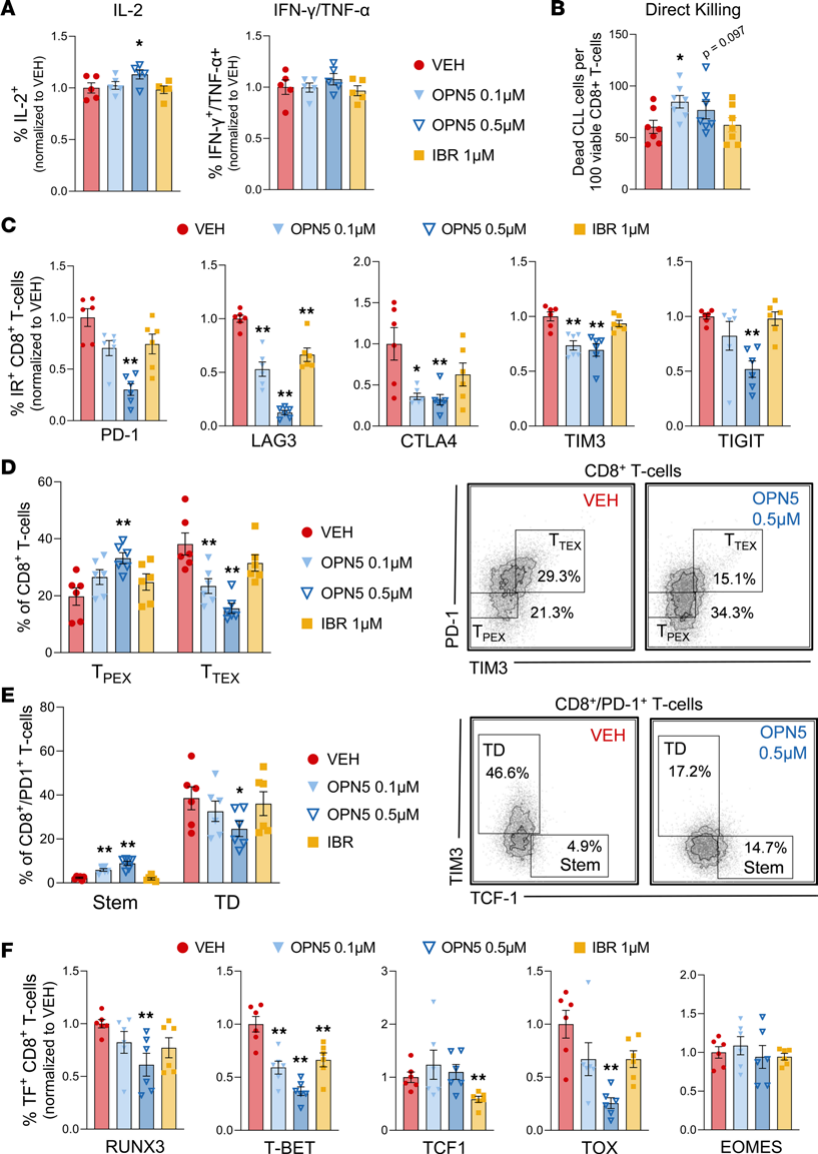
BET inhibition directly influences CLL patient–derived T cell function and state. (**A**) CLL patient–derived T cells treated with the OPN-51107 (OPN5; 0.1-0.5 μM), ibrutinib (IBR; 1 μM), or vehicle equivalent (VEH; DMSO) for 48 hours, stimulated with PMA/ionomycin for the final 6 hours before being evaluated by flow cytometry for percentage of IL-2^+^ and IFN-γ^+^TNF-α^+^ CD8^+^ T cells, normalized to VEH (*n* = 6). (**B**) CLL patient–derived CD8^+^ T cells treated with the indicated inhibitors for 48 hours before being cocultured with anti-CD3 coated MEC-1 CLL cells (*n* = 7). The number of dead MEC-1 cells per 100 viable CD8^+^ T cells following 18 hours of coculture is shown. (**C**–**F**) CLL patient–derived T cells treated with the indicated inhibitors in the presence of anti-CD3/anti-CD28 stimuli for 48 hours (*n* = 6). (**C**) Percentages of CD8^+^ T cells expressing immune inhibitory receptors (IRs) PD-1, LAG3, CTLA4, TIM3, and TIGIT, normalized to VEH. (**D**) Distribution of CD8^+^ T cells into progenitor exhausted (T_PEX_; PD-1^int^/TIM3^lo/–^) and terminally exhausted (T_TEX_; PD-1^hi^/TIM3^hi^) subsets. (**E**) Percentages of stem-like (Stem; PD-1^+^TIM3^–^TCF1^+^) and terminally differentiated (TD; PD-1^+^TIM3^+^TCF1^–^) CD8^+^ T cells. (**F**) Percentages of CD8^+^ T cells expressing transcription factors (TFs) EOMES, RUNX3, T-BET, TCF1, and TOX, normalized to VEH. Data are represented as mean ± SEM. Significant difference from VEH was calculated using 1-way ANOVA. *P < 0.05, **P < 0.01.

To verify T cell intrinsic effects of OPN5, T cells isolated from patients with CLL were treated ex vivo with OPN5 or IBR in the presence of activating stimuli. OPN5 enhanced both CD8^+^ and CD4^+^ T cell production of IL-2 and IFN-γ/TNF-α (Figure 6A and Supplemental Figure 16, A and B). The antitumor cytotoxic function of T cells was evaluated using a recently established TCR-dependent tumor-killing assay (44). CD8^+^ T cells derived from CLL patient–derived CD8^+^ T cells that were pretreated with were cocultured with MEC-1 cells in the presence of anti-CD3, which coats Fc receptors on tumor cells to facilitate TCR cross-linking. OPN5-treated T cells were more cytotoxic toward MEC-1 cells compared with VEH-treated or IBR-treated T cells (Figure 6B), suggesting that BET-i directly enhances CLL patient–derived CD8^+^ T cell antitumor capacity.

BET-i also directly affected exhaustion-associated IR and TF expression in T cells from patients with CLL. OPN5 significantly reduced IR expression, induced a shift from T_TEX_ to T_PEX_ (Figure 6, C and D, and Supplemental Figure 16, C and D), and altered the TF profile of T cells from patients with CLL to enrich for more stem-like populations (Figure 6, E and F, and Supplemental Figure 16, E and F). Herein, we expanded our TF panel to include TOX and EOMES. Sustained TOX expression is associated with terminal T cell exhaustion (45) and was markedly reduced with OPN5 treatment. EOMES is highly expressed in exhausted T cells but is required for CLL T cell antitumor function (46). No significant change in EOMES expression was witnessed with OPN5 treatment. In ChIP-Seq analyses from a recent study investigating BET-i in acute graft-versus-host disease (GVHD) (47), BRD4 was enriched at regulatory regions of *RUNX3*, *PRDM1* (BLIMP1), and *BATF* in healthy donor T cells and enrichment was reduced following treatment with 10 nM OPN-2853 (approximately equivalent to 0.1 μM of OPN5; Supplemental Figure 17) (15). BRD4 enrichment was minimal at the *TCF7* (TCF1), *TBX21* (T-BET), and *EOMES* gene loci (data not shown), suggesting insignificant direct BRD4 regulation in healthy donor T cells.

Moreover, the T cell effects witnessed with BET-i therapy in advanced disease Eμ-TCL1 mice were not observed with fludarabine (purine analog antimetabolite), a chemotherapy drug that, prior to the influx of targeted therapies, was commonly used for the treatment of CLL (48) (Supplemental Figure 18). Although both OPN5 and fludarabine reduce leukemic B cell burden (Supplemental Figure 18B), only targeted BET-i with OPN5 alleviated T cell dysfunction in vivo. OPN5, but not fludarabine, enriched for naive CD8^+^ T cells, reduced IR expression, and altered the TF profile of splenic CD8^+^ T cells to drive a more stem-like phenotype in Eμ-TCL1 mice (Supplemental Figure 18, C–F). These results support direct T cell effects mediated by BET-i in CLL.

### BET-i alleviates exhaustion-associated chromatin accessibility in CLL T cells.

**Figure 7:**
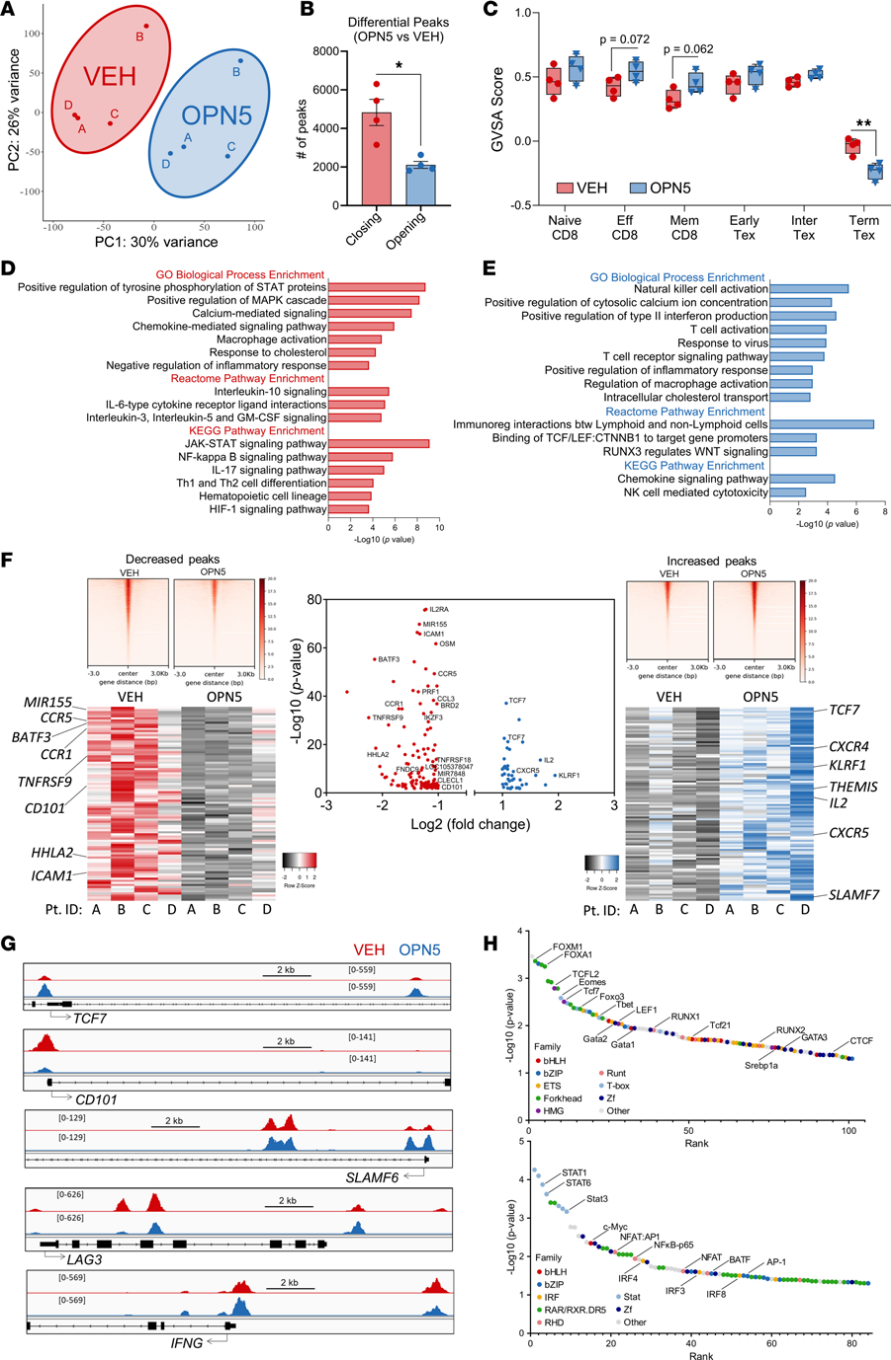
CD8^+^ T cell exhaustion–associated chromatin organization is altered with BET-i. (**A**–**H**) Bulk ATAC-Seq of CD8^+^ T cells isolated from CLL patient PBMCs (*n* = 4) that were treated with 0.5μM OPN-51107 (OPN5) or equivalent vehicle (VEH; DMSO) for 16 hours, in the presence of anti-CD3/anti-CD28 stimuli. (**A**) Principal component analysis (PCA) of BPM normalized signal at consensus peaks. (**B**) Differential peaks (*P* < 0.05) called in grouped OPN5 versus VEH analysis. Closing indicates peaks with decreased signal upon OPN5 treatment; opening indicates peaks with increased signal upon OPN5 treatment. Significant difference between groups was assessed using unpaired, 2-tailed Mann-Whitney *U* test. *P < 0.05. (**C**) Enrichment of T cell signatures based on ATAC-Seq data from Satpathy et al. (52). Data are represented as mean ± SEM. Significant difference between groups was assessed using unpaired Mann-Whitney *U* test or paired Student’s *t* test. **P < 0.01. (**D** and **E**) Overrepresentation analysis of gene ontology terms associated with genes near accessibility peaks that decreased (**D**) and increased (**E**) upon OPN5 treatment. (**F**) Differential promoter peaks identified in grouped OPN5 versus VEH analysis (volcano plot). Top heatmaps illustrate ATAC-Seq signal at consensus peaks between 3 or more patients for peaks decreased (left) and increased (right) upon OPN5 treatment. Bottom heatmaps display variation across CLL patient samples for differential promoter peaks identified in grouped analysis. (**G**) Chromatin accessibility derived from ATAC-Seq signal tracks at *TCF7*, *CD101*, *SLAMF6*, *LAG3*, and *IFNG* loci for grouped OPN5 and VEH samples. (**H**) Differentially accessible transcription factor binding motifs at increased (top) and decreased (bottom) peaks upon OPN5 treatment. Transcription factor families are annotated by color.

T cell exhaustion is maintained through a chromatin landscape that prevents memory or effector T cell function (8). While BET proteins are most widely recognized for their function in transcriptional elongation, they also play a significant role in chromatin remodeling (49–51). To further examine how BET-i alleviates CLL-associated T cell exhaustion through chromatin modulation, the assay for transposase-accessible chromatin with high-throughput sequencing (ATAC-Seq) was performed on CD8^+^ T cells isolated from isolated from CLL patient PBMCs that were treated with 0.5 μM OPN5 or VEH in the presence of anti-CD3/anti-CD28 for 16 hours. Principal component analyses (PCA) confirmed that OPN5 treatment induced global changes to chromatin accessibility compared with VEH controls (Figure 7A). Upon OPN5 treatment, an average of 4,830 loci showed a significant (FDR < 0.05) decrease in accessibility (closing), and 2,110 loci showed a significant (FDR < 0.05) increase (opening; Figure 7B). When this data set was compared with T cell signatures identified by Satpathy et al. (52) and Andreatta et al. (53), OPN5-treated cells displayed a reduced terminal exhaustion–like epigenetic signature compared with VEH-treated cells (Figure 7C and Supplemental Figure 19A). Gene ontology (GO) overrepresentation analysis of differentially accessible promoters indicated that OPN5 resulted in decreased accessibility of genes involved in the positive regulation of tyrosine phosphorylation of STAT proteins, IL-10 signaling, and NF-κB signaling (Figure 7D). Genes with increased accessibility are associated with NK cell and T cell activation, as well as the binding of TCF/LEF:CTNNB1 to target gene promoters (Figure 7E). Out of the thousands of differentially accessible loci (Figure 7B and Figure 7F, top heatmaps), analysis of OPN5- versus aggregate VEH-treated samples revealed 137 and 41 promoter regions with significantly decreased or increased accessibility, respectively (Figure 7F, middle volcano plot). The degree of changes at these promoters varied between patients (Figure 7F, bottom heatmaps). This analysis identified enhanced chromatin accessibility upon OPN5 treatment at the promoter regions of *TCF7* and *CXCR5* (Figure 7F and Supplemental Figure 19B). Conversely, reduced chromatin accessibility with OPN5 treatment was witnessed at the promoter regions of numerous genes in the terminal exhaustion epigenetic signature (52) (e.g., *CD101*, *BATF3*, *CCR5*, *TNFRSF9*; Figure 7F and Supplemental Figure 19B). This reversal of terminal exhaustion–associated chromatin organization with OPN5 treatment can be visualized in aggregate enhanced accessibility at *TCF7*, *SLAMF6*, and *IFNG* gene loci and in reduced accessibility at *CD101* and *LAG3* gene loci (Figure 7G). Motif analysis of differentially accessible sites identified Forkhead, HMG, T-box, Rnt, and ETS families of TF DNA-binding motifs with increased accessibility upon OPN5 treatment, while Stat, RHD, and IRF family binding motifs had decreased accessibility (Figure 7H). These data suggest that OPN5 treatment induces a unique epigenetic state that supports effector memory T cell function while reversing terminal exhaustion differentiation.

## Discussion

In this study, we characterized immunomodulatory effects of BET-i in preclinical models of CLL using OPN-51107, a pan-BET inhibitor in phase I/II trials (NCT04910152, NCT04022785; clinicaltrials.gov). We demonstrated that BET-i amends the immune microenvironment in CLL to mitigate T cell dysfunction beyond that expected of reduced tumor burden alone. In Eμ-TCL1 AT mice, BET-i reduced expression of IRs commonly targeted by checkpoint blockade (e.g., PD-1/PD-L1, LAG3, CTLA4) along with factors adding to redundant T cell suppression in CLL (e.g., CD244, *Il10*) and genes utilized by other immunosuppressive cellular contributors (e.g., *Cd84*, *Entpd1*, *Adora2a*) (54). Reduced antiinflammatory and/or immunosuppressive signatures of nonleukemic TME cellular components like MDSCs may, therefore, contribute to enhanced T cell function witnessed with OPN5 treatment in vivo. The antitumor effects of BET-i have been shown to, at least partially, rely on T cell presence in other malignancies (55, 56); however, experiments using T cell–deficient CLL models are needed to decipher the therapeutic effect of improved T cell function with OPN5 treatment in this setting. Additionally, a key limitation of this work is that it does not discern the tumor specificity of T cells. There is evidence for non–cancer-specific T cell exhaustion in the Eμ-TCL1 mouse model (57), but further studies are needed to determine the antigen specificity and, therefore, antitumor potential of CD8^+^ T cells following OPN5 treatment.

Recent work indicates that BRD4 plays a considerable role in differentiation and maintenance of terminal effector CD8^+^ T cells during viral infection through tight control of terminal effector–specific super-enhancers (58). In agreement, we found that BET-i increased the percentage of naive CD8^+^ T cells in Eμ-TCL1 AT mice and shifted CLL patient T cells to a progenitor phenotype in allogenic cocultures and patient-derived samples. Notably, the 2 exhausted CD8^+^ T cell subsets (progenitor/stem-like versus terminal) reported in this study are simplified versions of at least 4 subsets identified in the sequence of T cell exhaustion (41). Two TCF1^+^ progenitor subsets are distinct in proliferation state and circulatory presence, and progressive loss of TCF1 expression can yield an “effector-like” intermediate state prior to terminal exhaustion (41). The TFs we evaluated (e.g., TCF1, T-BET, TOX) coordinate the interplay of these exhausted CD8^+^ T cell subsets, but their expression in tumor-associated T cells can vary in different microenvironments (41, 46). ATAC-Seq analysis of CD8^+^ T cells from patients with CLL revealed that Forkhead, HMG-box, Runt, and T-box TF binding motifs were enriched with OPN5 treatment. FOXO1 was recently identified as a pioneer TF, responsible for promoting memory programs and preventing exhaustion in CAR T cells (59). The HMG family contains multiple TFs that dictate T cell differentiation, such as TCF1 (40) and TOX (45). Only binding motifs for HMG-box TFs that confer self-renewal potential were enriched with OPN5 treatment in our analysis (TCF1, TCFL2, LEF1, Sox17) (60). Interestingly, Runt-related TFs (RUNX) and T-box family TFs (e.g., T-BET) were reduced on the protein level with OPN5 treatment (Figure 5E and Figure 6F), yet accessible binding motifs for these TFs were enriched with OPN5 treatment (Figure 7H), indicating a sustained capacity for effector differentiation (61). Binding motifs depleted with OPN5 treatment also included those associated with immune activation (Stat, IRF, and RHD families) (62–65) as well as those present in more differentiated cells (retinoic acid receptors DR5 motif) (66) and binding motifs for TFs that can cooperate to drive T cell exhaustion (NFAT, BATF, IRF4) (65, 67). Collectively, our data suggest that BRD4 may regulate the expression of T cell TFs that cooperate to drive or maintain terminal T cell exhaustion in CLL. Most likely through reduced transcription at BRD4-regulated loci and/or chromatin reorganization, BET-i reduces expression of exhaustion-associated genes, curtailing IR expression and relieving TCF1 suppression by other TFs (e.g., T-BET, BATF) to promote a more progenitor-like T cell phenotype in CLL patient T cells. Future directions of this work include investigation of direct BRD4 gene regulation to better understand the dynamics of BRD4-driven/maintained T cell exhaustion in CLL and inform future use of BET inhibitors as immunomodulatory agents in the management of CLL.

Helper T cells play both pro- and antitumor roles in CLL, and expansion of various CD4^+^ T cell subsets (e.g., Th1, Th2, Th17, Tregs, follicular Th cells) have been observed in patients with CLL, supporting the intricate involvement of CD4^+^ T cells in CLL pathogenesis (68). CD4^+^ T cells from patients with CLL additionally exhibit elevated IR expression associated with advanced disease (2, 30, 68, 69). In this study, we report the broad effect of BET-i on total CD4^+^ T cells as well as classically immunosuppressive/protumor CD4^+^ T cells in the leukemia microenvironment. OPN5 reduced IR expression and improved proliferation of total CD4^+^ T cells in human cell cultures and enhanced production of Th1- and Th2-associated cytokines in Eμ-TCL1 mice. OPN5 treatment also altered key TF expression in CD4^+^ T cells, begetting a stem-like phenotype. Both Tregs and follicular Th cells were reduced with OPN5 treatment (Supplemental Figure 4F, Supplemental Figure 18G, and Supplemental Figure 20), indicating a shift toward more inflammatory CD4^+^ T cell populations. Thus, BET-i appears to influence the maintenance of polyfunctional progenitor CD4^+^ and CD8^+^ T cells in CLL, and it will be important to tease out specific effects of BET-i on Th subsets.

Despite impressive single-agent activity in CLL (12), BET-i may be most effective in combinatory treatment regimens. Preclinical studies have shown that BET-i synergizes with other small molecule inhibitors, such as venetoclax (15) and IBR (16), in B cell non-Hodgkin lymphoma (NHL). We and others (12, 70) demonstrate that BET inhibitors could serve as immunomodulators to help potentiate CLL patient responses to targeted inhibitors. Strategies combining epigenetic-targeted drugs with immunotherapies have strong rationale for clinical development, and some have already demonstrated preclinical activity in cancers (71, 72), including CLL (70, 73). In conjunction with recent studies (70, 74), our findings indicate that BET-i could be employed in combination with checkpoint blockade, CAR T cells, or bispecific antibodies (75, 76) to promote tumor clearance by enriching the TME with progenitor T cells expressing fewer exhaustion markers and sensitizing leukemic cells to CD8^+^ T cell–mediated killing.

Although we show that BET-i potently downregulates numerous immune checkpoint mechanisms in CLL, it can also evoke antiinflammatory effects in contexts of acute GVHD (47), myelofibrosis (77), cardiac dysfunction, and viral infection (78). BET inhibitors owe this dual potential to BD binding preferences (79). OPN5 exhibits modest preference for BD1 (anticancer) over BD2 (antiinflammatory) (12), suggesting that this therapy has lower potential to elicit autoimmune responses in patients with cancer. Moreover, both OPN5 and OPN-2853 exhibit favorable pharmacokinetic profiles and tolerability in mice with high peak plasma compound concentrations and short terminal half-life (12, 47).

We demonstrate that the pan-BET inhibitor OPN-51107 reshapes the leukemia microenvironment with marked reversal of immunosuppressive mechanisms inherent to CLL, thereby enhancing T cell function and promoting the maintenance of progenitor T cells, at least in part, through chromatin reorganization. This discovery corroborates similar findings in other hematological malignancies where BET proteins contribute to oncogenesis and immunosuppression (e.g., AML) (72) and supports BET-i as a useful component of treatment strategies in CLL to yield lasting anticancer control.

## Methods

### Sex as a biological variable

CLL occurs in males and females at a 1.9:1 ratio (80). For experiments utilizing human samples, samples were used from both male (*n* = 25) and female (*n* = 10) patients with CLL. For experiments with transgenic Eμ-TCL1 mice, both male and female mice were used. Since studies have shown that male Eμ-TCL1 mice tend to develop disease somewhat later and live longer on average than females (81), we utilized mice based on comparable disease burden. For Eμ-TCL1 AT studies, only female WT C57BL/6J (WT B6) mice were used. It is expected that the findings are relevant in male mice.

### Inhibitors/drugs

Plexxikon Inc. provided the pan-BET inhibitor, OPN-51107 (OPN5). Our collaboration has since been adopted by Opna Bio LLC, which acquired OPN-51107 and OPN-2853, a more potent analog, in March 2022. IBR was purchased from Cayman Chemicals. For in vivo experiments, OPN5 (12) was dissolved in 10% N-methyl-2-pyrrolidone plus diluent (40% PEG400, 5% TPGS, 5% Poloxamer 407, and 50% water) provided by Opna Bio LLC and administered via oral gavage. For ex vivo experiments, inhibitors were dissolved in DMSO (MilliporeSigma).

### Murine studies

Eμ-TCL1 transgenic mice (82) were provided by Carlo M. Croce (The Ohio State University). For AT studies, WT B6 mice (approximately 7 weeks old, The Jackson Laboratory) were engrafted via tail vein injection with 1 × 10^7^ spleen-derived lymphocytes from a moribund Eμ-TCL1 mouse as previously described (12, 17). Once leukemic cells were detectable in peripheral blood (≥10% CD45^+^CD19^+^CD5^+^ PBLs), recipient mice were randomized to receive either OPN5 (20 mg/kg, p.o.) or VEH daily for up to 4 weeks. For transgenic murine studies, Eμ-TCL1 mice with evident leukemia (> 30% CD45^+^CD19^+^CD5^+^ PBLs) were randomized to receive either OPN5 (20 mg/kg, PO) or VEH for up to 10 days. At study end, harvested spleens were homogenized into a single-cell suspension by passing through a 70 μm filter; then, RBCs were lysed (RBC Lysis Buffer; BioLegend) prior to T cell isolation or staining for flow cytometry analysis. Murine splenic T cells were isolated using EasySep Mouse T cell Isolation Kit (Stemcell Technologies) and maintained at 37°C in RPMI-1640 with 2 mM L-glutamine (MilliporeSigma) supplemented with 10% heat-inactivated FBS (hi-FBS, Avantor), 100 U/mL penicillin/100 μg/mL streptomycin (P/S, MilliporeSigma), 55 μM 2-mercaptoethanol (Thermo Fisher Scientific), 100 μM MEM nonessential amino acid solution (Lonza), 1 mM sodium pyruvate (Lonza) and 10 mM HEPES buffer (MilliporeSigma).

### Primary human samples

Characteristics of samples from patients with CLL used are tabulated in Supplemental Table 1. PBLs from healthy age-matched donors (median age 62 years) were obtained from the UNMC Elutriation Core. PBMCs were isolated from CLL patient peripheral blood using Lymphoprep (Stemcell Technologies) density gradient centrifugation (1,200*g* for 10 minutes at room temperature) following manufacturer protocols. CLL B cells were isolated from PBMCs using the EasySep Human B cell Enrichment Kit without CD43 Depletion (Stemcell Technologies). T cells were purified from CLL patient PBMCs or healthy donor PBLs using the EasySep Human T cell Isolation Kit (Stemcell Technologies). Human cells were maintained at 37°C in RPMI-1640 with 2 mM L-glutamine supplemented with 10% hi-FBS and P/S.

### Murine T cell functional assays

#### T cell proliferation.

Splenic T cells labeled with 2 μM Cell Trace Violet (Invitrogen) were stimulated with 10 μg/mL plate-bound anti-CD3 (clone 145-2C11, BioLegend) and 1 μg/mL anti-CD28 (clone 37.51, BioLegend) for 72 hours. Proliferation index was calculated as the average number of divisions responding cells underwent.

#### Effector cytokine production.

Splenic T cells were stained with 5 μg/mL anti-CD107a and stimulated for 6 hours with 1× PMA/ionomycin (BioLegend), with 1× Brefeldin-A (BioLegend) added for the final 5 hours. 

### Human T cell functional assays

For coculture experiments, a mixed lymphocyte system (3) with minor modifications to optimize induction of exhaustion phenotypes was employed as detailed below.

#### Exhaustion marker and T cell TF expression.

CLL patient–derived PBMCs or T cells, healthy donor T cells cultured alone, or healthy donor T cells cocultured with CLL B cells at a 2:1 (B cell/T cell [B:T]) ratio were stimulated with 10 μg/mL plate-bound anti-CD3 (clone UCHT1, BioLegend) and 5 μg/mL anti-CD28 (clone CD28.2, BioLegend), and they were treated with indicated inhibitors for 48 hours.

#### Time course cocultures.

Healthy donor T cells cultured alone or healthy donor T cells cocultured with CLL B cells at a 2:1 (B:T) ratio were stimulated with anti-CD3/anti-CD28 (BioLegend) for 2, 4, or 7 days. Cocultures were either treated with the indicated inhibitors continuously, or CLL B cells were pretreated for 48 hours, followed by inhibitor wash off of prior coculture. Cultures were split 1:1 following day 4 collection.

#### T cell proliferation.

Healthy donor T cells labeled with 2 μM Cell Trace CFSE (Invitrogen) were cultured alone or cocultured with CLL B cells at a 5:1 (B:T) ratio, stimulated with anti-CD3/anti-CD28 (BioLegend) and 250 ng/mL CXCL12 (Sino Biological), and treated with the indicated inhibitors for 96 hours.

#### Effector cytokine production.

CLL patient–derived T cells, or healthy donor T cells cultured alone or cocultured with CLL B cells at a 2:1 (B:T) ratio in the presence of 3.2 μM CpG 2006 oligonucleotides (CpG ODN; Integrated DNA Technologies), were treated with the indicated inhibitors for 48 hours. Cells were then stained with anti-CD107a and stimulated with 1× PMA/ionomycin in the presence of 1× Brefeldin-A as described above.

### Human CD8+ T cell cytotoxicity assay

CD8^+^ T cells were isolated from CLL patient PBMCs using EasySep Human CD8^+^ T cell Isolation Kit (Stemcell Technologies) and treated with the indicated inhibitors for 48 hours. The CLL cell line, MEC-1 was labeled with 1 μM CFSE (Invitrogen) before being coated with 2 μg/mL anti-CD3 (BioLegend) for 45 minutes at 37°C prior to coculture. In total, 30,000 viable CFSE-labeled MEC-1 cells were cocultured with 30,000 viable pretreated CD8^+^ T cells. Cells were maintained in RPMI 10% FBS containing 5 ng/mL 1L-15 and 20 IU/mL IL-2 (Stemcell Technologies) during pretreatment and coculture as previously described (44). Cocultures were incubated for 18 hours at 37°C before being stained with Zombie NIR viability dye (BioLegend) for 20 minutes at 4°C. The number of dead (Zombie NIR positive) MEC-1 cells per 100 live (Zombie NIR negative) CD8^+^ T cells was determined via flow cytometry analysis.

### Flow cytometry

A list of fluorochrome-labeled antibodies and gating strategies is provided in Supplemental Methods. Data acquisition was performed on a LSRII (BD Biosciences), LSRFortessa X-50 (BD Biosciences), or NovoCyte 2060R (Agilent) cytometer. Data were analyzed using NovoExpress v1.3.0 (Agilent) or Kaluza v2.1 (Beckman Coulter). Unsupervised clustering was conducted using the FlowSOM algorithm (27) on the Cytobank platform (Beckman Coulter) or OMIQ. Data were analyzed using hierarchical consensus clustering, equal sampling of events, and 8–10 metaclusters with 81–100 clusters, and they were run for 10 FlowSOM iterations.

### Gene expression profiling

The PanCancer IO360 panel (NanoString Technologies) was used to interrogate gene expression in splenic cells and PBMCs from leukemic mice treated for 21 days with OPN5 or VEH (*n* = 3/ group). Total RNA was isolated using RNeasy Mini Kit (Qiagen) protocol and run on the nCounter platform (NanoString Technologies) at the UNMC Genomics Core per manufacturer instructions. nSolver 4.0 Advanced Analysis module (NanoString Technologies) with default settings was used to derive DEGs. Relative cell-type scores were generated using log_2_ expression levels of cell-type–specific mRNAs and normalized to the abundance of tumor infiltrating leukocytes (average of B cell, T cell, CD45, macrophage, and cytotoxic cell scores) (83). Log_2_ fold change and *P* value data were subject to Ingenuity Pathway Analysis (IPA) (Qiagen; accessed April 2022; ref. 84), and results were obtained as modulated canonical pathways with significance of *P* < 0.001. DEGs (*P* < 0.05) were imported to Molecular Signatures Database (MSigDB) v7.5.1 (85) for GSEA to determine the top BioCarta, KEGG, PID, WikiPathways, Reactome, and GO Biological Processes gene sets modulated with OPN5 treatment.

### ATAC-Seq

CLL patient PBMCs were treated with 0.5 μM OPN5 or VEH for 16 hours in the presence of 10 μg/mL anti-CD3 (clone UCHT1, BioLegend) and 5 μg/mL anti-CD28 (clone CD28.2, BioLegend). Live CD8^+^ T cells were then isolated via FACS. Approximately 100,000 CD8^+^ T cells were subjected to nuclei isolation, tagmentation, DNA purification, and indexed amplification using the Active Motif ATAC-Seq Kit according to manufacturer instructions. Reactions were cleaned up with AMPure XP beads (Beckman Coulter). Libraries were quantified with a Qubit fluorometer (Thermo Fisher Scientific), and fragment analysis was performed with Bioanalyzer (Agilent). Libraries underwent paired-end sequencing (2 × 50 bp, 100 cycles) on an Illumina NovaSeq 6000 SP sequencer at the UNMC Genomics Core.

### ATAC-Seq analysis

Raw ATAC-Seq data were processed using the Encyclopedia of DNA Elements (ENCODE) ATAC-Seq processing pipeline (release v2.3.3) (86). This comprehensive workflow includes quality control checks using cutadapt (v1.9.1) (87), alignment of sequencing reads to the human reference genome (GRCh38/hg38) using Bowtie2 (v2.2.6) (88), removal of duplicates with Picard (v1.126) (89), and SAMtools (v1.7) (90) as well as the BEDTools suite (v2.26) (91) for file format conversion, followed by peak calling using MACS2 (v2.1.0) (92). Differential peak analysis was performed using MAnorm (v1.3.0) (93) ropController to merge peaks called in individual samples and to perform statistical assessment of differences under a *P* value threshold < 0.05 and a log_2_ fold change of at least 1. Differential peaks were called in individual patients, and overlaps between individuals were identified using BEDTools. Signal at differential peaks was visualized by Deeptools for heatmap creation (94). Annotation of identified ATAC-Seq peaks was conducted using Hypergeometric Optimization of Motif EnRichment (HOMER) (v4.11) (90). Comparison of the global changes in chromatin accessibility at the gene level was performed using gene set variation analysis (GSVA) (v1.48.3) (95) of the ATAC-Seq signal within promoters, defined as 250 bp upstream of genes. Scripts for postprocessing and figure creation can be found at https://github.com/jrowleylab/ATAC_Seq_TCell_Downstream_Analysis (commit ID: 57df234) (96).

### Statistics

All statistical tests were performed using Prism v9.5.1 (Graphpad Software Inc.) and are detailed in Supplemental Methods. The significance of the differences between mean values of 2 groups (inhibitor versus vehicle) was assessed using unpaired Mann-Whitney *U* test. One-way ANOVA with Tukey’s multiple-comparison test was performed for comparisons of more than 2 groups (multiple inhibitor concentrations). Two-way ANOVA with Tukey’s multiple-comparison test was performed for comparisons across multiple parameters (time and treatment). *P* values less than 0.05 were considered statistically significant.

### Study approval

All animal experiments were approved by the UNMC IACUC. All human samples were obtained following informed consent under an UNMC IRB–approved protocol for experimental use in accordance with the Declaration of Helsinki.

### Data availability

NanoString gene expression data are available at GEO (accession no. GSE262027). ATAC-Seq data have been deposited to dbGaP (accession no. phs003613). Individual patient ATAC-Seq data files are under controlled access and data use limitations, per patient confidentiality agreement. Summary data are available without restriction. Values for all data points in figures are reported in the Supporting Data Values file.

## Author contributions

ALS, SAS, APE, ES, NW, DYM, and DE performed experiments and analyzed the data; TER and MJR analyzed ATAC-Seq data; ALS and DE formulated the conceptualization and experimental design; ALS, TER, MJR, CRD, EM, ARM, PWD, and DE contributed to the interpretation of the results; CRD, AK, MAL, RGB, and JMV accrued patients, provided critical translational insight that helped shape the discussion, and reviewed the manuscript; BP and GB provided the study drug and recommendations for its use; ALS and DE wrote the original manuscript draft and obtained input from all other authors who reviewed and edited the manuscript. DE managed and supervised all study aspects and obtained funding. All authors have read and agreed to the published version of the manuscript.

## Supplementary Material

Supplemental data

Supporting data values

## Version 1 05/22/2024

Electronic publication
