# Supplementary material for: BET inhibition reforms the immune microenvironment and alleviates T cell dysfunction in chronic lymphocytic leukemia
Source: JCI Insight. 2024 May 22;9(10):e177054. doi: 10.1172/jci.insight.177054 (PMC11141939; doi:10.1172/jci.insight.177054)
Supplement: Supplemental data [file jciinsight-9-177054-s274.pdf]

SUPPLEMENTAL INFORMATION

SUPPLEMENTAL DATA

I. Supplemental Figures

Supplemental Figure 1

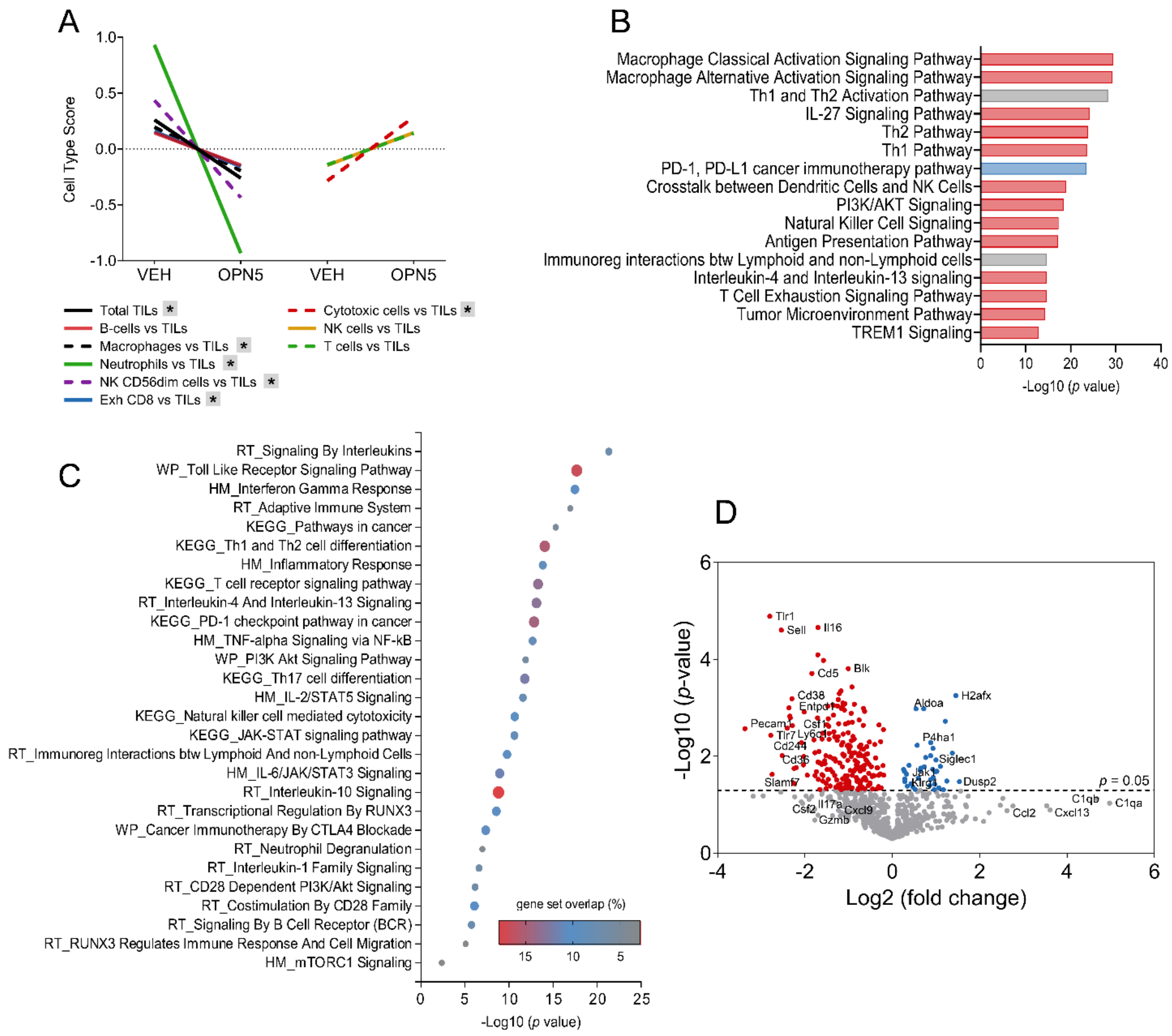

Supplemental Figure 1. NanoString (PancCancer iO360) profiling of PBMCs following in vivo BET inhibitor treatment.

Gene expression profiling of PBMCs from adoptive transfer Eμ-TCL1 mice treated with OPN-51107 (OPN5) or vehicle equivalent (VEH) for 21 days (n=3/treatment group). **(A)** NanoString cell type scoring indicating differences in immune cell populations following treatment. TILs: tumor-infiltrating lymphocytes; NK cells: natural killer cells; Exh: exhausted. Unpaired, two-tailed Mann-Whitney U tests were used to determine significant difference between VEH and OPN5 groups for each cell type. *$p < 0.05$. **(B)** Significantly modulated canonical pathways identified by IPA following BET inhibition with OPN5. The direction of z-score is indicated by bar coloring: activated (blue), inhibited (red), no activity pattern available (gray). **(C)** MSigDB analysis of genes significantly modulated by OPN5 treatment ($p < 0.05$). Gene set overlap is defined as (the number of significantly modulated genes that fall in a pathway gene set/ the total number of genes in that gene set) x 100. **(D)** Volcano plot of differentially expressed genes (OPN5 vs. VEH).

Supplemental Figure 2

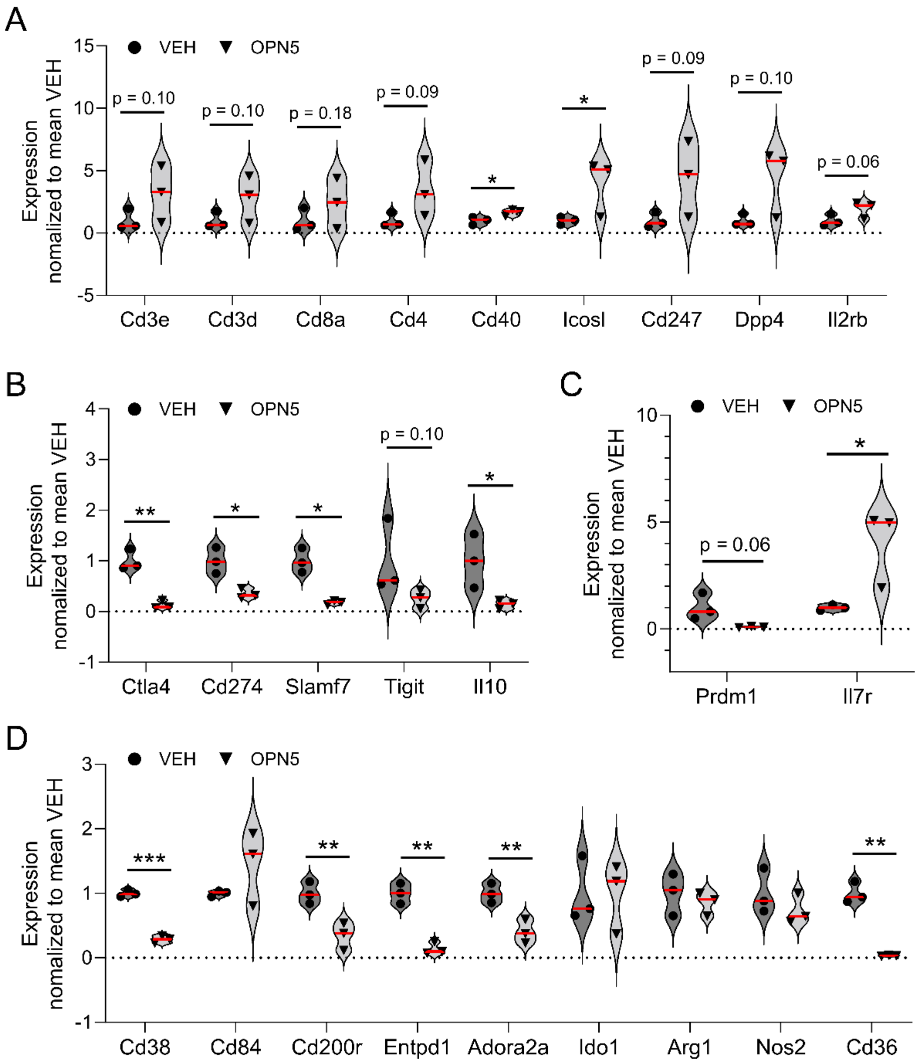

Supplemental Figure 2. Differentially expressed splenic genes following in vivo BET inhibitor treatment.

Select genes found to be differentially expressed in targeted gene expression analysis (NanoString PanCancer iO360 panel) of splenic tissue from Eμ-TCL1 adoptive transfer mice treated with OPN-51107 (OPN5) or vehicle equivalent (VEH) for 21 days (n=3/treatment group). **(A)** Genes involved in T-cell activation. **(B)** Genes involved in T-cell inhibition. **(C)** Genes involved in T-cell differentiation. **(D)** Genes pertaining to TME myeloid cell function. Expression values are normalized to the average of VEH-treated mice. Unpaired, two-tailed Mann-Whitney U tests were used to determine significant difference between VEH and OPN5 groups for each gene. $^*p < 0.05$, $^{**}p < 0.01$ $^{***}p < 0.001$.

Supplemental Figure 3

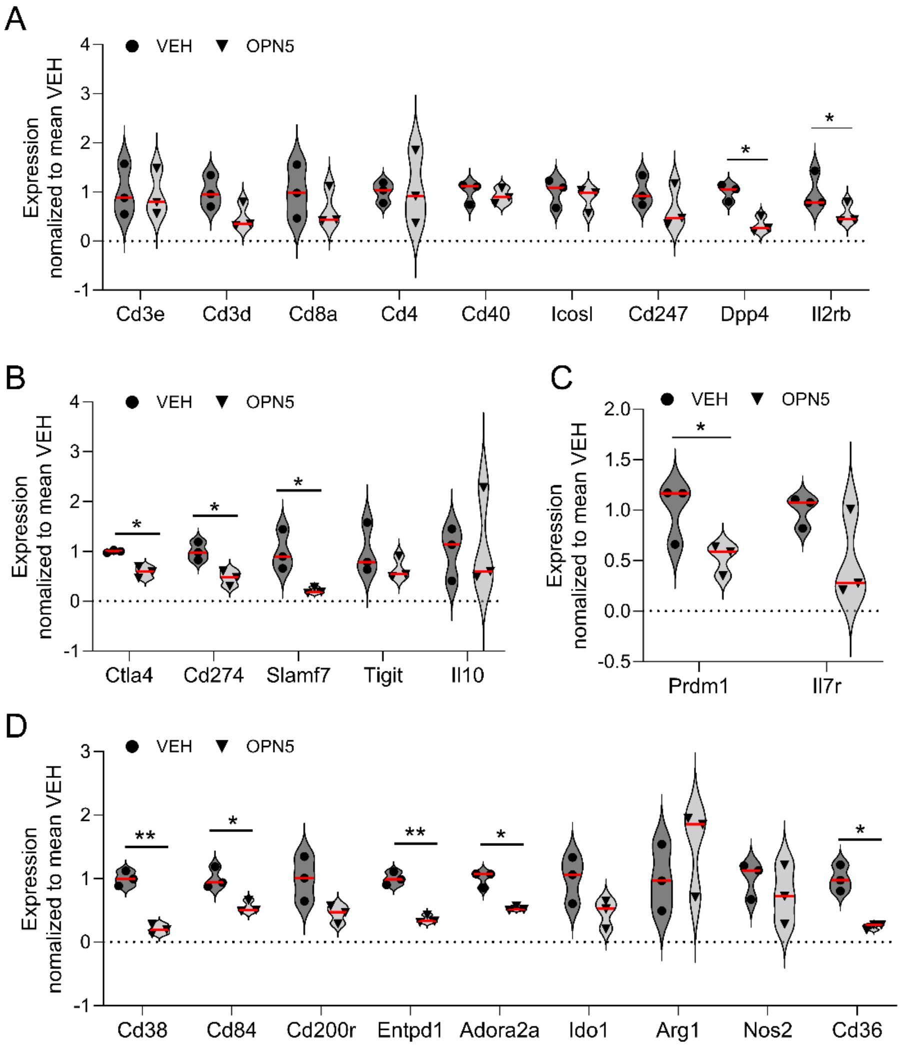

Supplemental Figure 3. Differentially expressed PBMC genes following in vivo BET inhibitor treatment.
Select genes found to be differentially expressed in targeted gene expression analysis (NanoString PanCancer iO360 panel) of PBMCs from Eμ-TCL1 adoptive transfer mice treated with OPN-51107 (OPN5) or vehicle equivalent (VEH) for 21 days (n=3/treatment group). **(A)** Genes involved in T-cell activation. **(B)** Genes involved in T-cell inhibition. **(C)** Genes involved in T-cell differentiation. **(D)** Genes pertaining to TME myeloid cell function. Expression values are normalized to the average of VEH-treated mice. Unpaired, two-tailed Mann-Whitney U tests were used to determine significant difference between VEH and OPN5 groups for each gene. *$p < 0.05$, **$p < 0.01$.

Supplemental Figure 4

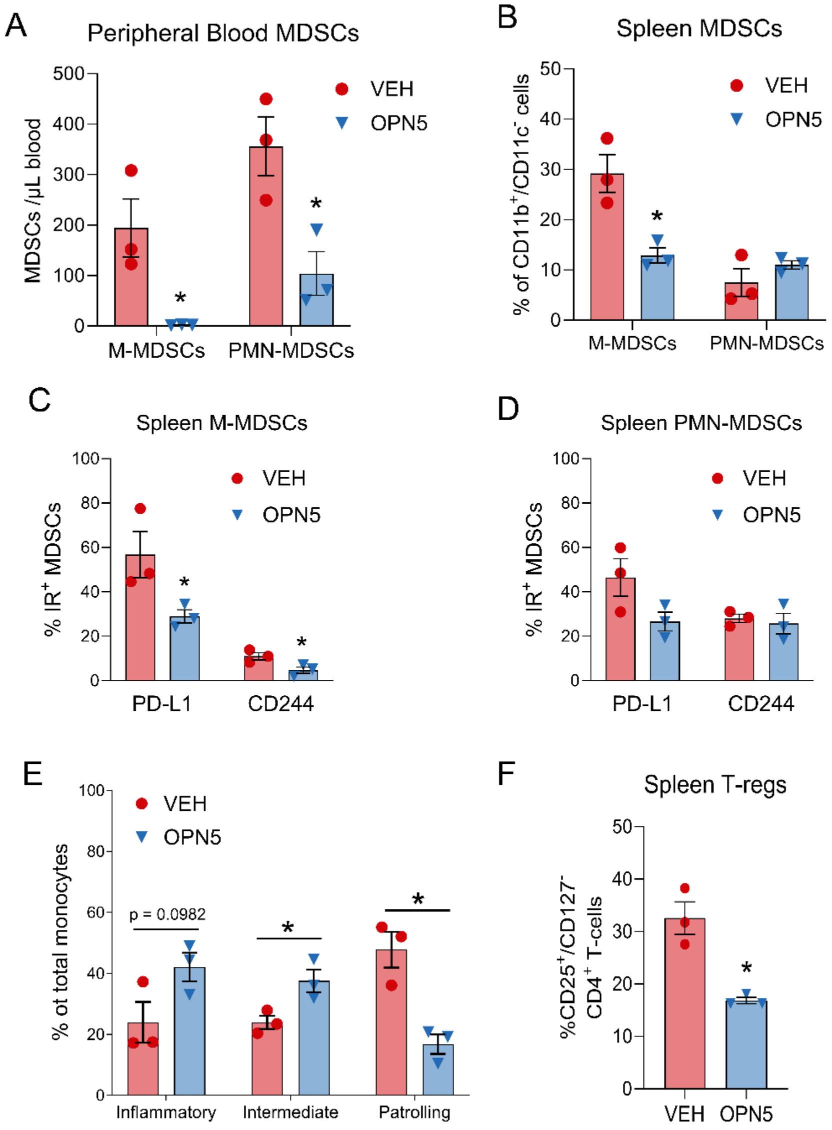

Supplemental Figure 4. Immuno-suppressive cell populations in the CLL TME.
Eµ-TCL1 adoptive transfer mice treated with OPN-51107 (OPN5) or vehicle (VEH) equivalent for 21 days (n=3/treatment group). **(A)** Monocytic MDSCs (M-MDSCs) gated as B220$^-$/CD3$^-$/CD11b$^+$/Ly6C$^+$/Ly6G$^-$ cells in the peripheral blood. **(B)** Granulocytic MDSCs (PMN-MDSCs) gated as B220$^-$/CD3$^-$/CD11b$^+$/Ly6C^{lo}/Ly6G$^+$ cells in the peripheral blood. **(C)** % M-MDSCs in the spleen gated as CD19$^-$/CD3$^-$/CD11b$^+$/Ly6C$^+$/Ly6G$^-$ cells. **(D)** % PMN-MDSCs in the spleen gated as CD19$^-$/CD3$^-$/CD11b$^+$/Ly6C^{lo}/Ly6G$^+$ cells. **(E)** Subsets of splenic monocytes gated as CD19$^-$/CD3$^-$/CD11b$^+$/LyG$^-$ then subdivided by Ly6C and CD43 expression: inflammatory (Ly6C^{hi}/CD43^{lo}), intermediate (Ly6C^{med}/CD43^{med}), and patrolling (Ly6C^{lo}/CD43^{hi}). **(F)** Splenic T-regs gated as CD3$^+$/CD4$^+$/CD25$^+$/CD127$^-$ cells. Unpaired, two-tailed Mann-Whitney U tests were used to determine significant difference between VEH and OPN5 groups. *$p < 0.05$.

Supplemental Figure 5

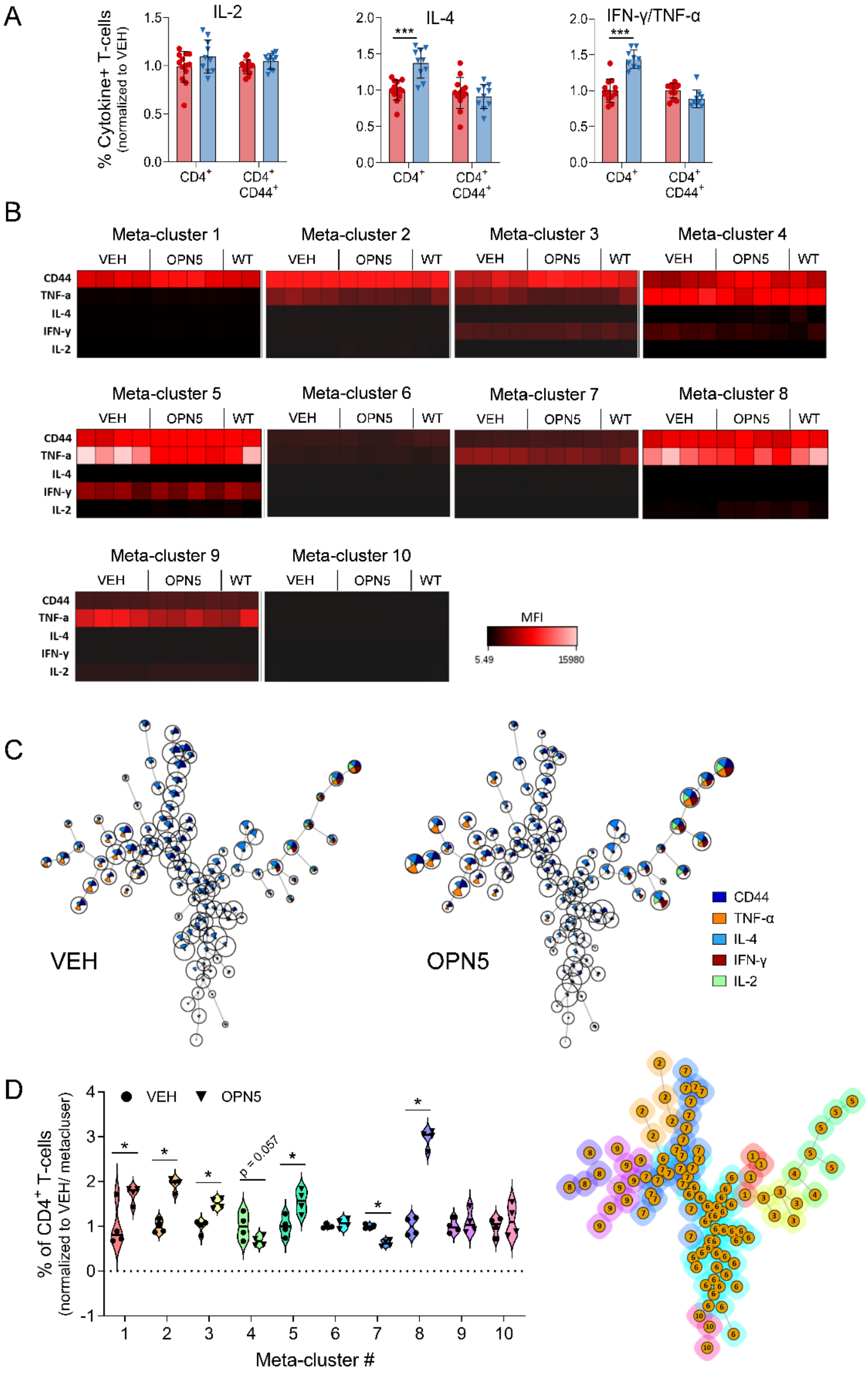

Supplemental Figure 5. Cytokine production in splenic CD4$^+$ T-cells.

(A) Splenic T-cells from vehicle (VEH; n=12) and OPN-51107 (OPN5; n=10) treated mice stimulated ex vivo for 6 h with PMA/ionomycin, then evaluated by flow cytometry for percentages of T-cells expressing intracellular cytokines. **(B-D)** FlowSOM clustering of splenic CD4$^+$ T-cells from a subset of adoptive transfer Eμ-TCL1 mice treated with OPN5 (n=4) or VEH (n=4) based on expression of CD44, TNF-α, IL-4, IFN-γ, and IL-2. Age-matched wild type (WT) mice were included as internal experimental controls (n=2). **(B)** Heatmaps showing relative expression of each marker (rows), for each evaluated mouse (columns), for each identified meta-cluster. **(C)** Representative clustering star plots from VEH- and OPN5- treated mice. **(D)** Fold change in the percentage of CD4$^+$ T-cells found in each FlowSOM-identified meta-cluster. Data is represented as mean ± standard error of the mean. MFI: median fluorescent intensity. Unpaired, two-tailed Mann-Whitney U tests were used to determine significant differences between VEH and OPN5 groups for each meta-cluster. *$p < 0.05$, ***$p < 0.001$.

Supplemental Figure 6

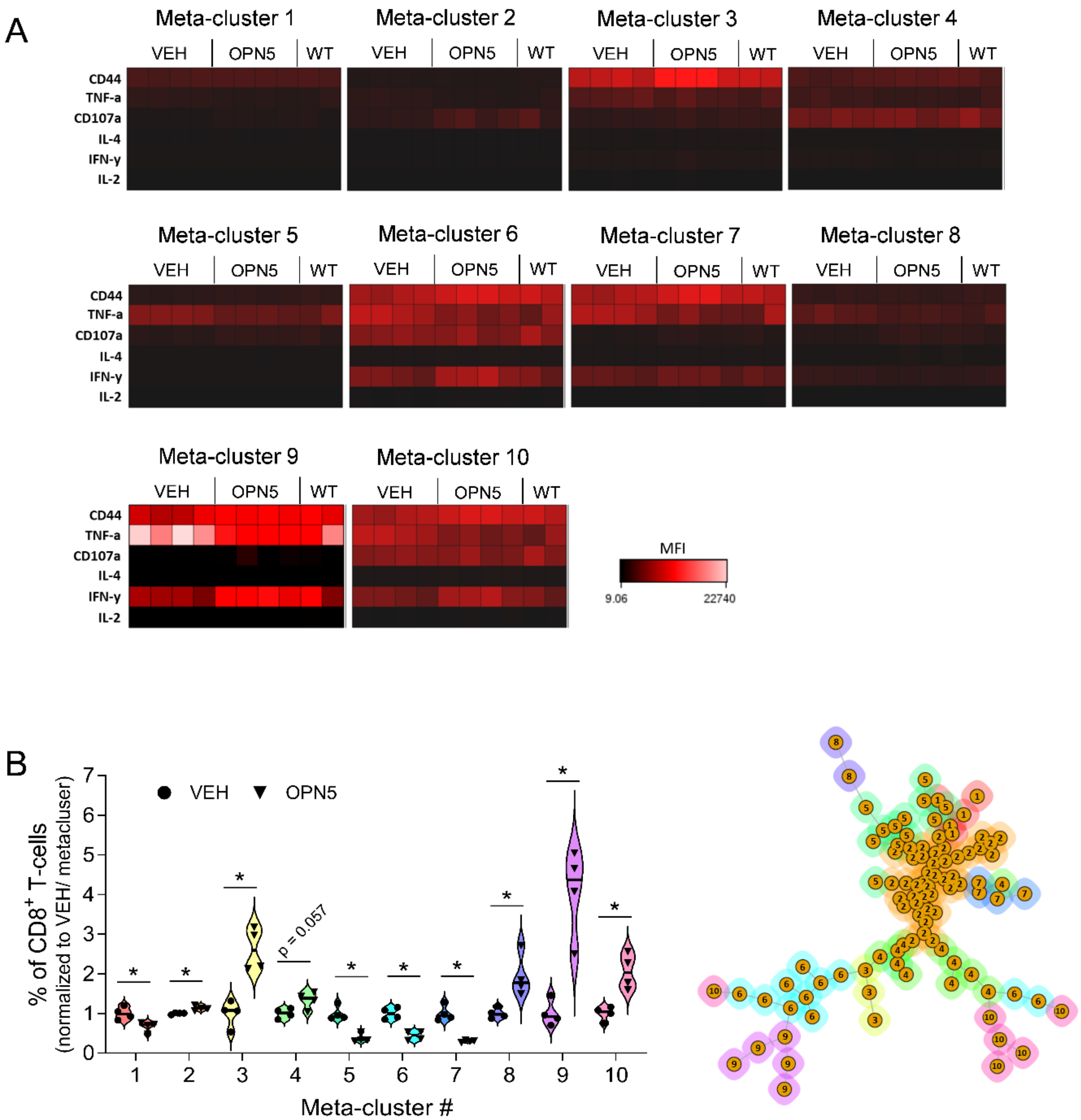

Supplemental Figure 6. FlowSOM clustering of splenic CD8^{+} T-cell cytokines. Clustering of flow cytometry data from a subset of adoptive transfer Eμ-TCL1 mice treated with OPN-51107 (OPN5, n=4) or vehicle equivalent (VEH, n=4) based on expression of CD44, CD107a, TNF-α, IL-4, IFN-γ, and IL-2. Age-matched wild type (WT) mice were included as internal experimental controls (n=2). **(A)** Heatmaps showing relative expression of each marker (rows), for each evaluated mouse (columns), for each identified meta-cluster **(B)** Fold change in the percentage of CD8^{+} T-cells found in each FlowSOM-identified meta-cluster. Data is represented as mean ± standard error of the mean. MFI: median fluorescent intensity. Unpaired, two-tailed Mann-Whitney U tests were used to determine significant difference between VEH and OPN5 groups for each meta-cluster. *$p < 0.05$.

Supplemental Figure 7

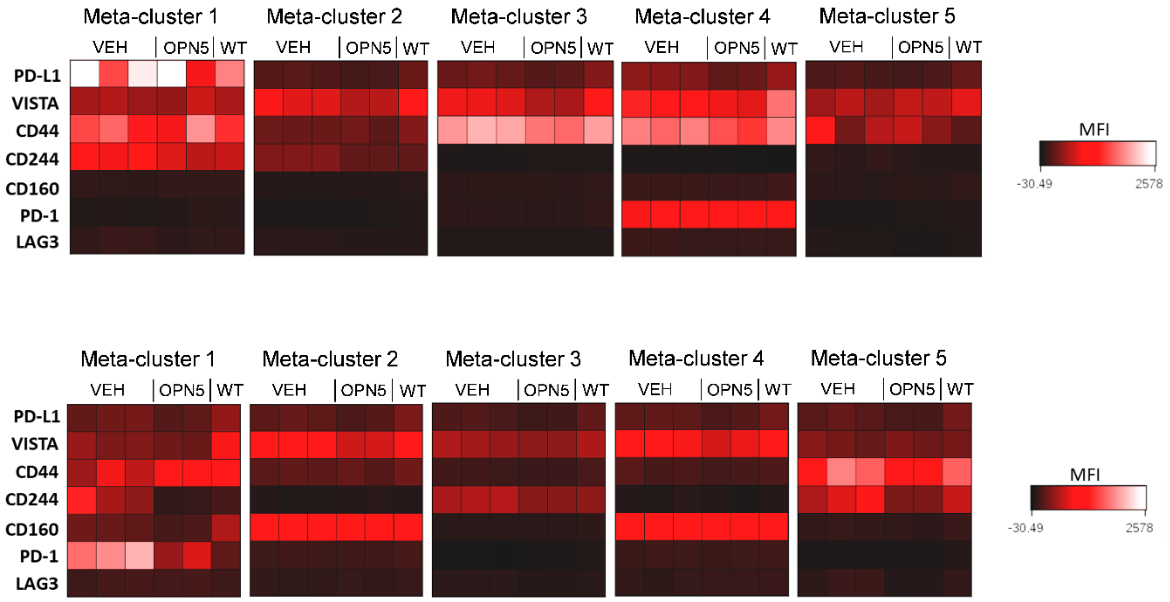

Supplemental Figure 7. FlowSOM clustering of total splenic T-cells based on inhibitory receptor expression.

Detected meta-clusters for total splenic T-cells from vehicle (VEH; n=3) and OPN-51107 (OPN5; n=2) treated adoptive transfer Eμ-TCL1 mice. Age-matched wild type (WT) mice were included as internal experimental controls (n=2). Heatmaps show the relative expression of each marker (rows), for each evaluated mouse (columns). MFI: median fluorescent intensity.

Supplemental Figure 8

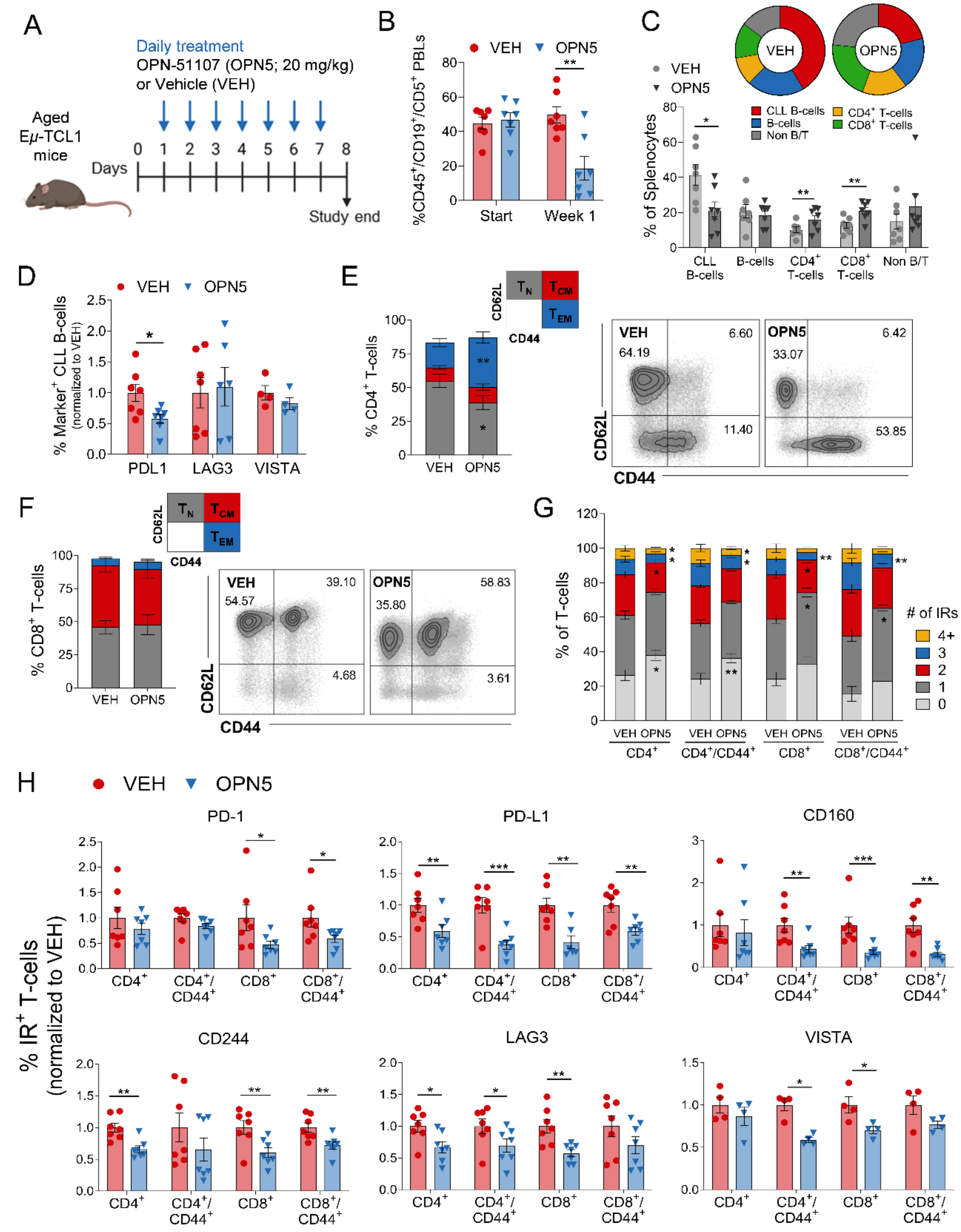

Supplemental Figure 8. BET inhibition with OPN-51107 exhibits positive immuno-modulatory potential in an indolent murine model of CLL.

(A) Diseased Eμ-TCL1 mice (average age = 11.6 ± 1.6 mo) were randomly assigned to treatment with OPN-51107 (OPN5; n=3 male, n=4 female) or vehicle equivalent (VEH; n=3 male, n=4 female) via oral gavage daily for 1 week. **(B)** Flow cytometry evaluation of disease burden in murine peripheral blood. **(C)** Percentages of abundant cell types found in the spleens of VEH- and OPN5-treated mice. CLL B-cells were gated as $CD45^+/CD19^+/CD5^+$, healthy B-cells were gated as $CD45^+/CD19^+/CD5^-$, T-cells were gated as $CD45^+/CD19^-/CD4^+$ or $CD8^+$. **(D)** Percentages of CLL B-cells expressing immune inhibitory receptors (IRs), normalized to the average of VEH-treated mice. **(E, F)** Distribution of $CD4^+$ **(E)** and $CD8^+$ **(F)** T-cells into naïve ($CD44^-/CD62L^+$, T_N), central memory ($CD44^+/CD62L^+$, T_{CM}), and effector memory ($CD44^+/CD62L^-$, T_{EM}) subsets with representative flow cytometry plots. Asterisks denote significant differences between VEH and OPN5 for each T-cell subset. **(G)** The number of evaluated immune IRs (PD-1, PD-L1, LAG3, CD160, CD244, VISTA) co-expressed on splenic T-cells. Asterisks denote significant differences between VEH and OPN5 for each # of IRs per T-cell subset. (H) Percentages of splenic T-cells expressing individual immune IRs, normalized to the average of VEH-treated mice. Summary data are represented as mean ± standard error of the mean. Unpaired, two-tailed Mann-Whitney U tests were used to determine significant difference between VEH and OPN5 groups. *$p < 0.05$, **$p < 0.01$, ***$p < 0.001$.

Supplemental Figure 9

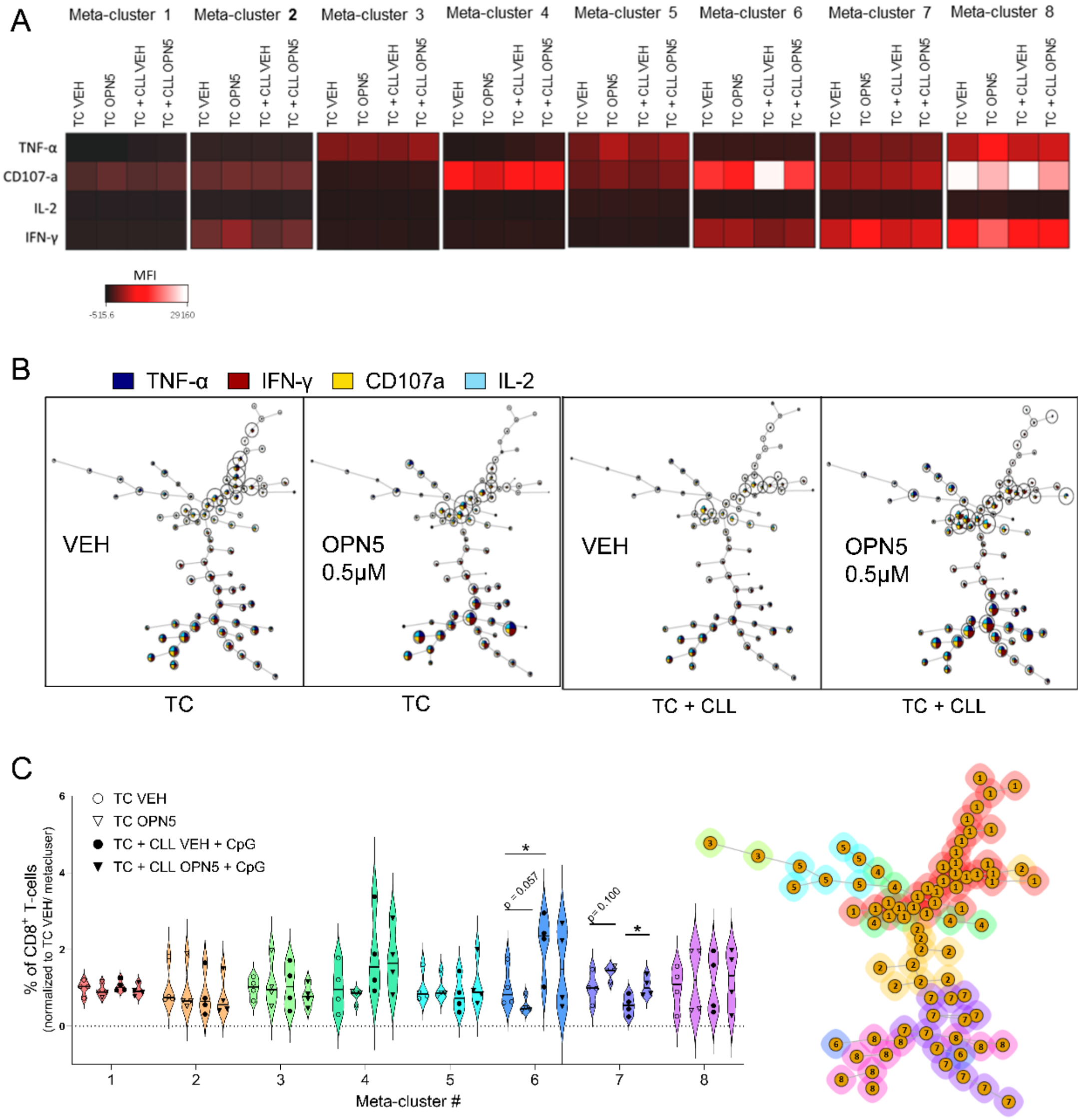

Supplemental Figure 9. FlowSOM clustering of CD8$^+$ T-cells from human T-cell/CLL co-cultures based on cytokine production.
(A) FlowSOM detected meta-clusters for PMA/ionomycin-stimulated CD8$^+$ healthy donor T-cells cultured alone (TC) or co-cultured with patient-derived CLL B-cells in the presence of CpG oligonucleotides (TC + CLL) (n=4/group). Heatmaps show the relative expression of each marker (rows), for each evaluated condition (columns). **(B)** Representative FlowSOM clustering of splenic CD8$^+$ T-cells based on the expression of TNF-α, IFN-γ, CD107a and IL-2. Relative expression is illustrated as the size of colored pie slices within each cluster. The relative abundance of each cluster is represented by cluster size. **(C)** Fold change in the percentage of T-cells found in each FlowSOM-identified meta-cluster. Data is represented as mean ± standard error of the mean. MFI: median fluorescent intensity. Significant differences were calculated using one-way ANOVA. *$p < 0.05$.

Supplemental Figure 10

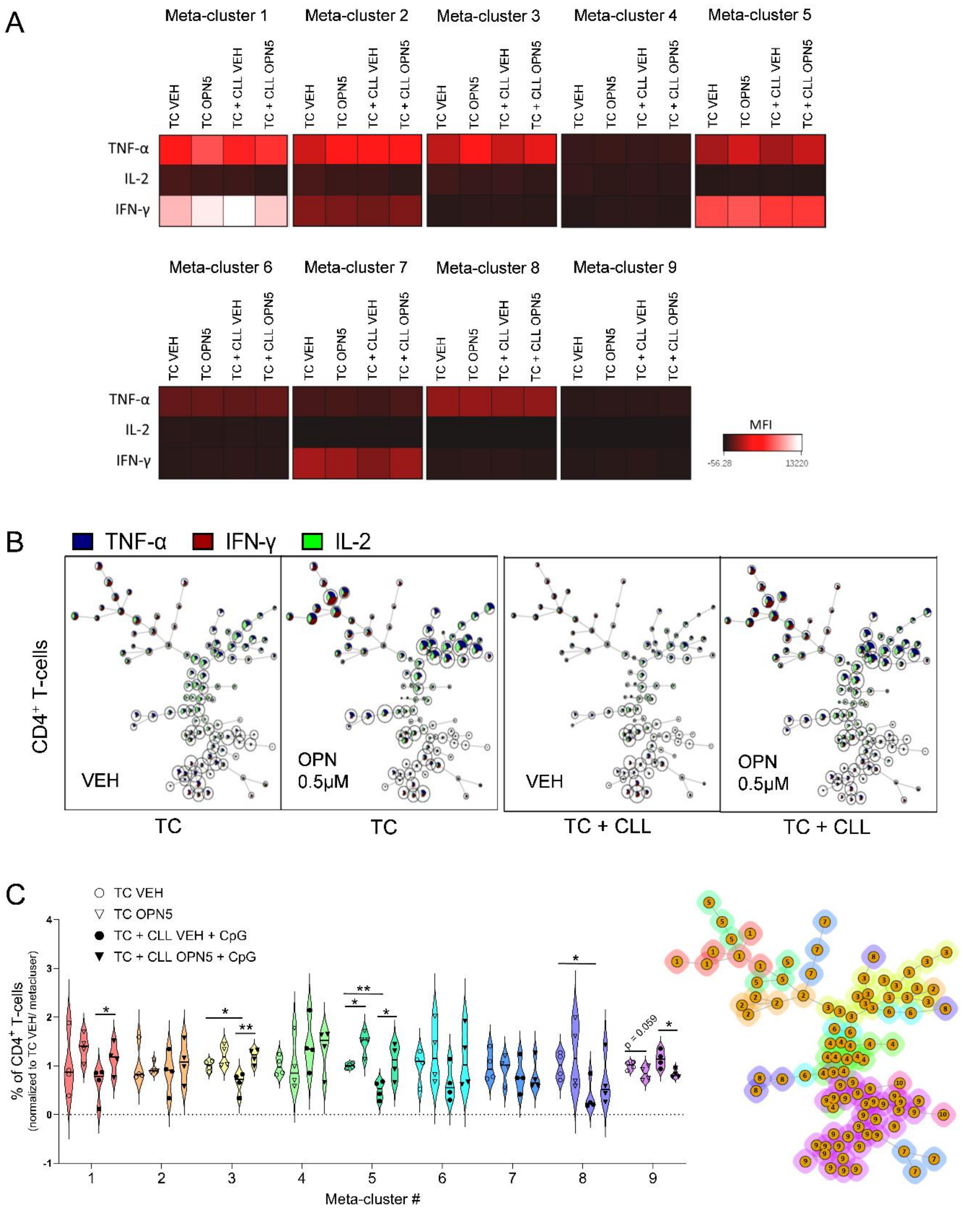

Supplemental Figure 10. FlowSOM clustering of CD4$^+$ T-cells from human T-cell/CLL co-cultures based on cytokine production.

(A) FlowSOM detected meta-clusters for PMA/ionomycin-stimulated CD4$^+$ healthy donor T-cells cultured alone (TC) or co-cultured with patient-derived CLL B-cells in the presence of CpG oligonucleotides (TC + CLL) (n=4/group). Heatmaps show the relative expression of each marker (rows), for each evaluated condition (columns). **(B)** Representative FlowSOM clustering of splenic CD4$^+$ T-cells based on the expression of TNF-α, IFN-γ, and IL-2. Relative expression is illustrated as the size of colored pie slices within each cluster. The relative abundance of each cluster is represented by cluster size. **(C)** Fold change in the percentage of T-cells found in each FlowSOM-identified meta-cluster. Data is represented as mean ± standard error of the mean. MFI: median fluorescent intensity. Significant differences were calculated using one-way ANOVA. *$p < 0.05$, **$p < 0.01$.

Supplemental Figure 11

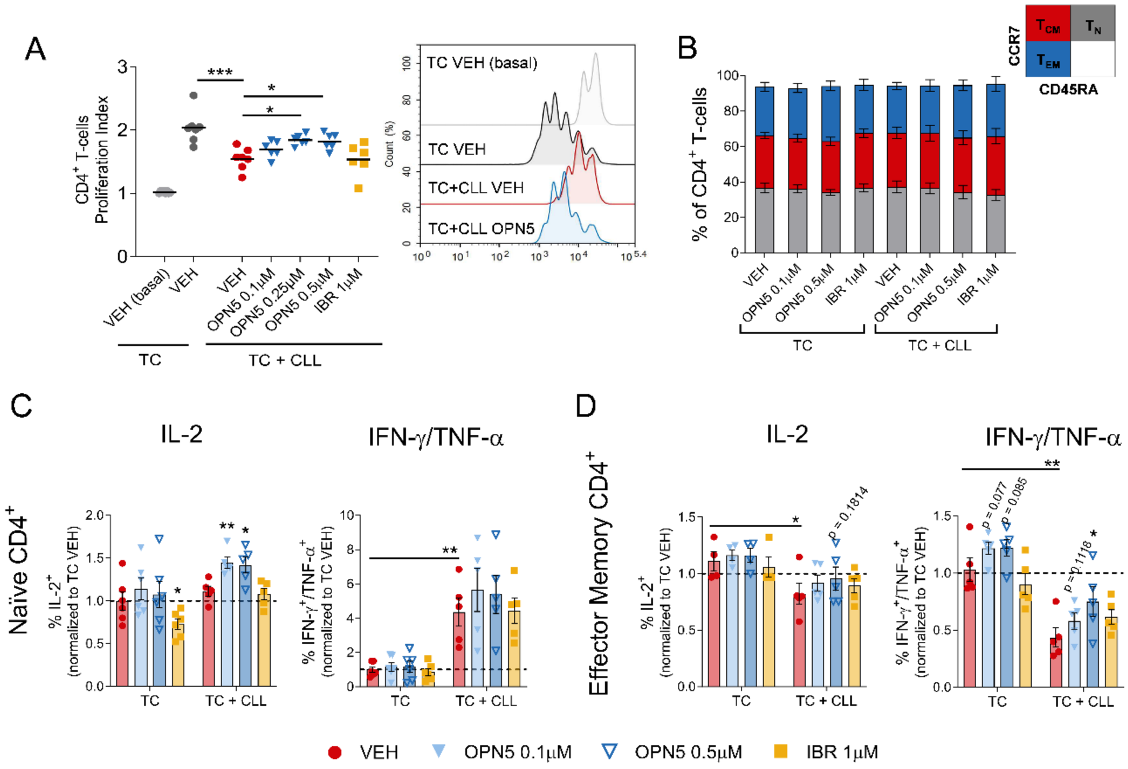

Supplemental Figure 11. BET inhibition with OPN-51107 improves the functionality of primary CLL-induced exhausted CD4$^+$ T-cells ex vivo.

(A) Flow cytometry proliferation analysis of CFSE-stained healthy donor T-cells cultured alone (TC) or co-cultured with patient-derived CLL B-cells (TC + CLL) in the presence of CXCL12, stimulated with α-CD3/α-CD28 for 96 h (n=6-8). **(B-D)** Healthy donor T-cells cultured alone or co-cultured with patient-derived CLL B-cells for 48 h in the presence of CpG oligonucleotides, stimulated with PMA/ionomycin for the final 6 h (n=4-5). **(B)** Distribution of basal CD4$^+$ T-cells into naïve (CD45RA$^+$/CCR7$^+$; T_N), central memory (CD45RA$^-$/CCR7$^+$; T_{CM}), and effector memory (CD45RA$^-$/CCR7$^-$; T_{EM}) subsets. TC + CLL = 48 h co-culture at a 2:1 (B:T) ratio. **(C, D)** Percentages of IL-2$^+$ and IFN-γ$^+$/TNF-α$^+$ T_N **(C)** and T_{EM} **(D)** CD4$^+$ T-cells upon PMA/ionomycin stimulation, normalized to VEH-treated T-cell monoculture average. Summary data is represented as mean ± standard error of the mean. Significant difference from VEH was calculated using one-way ANOVA. *p < 0.05, **p < 0.01, ***p < 0.001.

Supplemental Figure 12

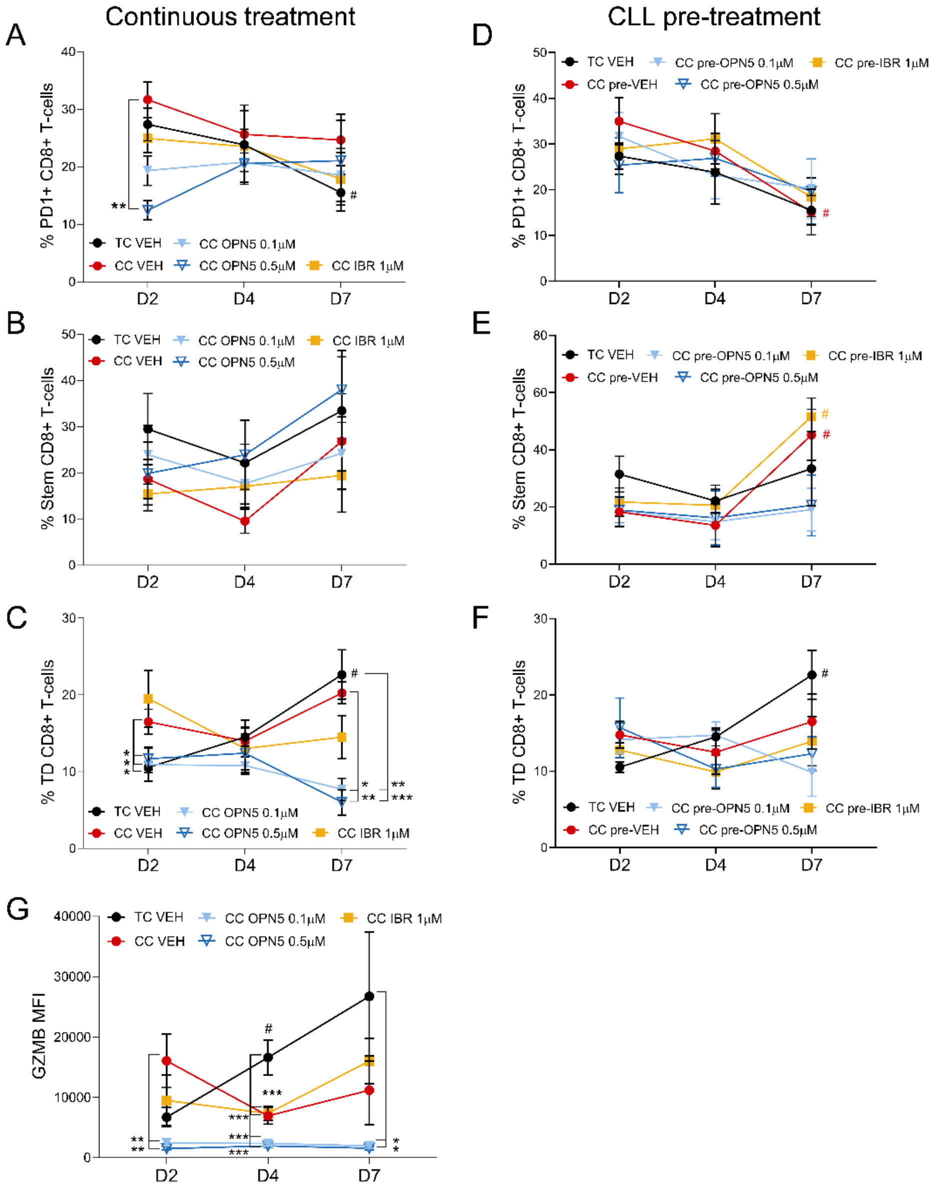

Supplemental Figure 12. Time course of primary CLL/T-cell co-culture.
Healthy donor T-cells alone (TC) or patient-derived CLL B-cells/healthy donor T-cell co-cultures (CC) were incubated for up to 7 days, stimulated with α-CD3/α-CD28 (n=5). Cultures were either treated continuously **(A-C)** with OPN-51107 (OPN5; 0.1 or 0.5 μM), ibrutinib (IBR; 1 μM), or vehicle equivalent (VEH; DMSO) or CLL B-cells were pre-treated, and drug was washed off prior co-culture **(D-F)**. **(A, D)** Percentages of PD-1^{+} CD8^{+} T-cells. **(B, E)** Percentages of stem-like (PD-1^{+}/TCF-1^{+}/Ly108^{+}) CD8^{+} T-cells. **(C, F)** Percentages of terminally differentiated (PD-1^{+}/TIM3^{+}/CD101^{+}) CD8^{+} T-cells. **(G)** GZMB MFI. Data is represented as mean ± standard error of the mean. Significant differences were calculated using two-way ANOVA. Significant difference from CC VEH within each day: *p < 0.05, **p < 0.01, ***p < 0.001. Significant difference from day 2 for the indicated color treatment group: #p < 0.05. D: day.

Supplemental Figure 13

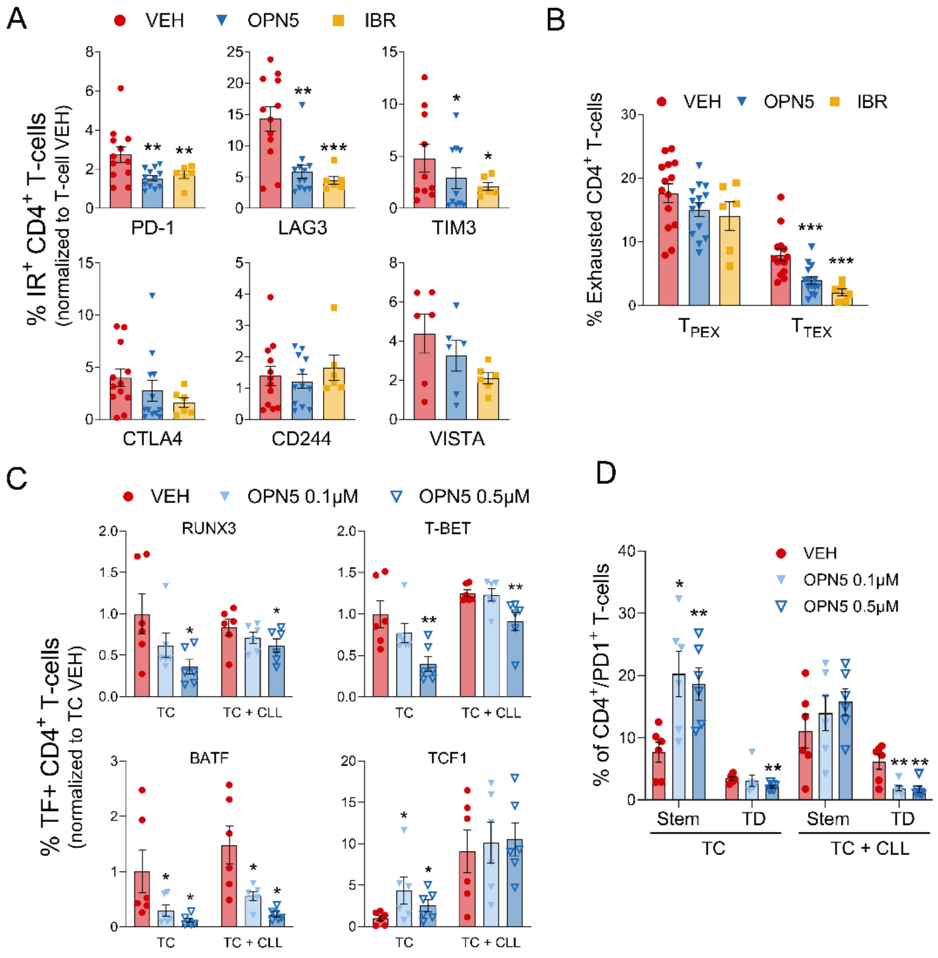

Supplemental Figure 13. Inhibitory receptor and transcription factor expression for CD4$^+$ T-cells from CLL B-cell/T-cell co-cultures.

Patient-derived CLL B-cells/healthy donor T-cell co-cultures were incubated for 48 h with α-CD3/α-CD28 and OPN-51107 (OPN5; 0.1 or 0.5 μM), ibrutinib (IBR; 1 μM) or vehicle equivalent (VEH; DMSO), then evaluated by flow cytometry for surface expression of immune inhibitory receptors (IRs; n=10) and intranuclear expression of the indicated transcription factors (TFs; n=6). **(A)** Percentages of CD4$^+$ T-cells from patient-derived CLL B-cell co-cultures expressing the indicated IRs, normalized to VEH-treated T-cell mono-culture average. **(B)** Distribution of co-cultured CD4$^+$ T-cells into progenitor exhausted (T_{PEX}; PD-1^{int}/TIM3$^{lo/-}$) and terminally exhausted (T_{TEX}; PD-1^{hi}/TIM3^{hi}) T-cell subsets. **(C)** Percentages of stem-like (Stem; PD-1$^+$/TIM3$^-$/TCF-1$^+$) and terminally differentiated (TD; PD-1$^+$/TIM3$^+$/TCF-1$^-$) CD4$^+$ T-cells in treated T-cell mono-cultures (TC) and CLL B-cell co-cultures (TC + CLL). **(D)** Percentages of CD4$^+$ T-cells expressing RUNX3, T-BET, BATF, and TCF-1, normalized to TC VEH average. Data is represented as mean ± standard error of the mean. Significant difference from VEH was calculated using one-way ANOVA. *$p < 0.05$, **$p < 0.01$, ***$p < 0.001$.

Supplemental Figure 14

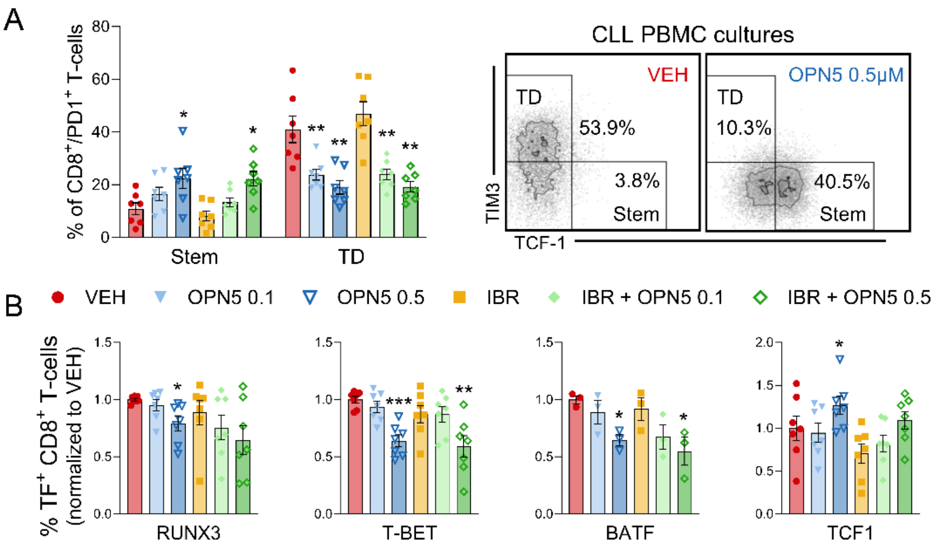

Supplemental Figure 14. CD8⁺ T-cell transcription factor expression from CLL patient PBMC cultures treated ex vivo.

CLL patient-derived PBMCs (consisting of 65.2 ± 0.06% CLL B-cells and 26.4 ± 0.04% T-cells) were stimulated with α-CD3/α-CD28 and treated with OPN-51107 (OPN5; 0.1 or 0.5 μM), ibrutinib (IBR; 1 μM), or the indicated combinations of OPN5 and IBR for 48 h (n=7). **(A)** Percentages of stem-like (Stem; PD-1^{+}/TIM3^{-}/TCF-1^{+}) and terminally differentiated (TD; PD-1^{+}/TIM3^{+}/TCF-1^{-}) CD8^{+} T-cells. **(B)** Percentages of CD8^{+} T-cells expressing RUNX3, T-BET, BATF, and TCF1, normalized to VEH average. Data are represented as mean ± standard error of the mean. Significant difference from VEH was calculated using one-way ANOVA. *p < 0.05, **p < 0.01, ***p < 0.001.

Supplemental Figure 15

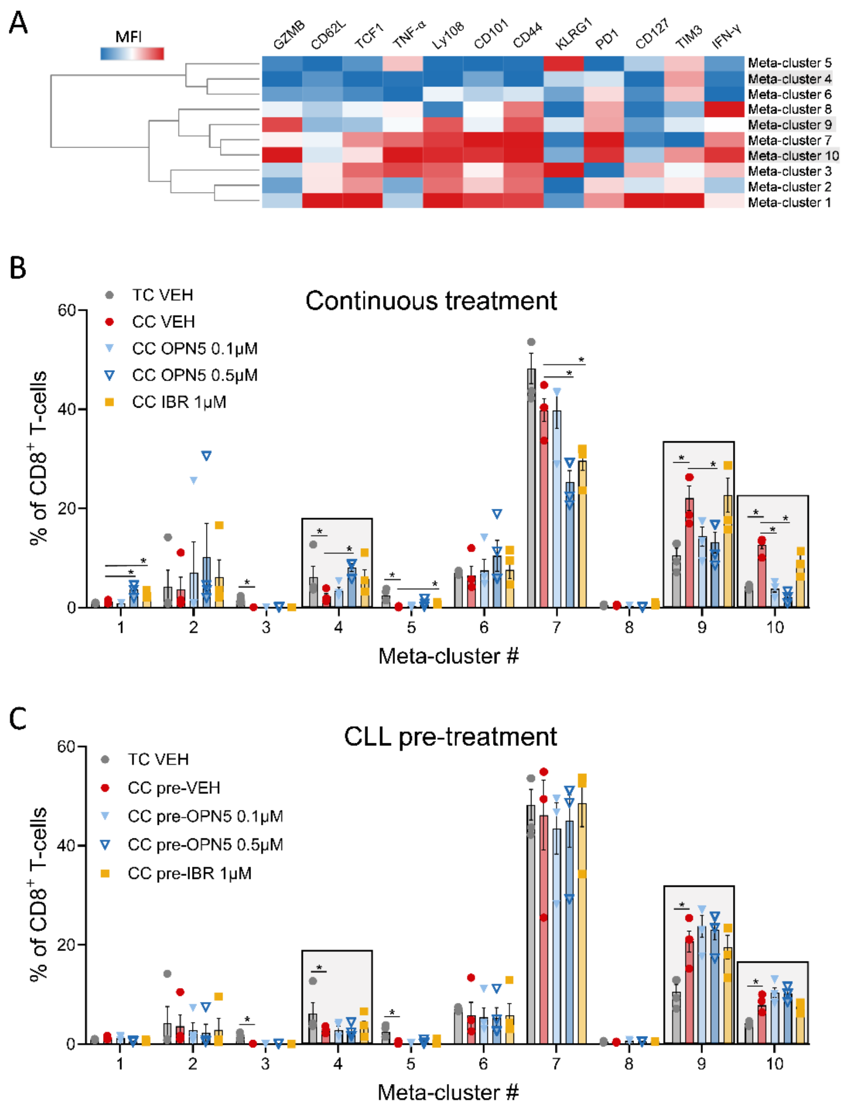

Supplemental Figure 15. FlowSOM clustering of CD8^{+} T-cells from CLL/T-cell co-cultures. Healthy donor T-cell alone (TC) or patient-derived CLL B-cells/healthy donor T-cell co-cultures (CC) were incubated for 48h, stimulated with α-CD3/α-CD28 (n=4) and probed for the indicated markers **(A)**. Cultures were either treated continuously **(B)** with OPN-51107 (OPN5; 0.1 or 0.5 μM), ibrutinib (IBR; 1 μM), or vehicle equivalent (VEH; DMSO) or CLL B-cells were pre-treated, and drug was washed off prior co-culture **(C)**. **(A)** Heatmap showing relative expression of each marker (columns), for each identified meta-cluster (rows). **(B)** Fold change in the percentage of CD8^{+} T-cells from continuously treated co-cultures found in each FlowSOM-identified meta-cluster. **(C)** Fold change in the percentage of CD8^{+} T-cells from CLL pre-treated co-cultures found in each FlowSOM-identified meta-cluster. Data is represented as mean ± standard error of the mean. MFI: median fluorescent intensity. Significant differences were calculated using one-way ANOVA for each meta-cluster. *$p < 0.05$.

Supplemental Figure 16

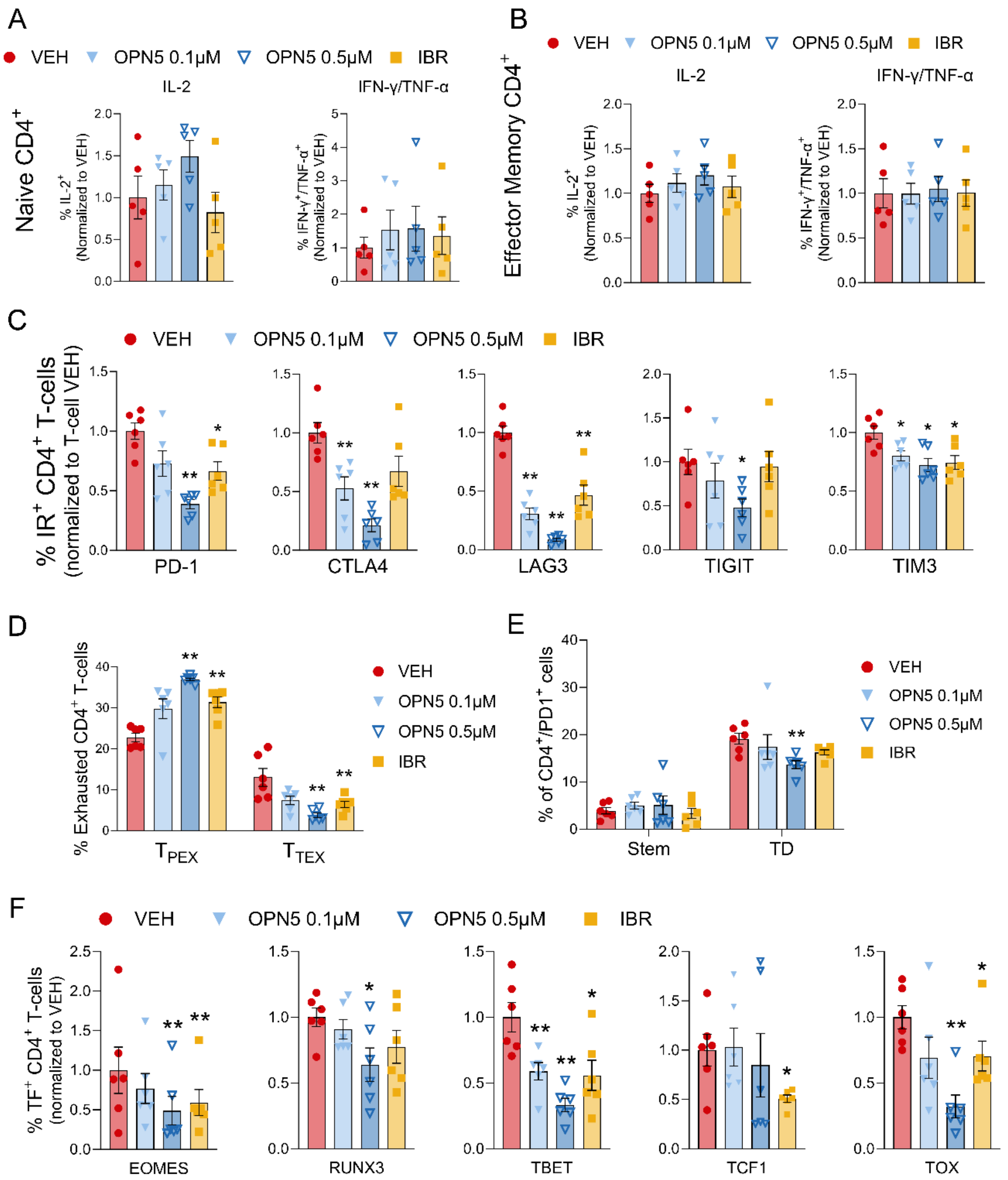

Supplemental Figure 16. Flow cytometry data for $CD4^+$ T-cells from CLL patient T-cells treated alone.

(A-B) CLL patient-derived T-cells treated with the OPN-51107 (OPN5; 0.1-0.5 μM), ibrutinib (IBR; 1 μM), or vehicle equivalent (VEH; DMSO) for 48 h, stimulated with PMA/ionomycin for the final 6 h then evaluated by flow cytometry for percentage of IL-2^+ and IFN-γ^+/TNF-α^+ naïve ($CD45RA^+$/$CCR7^+$, **A**) and effector memory ($CD45RA^-$/$CCR7^-$, **B**) $CD4^+$ T-cells, normalized to VEH (n=5). **(C-F)** CLL patient-derived T-cells treated with the indicated inhibitors in the presence of α-CD3/α-CD28 stimuli for 48 h (n=6). **(C)** Percentages of $CD4^+$ T-cells expressing immune inhibitory receptors (IRs) PD-1, LAG3, CTLA4, TIM3, and TIGIT, normalized to VEH. **(D)** Distribution of $CD4^+$ T-cells into progenitor exhausted (T_{PEX}; PD-1^{int}/$TIM3l^{o/-}$) and terminally exhausted (T_{TEX}; PD-1^{hi}/$TIM3^{hi}$) subsets. **(E)** Percentages of stem-like (Stem; PD-1^+/$TIM3^-$/TCF-1^+) and terminally differentiated (TD; PD-1^+/$TIM3^+$/TCF-1^-) $CD4^+$ T-cells. **(F)** Percentages of $CD4^+$ T-cells expressing transcription factors (TFs) EOMES, RUNX3, T-BET, TCF-1, and TOX, normalized to VEH. Data is represented as mean ± standard error of the mean. Significant difference from VEH was calculated using one-way ANOVA. *p < 0.05, **p < 0.01.

Supplemental Figure 17

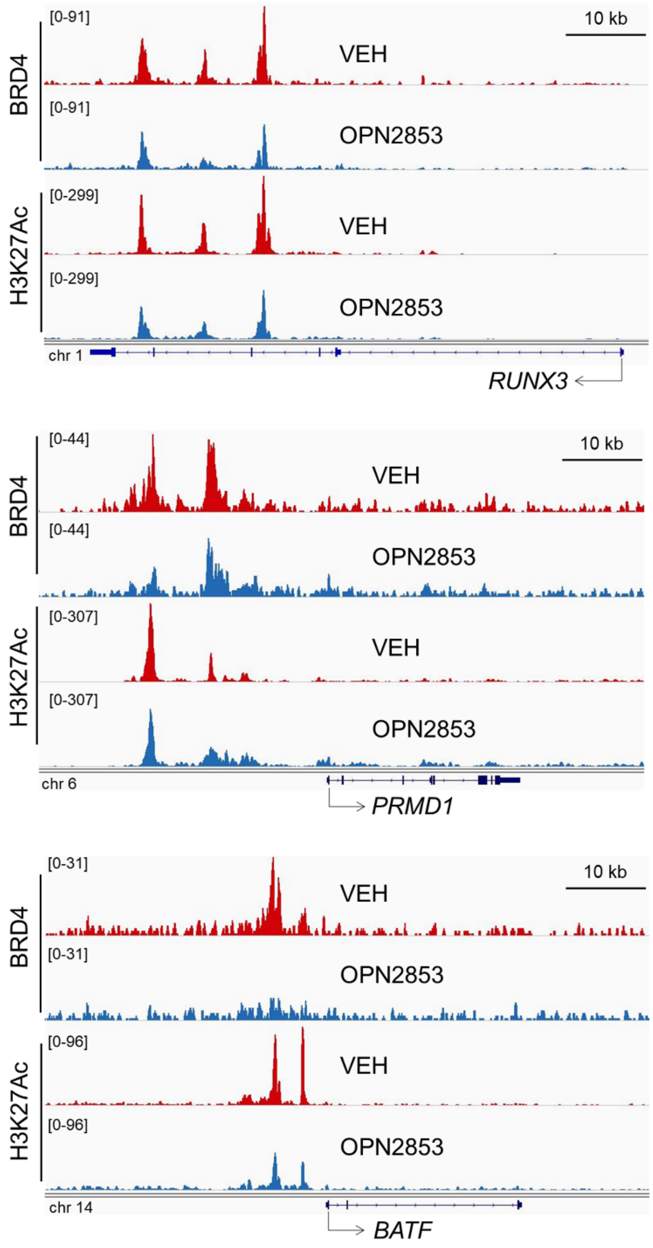

Supplemental Figure 17. Gene tracks from published ChIP-seq analysis of T-cells treated with OPN-2853.

IGV genome browser screenshot showing ChIP-seq peaks for BRD4 and H3K27Ac at *RUNX3, PRDM1,* and *BATF* loci in healthy donor T-cells stimulated with α-CD3/α-CD28 and treated with 10 nM OPN-2853 or vehicle equivalent (VEH) for 24 h (Snyder, et al. 2021). ChIP-seq track height is noted as a range [0-maximum].

Supplemental Figure 18

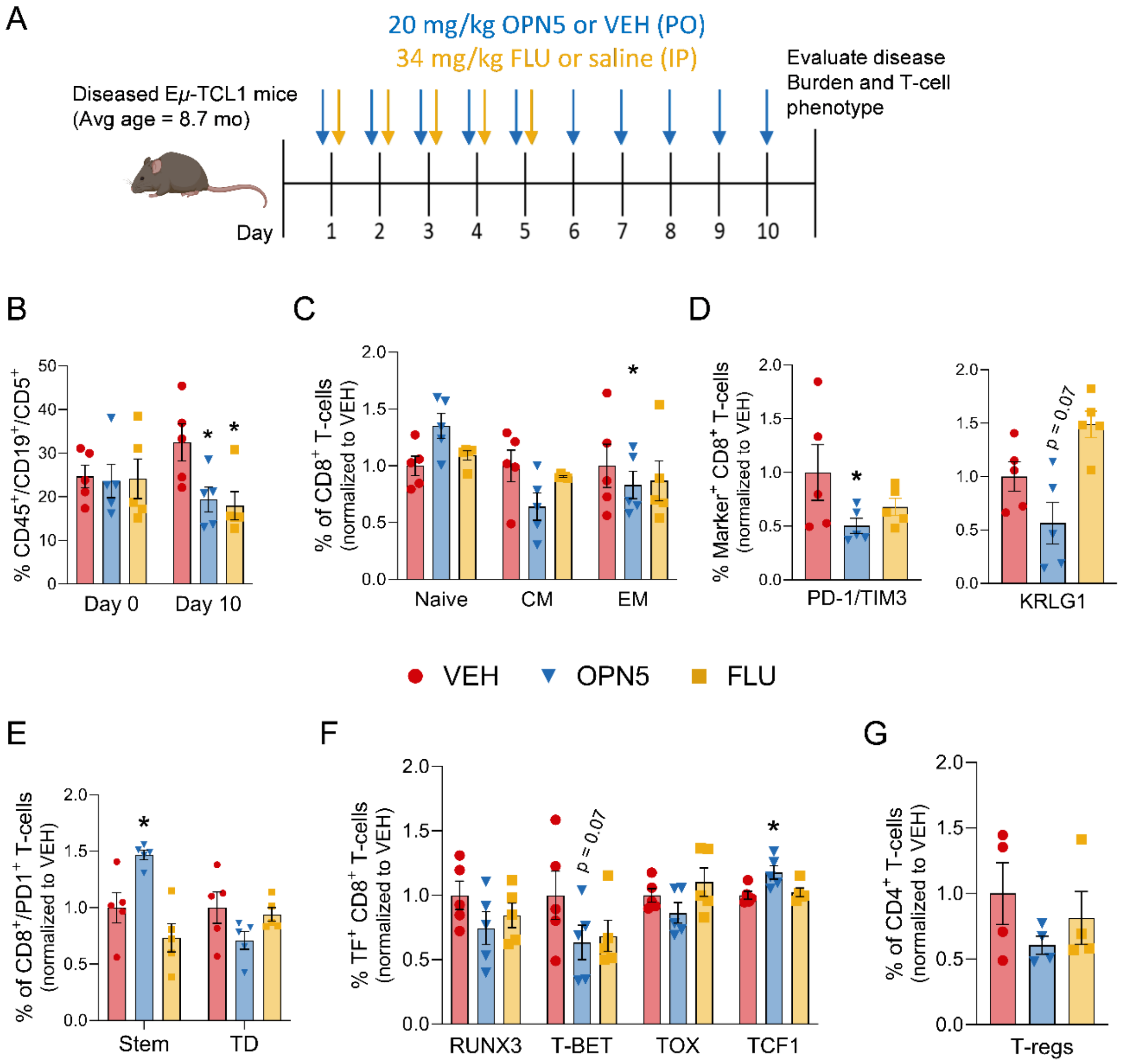

Supplemental Figure 18. Fludarabine reduces CLL burden like OPN-51107 but does not elicit comparable T-cell effects.

(A) Diseased Eμ-TCL1 mice (average age = 8.7 mo) were randomly assigned to treatment with OPN-51107 (OPN5, n=5), fludarabine phosphate (FLU, n=5) or vehicle equivalent (VEH, n=5). **(B)** Flow cytometry evaluation of disease burden in murine peripheral blood. **(C)** Distribution of splenic $CD8^+$ T-cells into naïve ($CD44^-/CD62L^+$, T_N), central memory ($CD44^+/CD62L^+$, T_{CM}), and effector memory ($CD44^+/CD62L^-$, T_{EM}) subsets, normalized to the average of VEH-treated mice. **(D)** Percentages of splenic $CD8^+$ T-cells expressing the indicated markers, normalized to the average of VEH-treated mice **(E)** Percentages of stem-like (Stem; $PD\text{-}1^+/TIM3^-/TCF\text{-}1^+$) and terminally differentiated (TD; $PD\text{-}1^+/TIM3^+/TCF\text{-}1^-$) splenic $CD8^+$ T-cells, normalized to the average of VEH-treated mice. **(F)** Percentages of splenic $CD8^+$ T-cells expressing the indicated transcription factors (TFs), normalized to the average of VEH-treated mice. **(G)** Percentage of splenic T-regs gated as $CD3^+/CD4^+/CD25^+/CD127^-$ cells, normalized to the average of VEH-treated mice. Summary data are represented as mean ± standard error of the mean. Significant difference from the VEH group was calculated using one-way ANOVA *$p < 0.05$.

Supplemental Figure 19

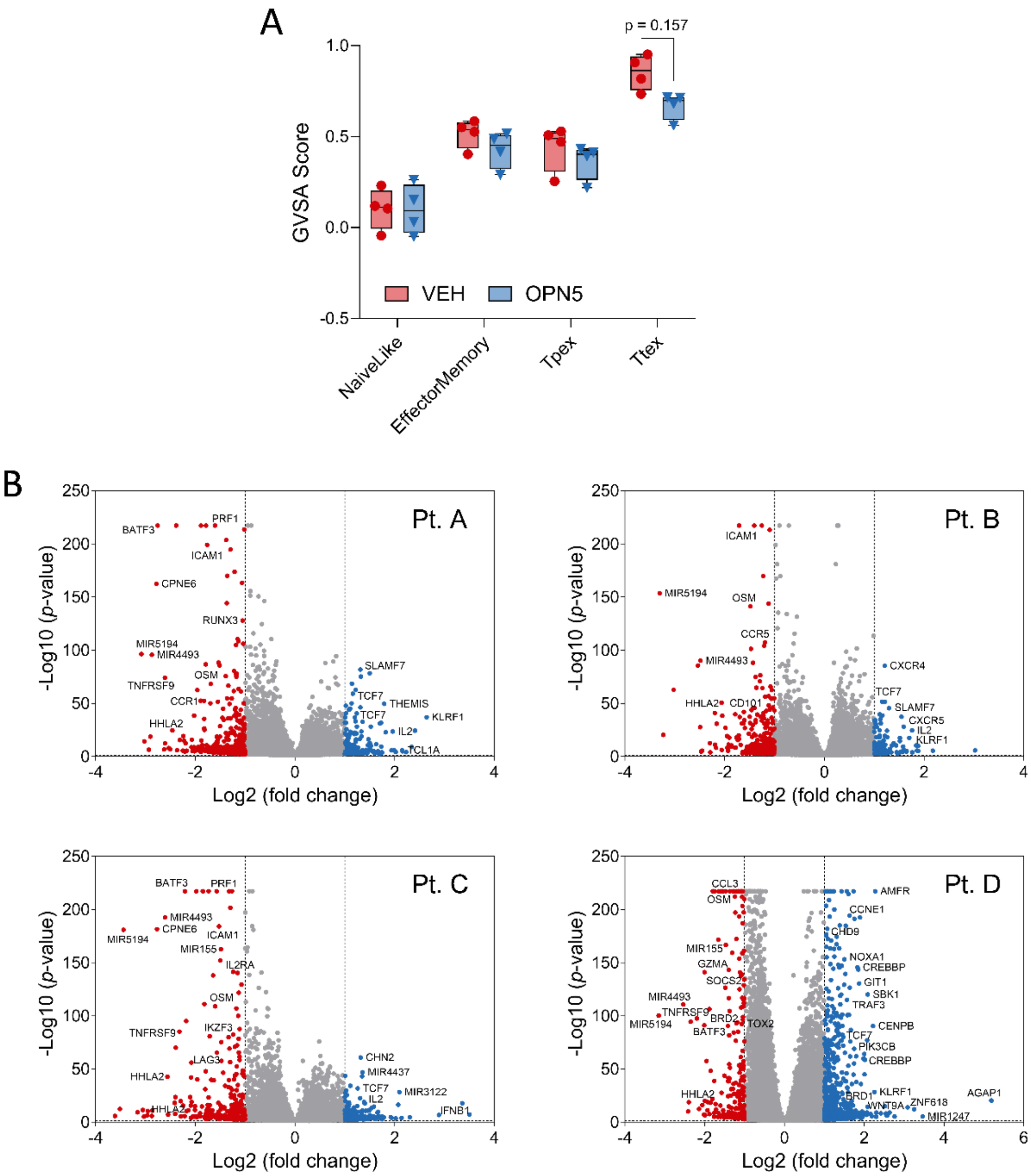

Supplemental Figure 19. Bulk ATAC-seq of CD8$^+$ T-cells isolated from CLL patient PBMCs treated ex vivo with OPN-51107.

PBMCs were treated with 0.5µM OPN-51107 (OPN5) or equivalent vehicle (DMSO) for 16 h, in the presence of α-CD3/α-CD28 stimuli (n=4). **(A)** Enrichment of T-cell signatures based on RNA-seq data from Andreatta et al. 2021. Significant difference between groups was assessed using Student's paired t-test. **(B)** Differential promoter peaks identified for each patient sample (OPN5 vs. VEH).

Supplemental Figure 20

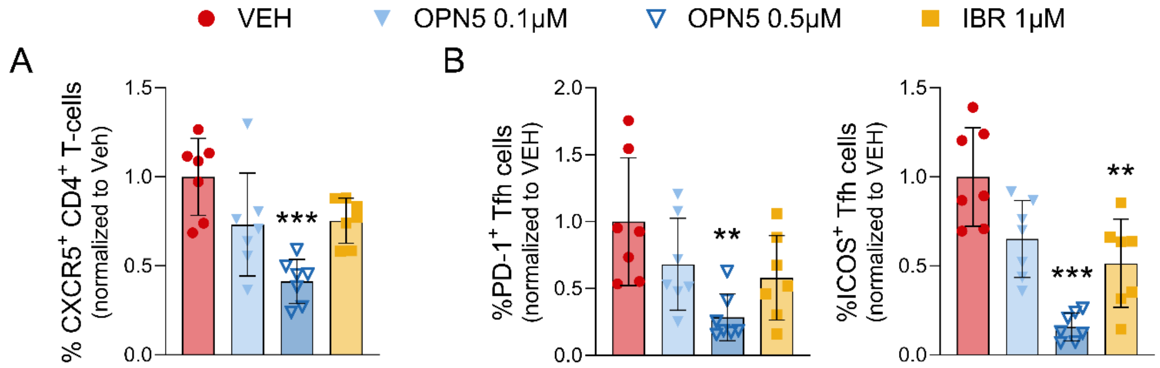

Supplemental Figure 20. T follicular helper cell populations in CLL patient PBMCs treated ex vivo with OPN-51107.

CLL patient-derived PBMCs were stimulated with α-CD3/α-CD28 and treated with OPN-51107 (OPN5; 0.1 or 0.5 μM), ibrutinib (IBR; 1 μM), or vehicle equivalent (VEH; DMSO) for 48 h (n=7). **(A)** Percentages of $CXCR5^{+}$ $CD4^{+}$ T follicular helper (Tfh) cells. **(B)** Percentage of Tfh cell expressing PD-1 and ICOS. Data are represented as mean ± standard error of the mean. Significant difference from VEH was calculated using one-way ANOVA. **p < 0.01, ***p < 0.001.

II. Supplemental Tables

Supplemental Table 1. Characteristics of CLL[#] patient-derived samples used in ex vivo assays.

ID #	Age	Sex	IgHV mutational status	Therapy status (Naïve or TT)	FISH cytogenetics					Karyotype (Normal or Complex[‡])	Figures
					del 13q	del 17p	del 11p	del 6q	Trisomy 12		
001	66	M	NA	Naïve	NA	NA	NA	NA	NA	NA	4A & S9-11A; 5A-C & S13A-B
002	78	F	Mutated (borderline*)	TT	pos	neg	pos	neg	neg	Complex	4A & S9-11A; 5A-C & S13A-B
003	66	M	NA	Naïve	NA	NA	NA	NA	NA	NA	4A & S9-11A; 5A-E & S13
004	68	F	Unmutated	TT	pos	neg	pos	neg	neg	NA	4 & S9-11; 5A-E & S13
005	65	M	Unmutated	Naïve	neg	neg	neg	neg	neg	NA	4 & S9-11; 5A-E & S13
006	84	F	NA	TT	pos	neg	pos	neg	pos	NA	4 & S9-11; 5A-E & S13
007	66	M	Mutated	TT	neg	neg	neg	neg	neg	NA	4 & S9-11; 5D-E & S13C-D
008	45	M	Unmutated	TT	pos	neg	neg	neg	neg	Normal	4B-D & S9-11B-D
009	70	M	Unmutated	Naïve	neg	neg	neg	neg	neg	Normal	5A-C & S13A-B
010	76	M	Unmutated	TT	neg	pos	neg	neg	neg	Complex	5A-C & S13A-B
011	67	F	Mutated	TT	pos	neg	neg	neg	neg	Normal	5A-C & S13A-B
012	53	F	Unmutated	Naïve	pos	neg	neg	neg	neg	Normal	5A-C & S13A-B
013	78	M	Mutated	Naïve	pos	neg	neg	neg	neg	NA	5A-E & S13
014	68	M	Unmutated	Naïve	neg	neg	neg	pos	neg	NA	5D-E & S13C-D; 5F & S12, S15
015	68	M	NA	Naïve	pos	neg	neg	neg	neg	Normal	5F & S12, S15
016	50	M	Mutated	Naïve	pos	neg	neg	neg	neg	Normal	5F & S12, S15
017	76	F	Mutated	Naïve	neg	neg	neg	neg	neg	Normal	5F & S12, S15
018	59	M	Unmut	Naïve	pos	neg	pos	neg	neg	NA	5F & S12, S15
019	52	M	Unmutated	Naïve	neg	neg	neg	neg	neg	NA	S14; S20
020	63	M	Mutated	Naïve	pos	neg	neg	neg	neg	NA	S14; 7 & S19[Δ]; S20
021	50	M	NA	TT	pos	neg	neg	neg	neg	Complex	S14; S20
022	60	M	Mutated	Naïve	neg	neg	neg	neg	pos	Normal	S14; S20
023	82	M	NA	Naïve	NA	NA	NA	NA	NA	NA	S14; S20
024	61	F	NA	Naïve	NA	NA	NA	NA	NA	NA	S14; S20
025	71	F	NA	Naïve	pos	neg	neg	neg	neg	Normal	S14; S20
026	88	M	Unmutated	TT	neg	neg	neg	neg	pos	Complex	S14; S20
027	52	M	NA	Naive	NA	NA	NA	NA	NA	NA	6A, C-F & S16
028	67	M	NA	TT	pos	pos	neg	neg	neg	NA	6A, C-F & S16
029	77	M	NA	TT	neg	neg	neg	neg	neg	Normal	6B
030	56	F	NA	Naive	NA	NA	NA	NA	NA	NA	6B
031	60	M	NA	Naive	neg	neg	neg	neg	pos	Normal	6 & S16; 7 & S19[Δ]
032	67	F	NA	TT	neg	neg	pos	pos	pos	Complex	6 & S16
033	78	M	Mutated	Naïve	pos	neg	neg	neg	neg	Normal	6 & S16
034	65	M	Unmut	Naive	pos	neg	neg	neg	neg	Normal	6 & S16; 7& S19[Δ]
035	69	M	Unmut	Naive	neg	neg	neg	neg	neg	Normal	7 & S19[Δ]

[#] CLL diagnosis was determined per iwCLL 2018 guidelines[1]; ***** with VH segment % homology of 97.54; **‡** complex karyotype defined as harboring 3 or more chromosome abnormalities[2]. [Δ] ATAC-sequencing samples (#020 = Pt A; #031 = Pt D; #034 = Pt C; #035 = Pt B).

IgHV, immunoglobulin heavy chain variable region; TT, treated; FISH, fluorescence in situ hybridization; del, deletion; M, male; F, female; pos, positive; neg, negative; NA, not available.

Supplemental Table 2. Modulated canonical pathways in the spleen microenvironment following BET inhibitor treatment of leukemic mice (adoptive transfer Eμ-TCL1) identified by IPA using NanoString PanCancer iO360 expression panel ($p < 0.001$).

Ingenuity Canonical Pathways	-Log(p-value)	Z-score*	Molecules
Th1 and Th2 Activation Pathway	2.70E+01	NA	CD247, CD274, CD4, CD40, CD80, CD86, CXCR4, GATA3, HLA-A, HLA-DMA, HLA-DMB, HLA-DQA1, HLA-DQB1, HLA-DRB5, ICOSLG/LOC102723996, IFNAR1, IFNGR1, IKZF1, IL10, IL18, IL24, IL2RB, JAK1, JAK2, JAK3, KLRD1, NFATC2, NOTCH1, PIK3CG, PIK3R1, STAT3, TGFB1, TGFBR1, TNFRSF4
PD-1, PD-L1 cancer immunotherapy pathway	2.37E+01	1.633	B2M, CD247, CD274, CD80, HLA-A, HLA-DMA, HLA-DMB, HLA-DQA1, HLA-DQB1, HLA-DRB5, HLA-E, HLA-G, IFNGR1, IL2RB, JAK1, JAK2, JAK3, PDCD1LG2, PIK3CG, PIK3R1, PTEN, PTPN11, TGFB1, TNFRSF1A, TNFRSF1B, ZAP70
Natural Killer Cell Signaling	2.32E+01	0.18	B2M, CD247, CD48, COL11A2, FASLG, FCGR3A/FCGR3B, FYN, HLA-A, HLA-E, HLA-G, IL15, IL18, IL2RB, ITGAL, JAK2, JAK3, KLRD1, LAIR1, MAP3K12, MAP3K5, MICB, NCR1, NFATC2, NRAS, PIK3CG, PIK3R1, PTPN11, RELB, ROCK1, TNFSF10, ULBP1, ZAP70
Th1 Pathway	2.30E+01	0.471	CD247, CD274, CD4, CD40, CD80, CD86, GATA3, HLA-A, HLA-DMA, HLA-DMB, HLA-DQA1, HLA-DQB1, HLA-DRB5, ICOSLG/LOC102723996, IFNAR1, IFNGR1, IL10, IL18, JAK1, JAK2, JAK3, KLRD1, NFATC2, NOTCH1, PIK3CG, PIK3R1, STAT3
Th2 Pathway	2.29E+01	-0.2	CD247, CD4, CD40, CD80, CD86, CXCR4, GATA3, HLA-A, HLA-DMA, HLA-DMB, HLA-DQA1, HLA-DQB1, HLA-DRB5, ICOSLG/LOC102723996, IKZF1, IL10, IL24, IL2RB, JAK1, JAK2, JAK3, NFATC2, NOTCH1, PIK3CG, PIK3R1, TGFB1, TGFBR1, TNFRSF4
Crosstalk between Dendritic Cells and Natural Killer Cells	2.26E+01	-2.236	CD40, CD69, CD80, CD86, CSF2RB, FASLG, HLA-A, HLA-DRB5, HLA-E, HLA-G, IL15, IL18, IL2RB, ITGAL, KLRD1, LTB, MICB, RELB, TLR4, TLR7, TLR9, TNFRSF1B, TNFSF10
Role of Pattern Recognition Receptors in Recognition of Bacteria and Viruses	2.20E+01	0.229	C1QA, C1QB, C5AR1, CASP1, FASLG, IL10, IL15, IL17A, IL18, IL1B, IRF3, IRF7, LTB, NOD2, PIK3CG, PIK3R1, PRKCA, RELB, RIPK2, TGFB1, TICAM1, TLR1, TLR2, TLR4, TLR7, TLR9, TNFSF10, TNFSF13, TNFSF8
Pulmonary Healing Signaling Pathway	2.02E+01	0.18	BLK, CCNB1, CDC25C, CDH1, CTNNB1, CXCR4, FYN, FZD9, HCK, KDR, MMP12, MMP13, MMP9, MYC, NFKBIA, NOTCH1, NRAS, PECAM1, PRKAA2, PRKCA, SMAD5, STAT3, TGFB1, THBS1, TLR2, TLR4, TNFRSF1A, TNFRSF1B, VEGFB, WNT10A, WNT5A
Glucocorticoid Receptor Signaling	1.98E+01	NA	ARID1A, B2M, BRCA1, CD247, CDKN1A, CHUK, CSF2RB, DUSP1, ERCC3, ESR1, HLA-A, HLA-DMA, HLA-DMB, HLA-DQA1, HLA-DQB1, HLA-DRB5, HLA-E, HLA-G, IFNAR1, IL10, IL1B, IL2RB, IL7R, IRF3, JAK1, JAK2, JAK3, MMP13, MMP9, MYC, NFATC2, NFKBIA, NRAS, PC, PCK2, PIK3CG, PIK3R1, POLR2A, PRKAA2, PRKACB, SDHA, SGK1, STAT3, TGFB1, TGFBR1, TLR2, VCAM1
Tumor Microenvironment Pathway	1.84E+01	0.577	BAD, CD274, CTLA4, CXCR4, FASLG, FGF9, HLA-A, HLA-E, HLA-G, IL10, IL1B, ITGB3, JAK2, MMP12, MMP13, MMP9, MYC, NRAS, PDCD1LG2, PIK3CG, PIK3R1, RELB, SLC2A1, SPP1, STAT3, TGFB1, TNFRSF1A, VEGFB
Antigen Presentation Pathway	1.72E+01	NA	B2M, HLA-A, HLA-DMA, HLA-DMB, HLA-DQA1, HLA-DQB1, HLA-DRB5, HLA-E, HLA-G, PSMB5, PSMB8, PSMB9, TAP1, TAP2
Granulocyte Adhesion and Diapedesis	1.69E+01	NA	C5AR1, CCL19, CCL22, CCL28, Ccl9, CCR2, CCR9, CDH5, CSF3R, CXCL13, CXCL14, Cxcl15, CXCR4, CXCR5, IL18, IL1B, ITGA4, ITGAM, MMP12, MMP13, MMP9, PECAM1, PF4, TNFRSF1A, TNFRSF1B, VCAM1
Regulation Of The Epithelial Mesenchymal Transition By Growth Factors Pathway	1.65E+01	-0.6	CDH1, CHUK, ERBB2, FASLG, FGF9, JAK1, JAK2, JAK3, LTB, MET, MMP9, NRAS, PDGFRB, PIK3CG, PIK3R1, PTPN11, RELB, STAT3, TGFB1, TGFBR1, TNFRSF1A, TNFRSF1B, TNFSF10, TNFSF13, TNFSF18, TNFSF8, ZEB2
T Cell Exhaustion Signaling Pathway	1.62E+01	-1	CD247, CD274, CD80, CD86, CTLA4, HLA-A, HLA-DMA, HLA-DMB, HLA-DQA1, HLA-DQB1, HLA-DRB5, HLA-E, HLA-G, IFNAR1, IL10, IRF4, JAK1, JAK2, JAK3, KDR, LAG3, NFATC2, NRAS, PDCD1LG2, PDK1, PIK3CG, PIK3R1, PRDM1, PTPN11, STAT2, STAT3, TGFB1, TGFBR1, TNFRSF14, ZAP70

ID1 Signaling Pathway	1.61E+01	0.192	ATF3, BAX, BIRC5, BLK, CCNE1, CDC20, CDKN1A, CDKN2A, CTNNB1, FYN, HCK, MMP9, MYC, NRAS, PIK3CG, PIK3R1, PTEN, PTGER4, S100A9, SMAD5, STAT3, TGFB1, TGFBR1, TNFRSF1A, TNFRSF1B, TYMS, VEGFB
Role of PKR in Interferon Induction and Antiviral Response	1.56E+01	-0.229	ATF3, BAX, CASP1, CASP8, CHUK, FASLG, IFNAR1, IFNGR1, IL18, IL1B, IL24, IRF3, JAK1, MARCO, NFKBIA, PDGFRB, RELB, STAT2, STAT3, TLR4, TLR9, TNFRSF1A
HOTAIR Regulatory Pathway	1.53E+01	-0.626	CDH1, CDKN1A, CTNNB1, ERBB2, ESR1, EZH2, MET, MMP12, MMP13, MMP9, MYC, NFKBIA, PIK3CG, PIK3R1, PTEN, RELB, ROCK1, SPP1, STAT3, TGFB1, TLR4, TWIST2, WNT10A, WNT5A
B Cell Development	1.52E+01	NA	CD19, CD40, CD79A, CD79B, CD80, CD86, HLA-A, HLA-DMA, HLA-DMB, HLA-DQA1, HLA-DQB1, HLA-DRB5, IL7R
Dendritic Cell Maturation	1.45E+01	-1.567	B2M, CD247, CD40, CD80, CD86, CHUK, COL11A2, FCGR2B, FCGR3A/FCGR3B, HLA-A, HLA-DMA, HLA-DMB, HLA-DQA1, HLA-DQB1, HLA-DRB5, HLA-E, HLA-G, IFNAR1, IL10, IL15, IL18, IL1B, JAK2, LTB, NFKBIA, PIK3CG, PIK3R1, RELB, STAT2, TLR2, TLR4, TLR9, TNFRSF1A, TNFRSF1B
TREM1 Signaling	1.44E+01	-1.5	CASP1, CD40, CD86, FCGR2B, IL10, IL18, IL1B, JAK2, NOD2, RELB, STAT3, TLR1, TLR2, TLR4, TLR7, TLR9, TREM1
PI3K/AKT Signaling	1.40E+01	0.535	BAD, CDKN1A, CHUK, CSF2RB, CTNNB1, EIF4EBP1, IFNAR1, IL2RB, IL7R, ITGA4, ITGA6, ITGAL, ITGAM, ITGB3, ITGB8, JAK1, JAK2, JAK3, MAP3K5, NFKBIA, NRAS, PIK3CG, PIK3R1, PTEN, RELB
Agranulocyte Adhesion and Diapedesis	1.35E+01	NA	C5AR1, CCL19, CCL22, CCL28, Ccl9, CCR2, CCR9, CDH5, CXCL13, CXCL14, Cxcl15, CXCR4, CXCR5, IL18, IL1B, ITGA4, ITGA6, MMP12, MMP13, MMP9, PECAM1, PF4, TNFRSF1A, VCAM1
Regulation of the Epithelial-Mesenchymal Transition Pathway	1.33E+01	NA	AXIN1, CDH1, CTNNB1, FGF9, FZD9, JAK1, JAK2, JAK3, MET, MMP9, NOTCH1, NRAS, PDGFRB, PIK3CG, PIK3R1, PTPN11, RELB, STAT3, TGFB1, TGFBR1, TWIST2, WNT10A, WNT5A, ZEB2
Pyroptosis Signaling Pathway	1.32E+01	-2.668	BAX, CASP1, CASP8, GBP2, GBP4, IL18, IL1B, IRF2, PRKACB, PTGER4, TLR1, TLR2, TLR4, TLR7, TLR9, TNFRSF1A, TNFRSF1B
Wound Healing Signaling Pathway	1.29E+01	0	CHUK, COL11A2, FASLG, IFNGR1, IL15, IL17A, IL18, IL1B, ITGA6, JAK1, JAK2, LAMB3, LTB, MMP9, NFKBIA, NRAS, PF4, PRKCA, TGFB1, TGFBR1, TNFRSF1A, TNFRSF1B, TNFSF10, TNFSF13, TNFSF8, VEGFB
Senescence Pathway	1.29E+01	1.177	ATF3, CCNB1, CCND3, CCNE1, CDC25C, CDK6, CDKN1A, CDKN2A, CHUK, E2F3, EIF4EBP1, EZH2, IRF3, MRE11, NBN, NF1, NFATC2, NRAS, PDK1, PIK3CG, PIK3R1, PTEN, RB1, SMAD5, TGFB1, TGFBR1, TLR2, VHL
HIF1α Signaling	1.20E+01	-1.043	ADM, CDKN1A, CYBB, EIF4EBP1, HK1, IL17A, KDR, LDHA, LDHB, MET, MMP12, MMP13, MMP9, NRAS, PIK3CG, PIK3R1, PKM, PRKCA, SLC2A1, STAT3, TGFB1, VEGFB, VHL
IL-15 Production	1.17E+01	-0.243	AXL, BLK, CSF1R, ERBB2, FYN, HCK, IL15, IRF3, JAK1, JAK2, JAK3, KDR, MET, PDGFRB, RELB, TIE1, TWF1, ZAP70
MSP-RON Signaling In Macrophages Pathway	1.14E+01	0	CHUK, HLA-DMA, HLA-DMB, HLA-DQA1, HLA-DQB1, HLA-DRB5, IFNGR1, IL10, ITGAM, JAK2, NRAS, PIK3CG, PIK3R1, RELB, SBNO2, STAT3, TLR4
IL-4 Signaling	1.11E+01	NA	HLA-A, HLA-DMA, HLA-DMB, HLA-DQA1, HLA-DQB1, HLA-DRB5, HMGA1, IRF4, JAK1, JAK2, JAK3, NFATC2, NRAS, PIK3CG, PIK3R1
IL-13 Signaling Pathway	1.11E+01	1	BLK, CD36, CTNNB1, DUSP1, FYN, HCK, IL10, IL17A, IL24, JAK1, JAK2, PIK3CG, PIK3R1, ROCK1, STAT3, TGFB1
Erythropoietin Signaling Pathway	1.09E+01	1.606	BAD, CSF2RB, CTNNB1, FASLG, IL15, IL17A, IL18, IL1B, JAK2, LTB, NFKBIA, NRAS, PIK3CG, PIK3R1, PRKCA, RELB, TGFB1, TNFSF10, TNFSF13, TNFSF8
T Cell Receptor Signaling	1.06E+01	-0.365	B2M, CD247, CD4, CD80, CD86, CHUK, CTLA4, CTNNB1, DUSP5, FYN, HLA-A, HLA-DMA, HLA-DMB, HLA-DQA1, HLA-DQB1, HLA-DRB5, HLA-E, HLA-G, ICOSLG/LOC102723996, ITGAL, NFATC2, NFKBIA, NRAS, PDK1, PIK3CG, PIK3R1, PTPN11, RELB, RPTOR, ZAP70
Caveolar-mediated Endocytosis Signaling	1.06E+01	NA	B2M, CD48, FLNB, FYN, HLA-A, HLA-E, HLA-G, ITGA4, ITGA6, ITGAL, ITGAM, ITGB3, ITGB8, PRKCA
HMGB1 Signaling	1.05E+01	0.905	FASLG, IFNGR1, IL15, IL17A, IL18, IL1B, LTB, NRAS, PIK3CG, PIK3R1, RELB, TGFB1, TLR4, TNFRSF1A, TNFRSF1B, TNFSF10, TNFSF13, TNFSF8, VCAM1
IL-17 Signaling	1.04E+01	-1.606	CCL22, FASLG, IL15, IL17A, IL18, IL1B, JAK1, JAK2, LTB, MMP13, MMP9, NRAS, PIK3CG, PIK3R1, TGFB1, TNFSF10, TNFSF13, TNFSF8, VEGFB
Production of Nitric Oxide and Reactive Oxygen Species in Macrophages	1.03E+01	0.229	APOE, CHUK, CYBB, IFNGR1, JAK1, JAK2, JAK3, MAP3K12, MAP3K5, NFKBIA, PIK3CG, PIK3R1, PRKCA,

			RELB, S100A8, SIRPA, TLR2, TLR4, TNFRSF1A, TNFRSF1B
Necroptosis Signaling Pathway	1.02E+01	0.943	AXL, CASP1, CASP8, CHUK, CYBB, FASLG, GLUD1, IFNAR1, IRF3, JAK1, RB1, RIPK1, STAT2, TICAM1, TLR4, TNFRSF1A, TNFRSF1B, TNFSF10
Endocannabinoid Cancer Inhibition Pathway	1.02E+01	-1.414	ATF3, BAD, CASP1, CASP8, CCND3, CCNE1, CDH1, CDKN1A, CTNNB1, MYC, PIK3CG, PIK3R1, PRKAA2, PRKACB, ROCK1, RPTOR, TWIST2, VEGFB
HGF Signaling	9.97E+00	-0.577	CDKN1A, CDKN2A, ITGA4, ITGA6, ITGAL, ITGAM, ITGB3, ITGB8, MAP3K12, MAP3K5, MET, NRAS, PIK3CG, PIK3R1, PRKCA, PTPN11, STAT3
PTEN Signaling	9.88E+00	0.302	BAD, CDKN1A, CHUK, FASLG, ITGA4, ITGA6, ITGAL, ITGAM, ITGB3, ITGB8, KDR, NRAS, PDGFRB, PIK3CG, PIK3R1, PTEN, RELB, TGFBR1
Acute Phase Response Signaling	9.68E+00	0.535	C1QA, C1QB, C2, CHUK, CRABP2, IL18, IL1B, JAK2, MAP3K5, NFKBIA, NRAS, PIK3CG, PIK3R1, PTPN11, RELB, RIPK1, STAT3, TNFRSF1A, TNFRSF1B
iNOS Signaling	9.51E+00	NA	CD14, CHUK, HMGA1, IFNGR1, JAK1, JAK2, JAK3, LY96, NFKBIA, RELB, TLR4
PI3K Signaling in B Lymphocytes	9.35E+00	0.258	ATF3, BLK, CD19, CD40, CD79A, CD79B, CHUK, FCGR2B, FYN, NFATC2, NFKBIA, NRAS, PIK3CG, PIK3R1, PTEN, RELB, TLR4
Toll-like Receptor Signaling	9.34E+00	-0.905	CD14, CHUK, IL18, IL1B, LY96, NFKBIA, RELB, TICAM1, TLR1, TLR2, TLR4, TLR7, TLR9
IL-7 Signaling Pathway	9.12E+00	1.155	BAD, BAX, CCND3, CXCR5, FYN, IL7R, JAK1, JAK3, MET, MYC, PIK3CG, PIK3R1, SLC2A1
NF-κB Activation by Viruses	9.05E+00	1.155	CD4, CHUK, CXCR5, ITGA6, ITGB3, NFKBIA, NRAS, PIK3CG, PIK3R1, PRKCA, RELB, RIPK1, TNFRSF14
Interferon Signaling	8.71E+00	1	BAX, IFITM2, IFNAR1, IFNGR1, JAK1, JAK2, PSMB8, STAT2, TAP1
IL-12 Signaling and Production in Macrophages	8.64E+00	NA	APOE, CD40, CHUK, IFNGR1, IL10, IL18, NFKBIA, PIK3CG, PIK3R1, PRKCA, RELB, S100A8, TGFB1, TLR2, TLR4
T Helper Cell Differentiation	8.55E+00	NA	CD247, CD40, CD80, CD86, CXCR5, GATA3, HLA-A, HLA-DMA, HLA-DMB, HLA-DQA1, HLA-DQB1, HLA-DRB5, ICOSLG/LOC102723996, IFNGR1, IL10, IL17A, IL18, STAT3, TGFB1, TGFBR1, TNFRSF1A, TNFRSF1B
ERK/MAPK Signaling	8.32E+00	0	BAD, DUSP1, DUSP2, EIF4EBP1, ESR1, FYN, ITGA4, ITGA6, ITGAL, ITGAM, ITGB3, ITGB8, MYC, NRAS, PIK3CG, PIK3R1, PRKACB, PRKCA, STAT3
FAK Signaling	8.22E+00	-0.949	ADGRE1, ADORA2A, APLNR, BAD, C5AR1, CCR2, CCR9, CD247, CDH1, CDH5, CMKLR1, COL11A2, CSF2RB, CTNNB1, CX3CR1, ERBB2, FYN, FZD9, IFNAR1, IL2RB, IL7R, ITGA4, ITGA6, ITGAL, ITGAM, ITGB3, ITGB8, KDR, MET, MMP9, MYC, NRAS, P2RY13, PDGFRB, PIK3CG, PIK3R1, PTEN, PTGER4, TGFB1, TGFBR1
Role of Tissue Factor in Cancer	8.01E+00	NA	BLK, FYN, HCK, IL1B, ITGA6, ITGB3, JAK2, MMP13, NRAS, PIK3CG, PIK3R1, PRKCA, PTEN, PTPN11
Phagosome Formation	7.98E+00	-2.058	ADGRE1, ADORA2A, APLNR, C5AR1, CCR2, CCR9, CD14, CD36, CMKLR1, CX3CR1, FCGR2B, FCGR3A/FCGR3B, FYN, FZD9, HCK, ITGA4, ITGA6, ITGAL, ITGAM, ITGB3, ITGB8, MARCO, NRAS, P2RY13, PIK3CG, PIK3R1, PRKCA, PTGER4, ROCK1, TLR1, TLR2, TLR4, TLR7, TLR9
IL-17A Signaling in Airway Cells	7.96E+00	0	CHUK, IL17A, JAK1, JAK2, JAK3, NFKBIA, PIK3CG, PIK3R1, PTEN, RELB, STAT3
NF-κB Signaling	7.89E+00	-0.408	CASP8, CD247, CD40, CHUK, IL18, IL1B, KDR, NFKBIA, NRAS, PDGFRB, PIK3CG, PIK3R1, PRKACB, RELB, RIPK1, TGFBR1, TLR1, TLR2, TLR4, TLR7, TLR9, TNFRSF17, TNFRSF1A, TNFRSF1B, ZAP70
FAT10 Cancer Signaling Pathway	7.89E+00	3	CHUK, CTNNB1, CXCR4, NFKBIA, RELB, STAT3, TGFB1, TGFBR1, TNFRSF1A, TNFRSF1B
Role of BRCA1 in DNA Damage Response	7.86E+00	-1.265	ARID1A, BLM, BRCA1, BRCA2, BRIP1, CDKN1A, E2F3, MLH1, MRE11, NBN, RAD51, RB1
STAT3 Pathway	7.83E+00	0	CDKN1A, CSF2RB, IFNAR1, IL1B, IL2RB, IL7R, JAK2, KDR, MAP3K12, MYC, NRAS, PDGFRB, STAT3, TGFB1, TGFBR1
IL-8 Signaling	7.77E+00	0.5	BAX, CCND3, CDH1, CHUK, CYBB, EIF4EBP1, ITGAM, ITGB3, KDR, MMP9, NFKBIA, NRAS, PIK3CG, PIK3R1, PRKCA, ROCK1, VCAM1, VEGFB
Role of JAK1 and JAK3 in γc Cytokine Signaling	7.60E+00	NA	IL15, IL2RB, IL7R, JAK1, JAK2, JAK3, NRAS, PIK3CG, PIK3R1, PTPN11, STAT3
ICOS-ICOSL Signaling in T Helper Cells	7.59E+00	-0.471	BAD, CD247, CD4, CD40, CD80, CD86, CHUK, HLA-A, HLA-DMA, HLA-DMB, HLA-DQA1, HLA-DQB1, HLA-DRB5, ICOSLG/LOC102723996, IL2RB, NFATC2, NFKBIA, PIK3CG, PIK3R1, PTEN, RELB, ZAP70
Mouse Embryonic Stem Cell Pluripotency	7.58E+00	0.832	AXIN1, CTNNB1, FZD9, JAK1, JAK2, JAK3, MYC, NRAS, PIK3CG, PIK3R1, PTPN11, SMAD5, STAT3
Activation of IRF by Cytosolic Pattern Recognition Receptors	7.55E+00	1.667	CD40, CHUK, IFNAR1, IL10, IRF3, IRF7, NFKBIA, RELB, RIPK1, STAT2

Autophagy	7.55E+00	0.471	BAD, BNIP3, MYC, NOD2, PIK3CG, PIK3R1, PRKAA2, PRKACB, PTEN, RIPK1, RIPK2, RPTOR, SLC1A5, TGFB1, TICAM1, TLR4, TNFRSF1A, TNFRSF1B
Apoptosis Signaling	7.53E+00	-0.577	BAD, BAX, CASP8, CHUK, FASLG, MAP3K5, NFKBIA, NRAS, PRKCA, RELB, ROCK1, TNFRSF1A, TNFRSF1B
Leukocyte Extravasation Signaling	7.47E+00	0.5	CDH5, CTNNB1, CXCR4, CYBB, ITGA4, ITGAL, ITGAM, MMP12, MMP13, MMP9, PECAM1, PIK3CG, PIK3R1, PRKCA, PTPN11, ROCK1, VCAM1
IL-6 Signaling	7.39E+00	1.155	CD14, CHUK, IL18, IL1B, JAK2, NFKBIA, NRAS, PIK3CG, PIK3R1, PTPN11, RELB, STAT3, TNFRSF1A, TNFRSF1B
GADD45 Signaling	7.16E+00	0	BRCA1, CCNB1, CCND3, CCNE1, CDKN1A, IL1B, MYC, RELB, TGFB1, TGFBR1
LXR/RXR Activation	7.09E+00	-1.732	APOE, CD14, CD36, IL18, IL1B, IRF3, LY96, MMP9, RELB, S100A8, TLR4, TNFRSF1A, TNFRSF1B
Death Receptor Signaling	7.05E+00	0.302	CASP8, CHUK, FASLG, MAP3K5, NFKBIA, RELB, RIPK1, ROCK1, TNFRSF1A, TNFRSF1B, TNFSF10, TNKS
Role of JAK1, JAK2 and TYK2 in Interferon Signaling	7.04E+00	NA	IFNAR1, IFNGR1, JAK1, JAK2, RELB, STAT2, STAT3
MYC Mediated Apoptosis Signaling	6.90E+00	-1	BAX, CASP8, CDKN2A, CHUK, FASLG, MYC, PRKACB, TNFRSF1A, TNFRSF1B
Insulin Receptor Signaling	6.84E+00	-0.277	BAD, EIF2B4, EIF4EBP1, FYN, JAK1, JAK2, NRAS, PIK3CG, PIK3R1, PRKACB, PTEN, PTPN11, RPTOR, SGK1
Role of NANOG in Mammalian Embryonic Stem Cell Pluripotency	6.82E+00	0.707	AXIN1, CTNNB1, FZD9, JAK1, JAK2, JAK3, NRAS, PIK3CG, PIK3R1, SMAD5, STAT3, WNT10A, WNT5A
Virus Entry via Endocytic Pathways	6.80E+00	NA	B2M, FLNB, FYN, HLA-A, HLA-E, HLA-G, ITGB3, ITGB8, NRAS, PIK3CG, PIK3R1, PRKCA
Estrogen Receptor Signaling	6.76E+00	0	BAD, CDKN1A, EIF2B4, EIF4EBP1, ESR1, JAK1, JAK2, JAK3, MMP12, MMP13, MMP9, MYC, NOTCH1, NRAS, PIK3CG, PIK3R1, PRKAA2, PRKACB, PRKCA, PTEN, RELB, ROCK1, SDHA, VEGFB
Cell Cycle: G1/S Checkpoint Regulation	6.75E+00	-0.333	CCND3, CCNE1, CDK6, CDKN1A, CDKN2A, E2F3, HDAC4, MYC, RB1, TGFB1
JAK/STAT Signaling	6.74E+00	0.632	CDKN1A, JAK1, JAK2, JAK3, NRAS, PIK3CG, PIK3R1, PTPN11, RELB, STAT2, STAT3
IL-10 Signaling	6.50E+00	NA	CD14, CHUK, FCGR2B, IL10, IL18, IL1B, JAK1, NFKBIA, RELB, STAT3
Role of NFAT in Regulation of the Immune Response	6.47E+00	-1.091	CD247, CD4, CD79A, CD79B, CD80, CD86, CHUK, FCGR2B, FCGR3A/FCGR3B, FYN, HLA-A, HLA-DMA, HLA-DMB, HLA-DQA1, HLA-DQB1, HLA-DRB5, NFATC2, NFKBIA, NRAS, PIK3CG, PIK3R1, RELB, ZAP70
Regulation of Cellular Mechanics by Calpain Protease	6.47E+00	NA	CCND3, CCNE1, CDK6, ITGA4, ITGA6, ITGAL, ITGAM, ITGB3, ITGB8, NRAS, RB1
Sertoli Cell-Sertoli Cell Junction Signaling	6.31E+00	NA	AXIN1, CDH1, CTNNB1, ITGA4, ITGA6, ITGAL, ITGAM, ITGB3, ITGB8, MAP3K12, MAP3K5, NECTIN1, NRAS, PRKACB, PTEN, TNFRSF1A
Inhibition of ARE-Mediated mRNA Degradation Pathway	6.29E+00	-1	FASLG, LTB, PRKACB, PSMB10, PSMB5, PSMB8, PSMB9, PSMC4, TNFRSF1A, TNFRSF1B, TNFSF10, TNFSF13, TNFSF18, TNFSF8
Inflammasome pathway	6.21E+00	-1.633	CASP1, CASP8, IL18, IL1B, NOD2, TLR4
p53 Signaling	6.12E+00	-1.508	BAX, BIRC5, BRCA1, CDKN1A, CDKN2A, CTNNB1, PIK3CG, PIK3R1, PTEN, RB1, THBS1
MSP-RON Signaling In Cancer Cells Pathway	6.10E+00	-1.732	CSF2RB, CTNNB1, ITGA6, MET, MYC, NFKBIA, NRAS, PIK3CG, PIK3R1, RELB, STAT3, VEGFB, VHL
CD28 Signaling in T Helper Cells	6.09E+00	1.508	CD247, CD4, CD80, CD86, CHUK, CTLA4, FYN, HLA-A, HLA-DMA, HLA-DMB, HLA-DQA1, HLA-DQB1, HLA-DRB5, NFATC2, NFKBIA, PIK3CG, PIK3R1, PTPN11, RELB, ZAP70
IL-23 Signaling Pathway	5.93E+00	0.378	IL17A, IL1B, JAK2, NFKBIA, PIK3CG, PIK3R1, RELB, STAT3
TEC Kinase Signaling	5.91E+00	0.277	BLK, CD247, FASLG, FYN, HCK, ITGA4, ITGA6, ITGAL, ITGAM, ITGB3, ITGB8, JAK1, JAK2, JAK3, PIK3CG, PIK3R1, PRKCA, RELB, STAT2, STAT3, TLR4, TNFSF10
Sirtuin Signaling Pathway	5.88E+00	-1.807	BAX, CDH1, GLS, GLUD1, GOT2, LDHA, LDHB, MYC, NBN, PCK2, PDK1, PFKFB3, PRKAA2, RELB, RPTOR, SDHA, SLC2A1, STAT3
Sperm Motility	5.84E+00	NA	AXL, BLK, CSF1R, ERBB2, FYN, HCK, JAK1, JAK2, JAK3, KDR, MET, PDGFRB, PRKACB, PRKCA, TIE1, TWF1, ZAP70
Cyclins and Cell Cycle Regulation	5.84E+00	-0.333	CCNB1, CCND3, CCNE1, CDK6, CDKN1A, CDKN2A, E2F3, HDAC4, RB1, TGFB1
PKCθ Signaling in T Lymphocytes	5.83E+00	-0.894	CD247, CD4, CD80, CD86, CHUK, FYN, HLA-A, HLA-DMA, HLA-DMB, HLA-DQA1, HLA-DQB1, HLA-DRB5, MAP3K12, MAP3K5, NFATC2, NFKBIA, NRAS, PIK3CG, PIK3R1, RELB, ZAP70
LPS-stimulated MAPK Signaling	5.74E+00	0.707	CD14, CHUK, MAP3K5, NFKBIA, NRAS, PIK3CG, PIK3R1, PRKCA, RELB, TLR4

Role of RIG1-like Receptors in Antiviral Innate Immunity	5.68E+00	1.342	CASP8, CHUK, IRF3, IRF7, NFKBIA, RELB, RIPK1
Aryl Hydrocarbon Receptor Signaling	5.68E+00	0	BAX, CCND3, CCNE1, CDK6, CDKN1A, CDKN2A, ESR1, FASLG, IL1B, MYC, RB1, RELB, TGFB1
RAR Activation	5.62E+00	NA	ARID1A, CRABP2, CSF2RB, DUSP1, ERCC3, JAK2, MAP3K5, PIK3CG, PIK3R1, PRKACB, PRKCA, PTEN, RELB, SMAD5, TGFB1
PDGF Signaling	5.59E+00	0	JAK1, JAK2, JAK3, MYC, NRAS, PDGFRB, PIK3CG, PIK3R1, PRKCA, STAT3
DNA Double-Strand Break Repair by Homologous Recombination	5.55E+00	NA	BRCA1, BRCA2, MRE11, NBN, RAD51
Role of Cytokines in Mediating Communication between Immune Cells	5.51E+00	NA	IL10, IL15, IL17A, IL18, IL1B, IL24, TGFB1
Estrogen-mediated S-phase Entry	5.33E+00	-0.816	CCNE1, CDKN1A, E2F3, ESR1, MYC, RB1
MSP-RON Signaling Pathway	5.32E+00	NA	CCR2, CSF2RB, ITGAM, JAK2, PIK3CG, PIK3R1, TLR2, TLR4
Macropinocytosis Signaling	5.20E+00	0	CD14, CSF1R, ITGB3, ITGB8, MET, NRAS, PIK3CG, PIK3R1, PRKCA
VEGF Signaling	5.16E+00	-0.333	BAD, EIF2B4, KDR, NRAS, PIK3CG, PIK3R1, PRKCA, PTPN11, ROCK1, VEGFB
ATM Signaling	5.12E+00	-1.134	BLM, BRCA1, CCNB1, CDC25C, CDKN1A, MRE11, NBN, NFKBIA, RAD51, TLK2
IL-3 Signaling	5.06E+00	1	BAD, CSF2RB, JAK1, JAK2, NRAS, PIK3CG, PIK3R1, PRKCA, STAT3
CLEAR Signaling Pathway	5.00E+00	1.213	BNIP3, GUSB, KDR, NRAS, PDGFRB, PRKAA2, PRKCA, RPTOR, TGFB1, TGFBR1, TLR1, TLR2, TLR4, TLR7, TLR9, TNFRSF1A, TNFRSF1B
Thrombopoietin Signaling	4.87E+00	0	JAK2, MYC, NRAS, PIK3CG, PIK3R1, PRKCA, PTPN11, STAT3
PEDF Signaling	4.84E+00	-1.134	CASP8, CHUK, FASLG, NFKBIA, NRAS, PIK3CG, PIK3R1, RELB, ROCK1
Prolactin Signaling	4.75E+00	0.333	FYN, JAK2, MYC, NRAS, PIK3CG, PIK3R1, PRKCA, PTPN11, STAT3
Regulation Of The Epithelial Mesenchymal Transition In Development Pathway	4.75E+00	1.414	AXIN1, BRCA1, CDH1, CTNNB1, FZD9, NOTCH1, RELB, WNT10A, WNT5A
Gα12/13 Signaling	4.74E+00	1	CDH1, CDH5, CHUK, CTNNB1, MAP3K5, NFKBIA, NRAS, PIK3CG, PIK3R1, RELB, ROCK1
CD40 Signaling	4.72E+00	0.378	CD40, CHUK, JAK3, NFKBIA, PIK3CG, PIK3R1, RELB, STAT3
PPARα/RXRα Activation	4.60E+00	-1.508	CD36, CHUK, GOT2, IL1B, JAK2, NFKBIA, NRAS, PRKAA2, PRKACB, PRKCA, RELB, TGFB1, TGFBR1
RAC Signaling	4.56E+00	-1.342	CYBB, ITGA4, ITGA6, ITGAL, ITGAM, ITGB3, ITGB8, NRAS, PIK3CG, PIK3R1, RELB
IL-9 Signaling	4.54E+00	0.447	JAK1, JAK3, PIK3CG, PIK3R1, RELB, STAT3
Germ Cell-Sertoli Cell Junction Signaling	4.54E+00	NA	AXIN1, CDH1, CTNNB1, ITGA6, MAP3K12, MAP3K5, NRAS, PIK3CG, PIK3R1, TGFB1, TGFBR1, TNFRSF1A
GM-CSF Signaling	4.53E+00	-0.707	CSF2RB, HCK, JAK2, NRAS, PIK3CG, PIK3R1, PTPN11, STAT3
Communication between Innate and Adaptive Immune Cells	4.52E+00	NA	B2M, Ccl9, CD247, CD4, CD40, CD79A, CD79B, CD80, CD86, HLA-A, HLA-DRB5, HLA-E, HLA-G, IL10, IL15, IL18, IL1B, TLR1, TLR2, TLR4, TLR7, TLR9, TNFRSF17, TNFSF13
ILK Signaling	4.50E+00	-0.905	CDH1, CTNNB1, FLNB, ITGB3, ITGB8, MMP9, MYC, PIK3CG, PIK3R1, PTEN, RELB, TNFRSF1A, VEGFB
PAK Signaling	4.50E+00	-1.342	ITGA4, ITGA6, ITGAL, ITGAM, ITGB3, ITGB8, NRAS, PDGFRB, PIK3CG, PIK3R1
p38 MAPK Signaling	4.50E+00	0	DUSP1, FASLG, IL18, IL1B, MAP3K5, MYC, TGFB1, TGFBR1, TNFRSF1A, TNFRSF1B
Lymphotoxin β Receptor Signaling	4.39E+00	0	CHUK, LTB, NFKBIA, PIK3CG, PIK3R1, RELB, VCAM1
Role of CHK Proteins in Cell Cycle Checkpoint Control	4.33E+00	-1.134	BRCA1, CDC25C, CDKN1A, E2F3, MRE11, NBN, TLK2
Protein Kinase A Signaling	4.28E+00	2.324	BAD, CDC25C, CHUK, CTNNB1, DUSP1, DUSP2, DUSP5, FLNB, NFATC2, NFKBIA, PRKACB, PRKCA, PTEN, PTPN11, RELB, ROCK1, SIRPA, TGFB1, TGFBR1
G-Protein Coupled Receptor Signaling	4.27E+00	0.192	ADGRE1, ADORA2A, APLNR, C5AR1, CCR2, CCR9, CHUK, CMKLR1, CTNNB1, CX3CR1, DUSP1, FYN, FZD9, MAP3K12, MAP3K5, NFATC2, NFKBIA, NRAS, P2RY13, PIK3CG, PIK3R1, PRKACB, PRKCA, PTGER4, RELB, ROCK1, STAT3
Insulin Secretion Signaling Pathway	4.19E+00	1.807	EIF2B4, EIF4EBP1, FYN, HCK, JAK1, JAK2, JAK3, PC, PIK3CG, PIK3R1, PRKACB, PRKCA, SLC2A1, STAT2, STAT3

Protein Ubiquitination Pathway	4.17E+00	NA	B2M, BRCA1, CDC20, HLA-A, HLA-E, HLA-G, PSMB10, PSMB5, PSMB8, PSMB9, PSMC4, TAP1, TAP2, UBE2C, VHL
NUR77 Signaling in T Lymphocytes	4.13E+00	-0.378	B2M, CD247, CD80, CD86, FASLG, HLA-A, HLA-DMA, HLA-DMB, HLA-DQA1, HLA-DQB1, HLA-DRB5, HLA-E, HLA-G, PDK1, PRKCA, TNFSF10
IGF-1 Signaling	4.12E+00	1	BAD, JAK1, JAK2, NRAS, PIK3CG, PIK3R1, PRKACB, PTPN11, STAT3
Epithelial Adherens Junction Signaling	4.12E+00	0.905	CDH1, CTNNB1, FYN, MET, NECTIN1, NOTCH1, NRAS, PRKAA2, PTEN, ROCK1, TGFBR1
Oncostatin M Signaling	4.07E+00	0	JAK1, JAK2, JAK3, MMP13, NRAS, STAT3
April Mediated Signaling	4.07E+00	-0.447	CHUK, NFATC2, NFKBIA, RELB, TNFRSF17, TNFSF13
FLT3 Signaling in Hematopoietic Progenitor Cells	4.07E+00	0.707	BAD, EIF4EBP1, NRAS, PIK3CG, PIK3R1, PTPN11, STAT2, STAT3
BAG2 Signaling Pathway	4.07E+00	NA	CDKN1A, MYC, PSMB10, PSMB5, PSMB8, PSMB9, PSMC4, RELB
IL-2 Signaling	4.04E+00	-0.378	IL2RB, JAK1, JAK3, NRAS, PIK3CG, PIK3R1, PTPN11
Paxillin Signaling	4.02E+00	-1	ITGA4, ITGA6, ITGAL, ITGAM, ITGB3, ITGB8, NRAS, PIK3CG, PIK3R1
PPAR Signaling	4.02E+00	-0.707	CHUK, IL18, IL1B, NFKBIA, NRAS, PDGFRB, RELB, TNFRSF1A, TNFRSF1B
Adipogenesis pathway	3.98E+00	NA	CTNNB1, ERCC3, EZH2, FZD9, HDAC4, RB1, SMAD5, TGFB1, TNFRSF1A, WNT5A
Apelin Pancreas Signaling Pathway	3.90E+00	-1.342	APLNR, PIK3CG, PIK3R1, PRKAA2, PRKACB, RELB
FGF Signaling	3.89E+00	-0.707	FGF9, MAP3K5, MET, PIK3CG, PIK3R1, PRKCA, PTPN11, STAT3
Human Embryonic Stem Cell Pluripotency	3.84E+00	NA	AXIN1, CTNNB1, FZD9, PDGFRB, PIK3CG, PIK3R1, SMAD5, TGFB1, TGFBR1, WNT10A, WNT5A
Actin Nucleation by ARP-WASP Complex	3.82E+00	NA	ITGA4, ITGA6, ITGAL, ITGAM, ITGB3, ITGB8, NRAS, ROCK1
WNT/β-catenin Signaling	3.73E+00	-0.707	AXIN1, CDH1, CDH5, CDKN2A, CTNNB1, FZD9, MYC, TGFB1, TGFBR1, WNT10A, WNT5A
NAD Signaling Pathway	3.69E+00	-0.333	CD38, LDHA, LDHB, NT5E, PIK3CG, PIK3R1, POLR2A, PRKAA2, TGFB1, TNKS
Regulation of eIF4 and p70S6K Signaling	3.68E+00	-1	EIF2B4, EIF4EBP1, ITGA4, ITGA6, ITGAL, ITGAM, ITGB3, ITGB8, NRAS, PIK3CG, PIK3R1
Signaling by Rho Family GTPases	3.68E+00	-1.414	CDH1, CDH5, CYBB, ITGA4, ITGA6, ITGAL, ITGAM, ITGB3, ITGB8, MAP3K12, PIK3CG, PIK3R1, RELB, ROCK1
Role of JAK2 in Hormone-like Cytokine Signaling	3.66E+00	NA	JAK1, JAK2, PTPN11, SIRPA, STAT3
TNFR1 Signaling	3.64E+00	1.342	CASP8, CHUK, NFKBIA, RELB, RIPK1, TNFRSF1A
4-1BB Signaling in T Lymphocytes	3.60E+00	NA	CHUK, MAP3K5, NFKBIA, RELB, TNFRSF9
Complement System	3.60E+00	1	C1QA, C1QB, C2, C5AR1, ITGAM
CSDE1 Signaling Pathway	3.60E+00	0	CCND3, CTNNB1, MYC, PTEN, PUM1, TGFB1
Integrin Signaling	3.58E+00	-0.577	FYN, ITGA4, ITGA6, ITGAL, ITGAM, ITGB3, ITGB8, NRAS, PIK3CG, PIK3R1, PTEN, ROCK1
OX40 Signaling Pathway	3.55E+00	0	B2M, CD247, CD4, HLA-A, HLA-DMA, HLA-DMB, HLA-DQA1, HLA-DQB1, HLA-DRB5, HLA-E, HLA-G, NFKBIA, RELB, TNFRSF4
MIF-mediated Glucocorticoid Regulation	3.54E+00	0	CD14, LY96, NFKBIA, RELB, TLR4
CD27 Signaling in Lymphocytes	3.37E+00	0	CASP8, CHUK, MAP3K12, MAP3K5, NFKBIA, RELB
TWEAK Signaling	3.36E+00	0	CASP8, CHUK, NFKBIA, RELB, RIPK1
Telomerase Signaling	3.34E+00	-0.447	CDKN1A, HDAC4, IL2RB, MYC, NRAS, PIK3CG, PIK3R1, RB1
p70S6K Signaling	3.33E+00	0.333	BAD, CD19, CD79A, CD79B, JAK1, NRAS, PIK3CG, PIK3R1, PRKCA
CTLA4 Signaling in Cytotoxic T Lymphocytes	3.30E+00	NA	B2M, CD247, CD80, CD86, CTLA4, FYN, HLA-A, HLA-E, HLA-G, JAK2, PIK3CG, PIK3R1, PTPN11, ZAP70
Tumoricidal Function of Hepatic Natural Killer Cells	3.28E+00	NA	BAX, CASP8, FASLG, ITGAL
CDC42 Signaling	3.23E+00	NA	B2M, CD247, HLA-A, HLA-DMA, HLA-DMB, HLA-DQA1, HLA-DQB1, HLA-DRB5, HLA-E, HLA-G, ITGA4, ITGA6, ITGAL, ITGAM, ITGB3, ITGB8
MIF Regulation of Innate Immunity	3.10E+00	0	CD14, LY96, NFKBIA, RELB, TLR4
Role of JAK family kinases in IL-6-type Cytokine Signaling	3.06E+00	NA	JAK1, JAK2, PTPN11, STAT3

Glycolysis I	3.06E+00	0	ALDOA, ALDOC, ENO1, PKM
B Cell Activating Factor Signaling	3.05E+00	NA	CHUK, NFATC2, NFKBIA, RELB, TNFRSF17
Ceramide Signaling	3.05E+00	0	BAD, NRAS, PIK3CG, PIK3R1, RELB, TNFRSF1A, TNFRSF1B
Glutamate Degradation II	3.04E+00	NA	GOT1, GOT2
Aspartate Biosynthesis	3.04E+00	NA	GOT1, GOT2
Actin Cytoskeleton Signaling	3.02E+00	-0.447	CD14, FGF9, ITGA4, ITGA6, ITGAL, ITGAM, ITGB3, ITGB8, NRAS, PIK3CG, PIK3R1, ROCK1
Retinoic acid Mediated Apoptosis Signaling	3.01E+00	0	CASP8, CRABP2, IFNAR1, TNFSF10, TNKS

*Z-score: positive score = activated pathway; negative score = inhibited pathway; NA = no activity pattern available.

Supplemental Table 3. Modulated canonical pathways in the peripheral blood microenvironment (PBMCs) following BET inhibitor treatment of leukemic mice (adoptive transfer Eμ-TCL1) identified by IPA using NanoString PanCancer iO360 expression panel ($p < 0.001$).

Ingenuity Canonical Pathways	-Log (p-value)	Z-score*	Molecules
Pathogen Induced Cytokine Storm Signaling Pathway	3.74E+01	-4.919	ATP,CASP1,CASP8,CCL1,CCL11,CCL20,CCR5,CD70,CSF2RB,Cxcl3,FASLG,HLA-DMA,HLA-DMB,HLA-DOB,HLA-DQA1,HLA-DQB1,HLA-DRB5,IFIH1,IFNGR2,IL15,IL18,IL1RN,IL21R,IL6R,IRF9,JAK1,LTB,MAPK10,MYC,MYD88,NFKB1,PRDM1,RIPK3,SLC2A1,TBX21,TGFB1,TICAM1,TLR1,TLR2,TLR4,TLR7,TLR8,TLR9,TNF,VEGFB
Neuroinflammation Signaling Pathway	3.58E+01	-3.795	ATP,B2M,BCL2,CASP1,CASP8,CD200R1,CD80,CD86,CTNNB1,CYBB,FASLG,GLS,HLA-DMA,HLA-DMB,HLA-DOB,HLA-DQA1,HLA-DQB1,HLA-DRB5,HLA-E,HLA-G,IFNGR2,IL18,IL6R,JAK1,JAK3,MAPK10,MYD88,NFKB1,PIK3CD,PIK3CG,PIK3R1,TGFB1,TGFBR1,TGFBR2,TICAM1,TLR1,TLR2,TLR4,TLR7,TLR8,TLR9,TNF
Macrophage Classical Activation Signaling Pathway	2.94E+01	-4.131	CCL20,CD70,CD80,CD86,CYBB,FASLG,GBP4,HLA-DMA,HLA-DMB,HLA-DOB,HLA-DQA1,HLA-DQB1,HLA-DRB5,IFNGR2,IL15,IL18,IRF5,IRF8,IRF9,JAK1,LTB,LY96,MYD88,NFKB1,NFKBIA,PARP9,TGFB1,TICAM1,TLR4,TLR9,TNF
Macrophage Alternative Activation Signaling Pathway	2.93E+01	-3.413	ADORA2A,CD36,CSF1,EIF4EBP1,FCGR2B,HLA-DMA,HLA-DMB,HLA-DOB,HLA-DQA1,HLA-DQB1,HLA-DRB5,IL18,IL1RN,IL2RG,IRF4,IRF5,JAK1,JAK3,MRC1,MYC,NFKB1,NFKBIA,PIK3CD,PIK3CG,PIK3R1,RPS6KB1,RPTOR,SREBF1,TGFB1,TLR4,TNF
Th1 and Th2 Activation Pathway	2.83E+01	NaN	CCR5,CD274,CD3G,CD80,CD86,HLA-DMA,HLA-DMB,HLA-DOB,HLA-DQA1,HLA-DQB1,HLA-DRB5,ICOS,IFNGR2,IKZF1,IL18,IL2RG,IL6R,JAG1,JAK1,JAK3,NFIL3,NFKB1,PIK3CD,PIK3CG,PIK3R1,TBX21,TGFB1,TGFBR1,TGFBR2
Multiple Sclerosis Signaling Pathway	2.58E+01	-3.286	ATP,CASP1,CASP8,CD70,CTLA4,FASLG,HLA-DMA,HLA-DMB,HLA-DOB,HLA-DQA1,HLA-DQB1,HLA-DRB5,HLA-E,HLA-G,IFNGR2,IL15,IL18,IRF4,LTB,NFKB1,PARP4,PARP9,TGFB1,TLR1,TLR2,TLR4,TLR7,TLR8,TLR9,TNF
ISGylation Signaling Pathway	2.48E+01	-3.838	ATP,DTX3L,EIF2AK2,FLNB,IFIH1,IRF2,IRF4,IRF5,IRF8,IRF9,ITGA2,ITGAL,JAK1,MAPK10,NFKB1,TLR1,TLR2,TLR4,TLR7,TLR8,TLR9,TP53,UBA7
Colorectal Cancer Metastasis Signaling	2.47E+01	-2.6	ATP,AXIN1,CASP9,CTNNB1,GNG4,IL6R,JAK1,JAK3,MAPK10,MLH1,MYC,NFKB1,NRAS,PIK3CD,PIK3CG,PIK3R1,TCF3,TGFB1,TGFBR1,TGFBR2,TLR1,TLR2,TLR4,TLR7,TLR8,TLR9,TNF,TP53,VEGFB,WNT10A,WNT2B
IL-27 Signaling Pathway	2.43E+01	-3.674	ATP,B2M,CASP1,CD274,ENTPD1,HLA-E,HLA-G,IFNGR2,IRF8,JAK1,JAK3,MYD88,NFKB1,PIK3CD,PIK3CG,PIK3R1,TBX21,TLR1,TLR2,TLR4,TLR7,TLR8,TLR9,TNFRSF9
NAFLD Signaling Pathway	2.40E+01	-2.785	ATP,CASP1,CASP8,CD36,CD70,FASLG,IL15,IL18,IL6R,JAK1,JAK3,LTB,MAP3K5,MAPK10,MYD88,NFKB1,PIK3CD,PIK3CG,PIK3R1,RPTOR,SREBF1,TGFB1,TLR1,TLR2,TLR4,TLR7,TLR8,TLR9,TNF
Th2 Pathway	2.39E+01	-2.858	CCR5,CD3G,CD80,CD86,HLA-DMA,HLA-DMB,HLA-DOB,HLA-DQA1,HLA-DQB1,HLA-DRB5,ICOS,IKZF1,IL2RG,JAG1,JAK1,JAK3,NFKB1,PIK3CD,PIK3CG,PIK3R1,TBX21,TGFB1,TGFBR1,TGFBR2
Role of Pattern Recognition Receptors in Recognition of Bacteria and Viruses	2.39E+01	-3.153	CASP1,CD70,EIF2AK2,FASLG,IFIH1,IL15,IL18,LTB,MAPK10,MYD88,NFKB1,OAS2,PIK3CD,PIK3CG,PIK3R1,PRKCA,TGFB1,TICAM1,TLR1,TLR2,TLR4,TLR7,TLR8,TLR9,TNF
Th1 Pathway	2.36E+01	-3.962	CCR5,CD274,CD3G,CD80,CD86,HLA-DMA,HLA-DMB,HLA-DOB,HLA-DQA1,HLA-DQB1,HLA-DRB5,ICOS,IFNGR2,IL18,IL6R,JAK1,JAK3,NFIL3,NFKB1,PIK3CD,PIK3CG,PIK3R1,TBX21
Molecular Mechanisms of Cancer	2.36E+01	-1.769	ADORA2A,ATP,AXIN1,BCL2,CASP8,CASP9,CCND2,CCND3,CCR2,CCR5,CCR9,CSF2RB,CTNNB1,E2F3,FASLG,FYN,GNG4,HDAC3,HES1,IHH,IL21R,IL2RG,IL6R,ITGA2,ITGA4,ITGAL,JAK1,JAK3,MAP3K5,MAPK10,MDM2,MRE11,MYC,NF1,NFKB1,NFKBIA,NRAS,PIK3CD,PIK3CG,PIK3R1,PRKCA,RBL2,TCF3,TGFB1,TGFBR1,TGFBR2,TP53
PD-1, PD-L1 cancer immunotherapy pathway	2.35E+01	3.13	B2M,CD274,CD80,HLA-DMA,HLA-DMB,HLA-DOB,HLA-DQA1,HLA-DQB1,HLA-DRB5,HLA-E,HLA-

			G,IFNGR2,IL2RG,JAK1,JAK3,LCK,PDCD1LG2,PIK3CD,PIK3CG,PIK3R1,TGFB1,TNF
Glucocorticoid Receptor Signaling	2.25E+01	NaN	ARID1A,B2M,BCL2,CCL11,CD3G,CSF2RB,ESR1,HLA-DMA,HLA-DMB,HLA-DOB,HLA-DQA1,HLA-DQB1,HLA-DRB5,HLA-E,HLA-G,IL1RN,IL21R,IL2RG,IL6R,JAK1,JAK3,KAT2B,MAPK10,MYC,NFKB1,NFKBIA,NRAS,PC,PCK2,PIK3CD,PIK3CG,PIK3R1,POLR2A,TBP,TGFB1,TGFBR1,TGFBR2,TLR2,TNF
IL-10 Signaling	2.22E+01	1.633	BCL2,CCR5,CD80,CD86,FCGR2B,HLA-DMA,HLA-DMB,HLA-DOB,HLA-DQA1,HLA-DQB1,HLA-DRB5,HLA-E,HLA-G,IFNGR2,IHH,IL18,IL1RN,IL6R,JAK1,NFKB1,NFKBIA,PRDM1,TLR4,TNF
Role of Macrophages, Fibroblasts and Endothelial Cells in Rheumatoid Arthritis	2.20E+01	NaN	AXIN1,CSF1,CTNNB1,IL15,IL16,IL18,IL1RN,IL6R,LTB,MYC,MYD88,NFKB1,NFKBIA,NRAS,PIK3CD,PIK3CG,PIK3R1,PRKCA,TCF3,TGFB1,TLR1,TLR2,TLR4,TLR7,TLR8,TLR9,TNF,TRAF1,VEGFB,WNT10A,WNT2B
Cachexia Signaling Pathway	2.16E+01	-1.976	ATP,BCL2,CASP1,CASP8,CASP9,CD70,EIF2AK2,EIF2B4,FASLG,IFNGR2,IL15,IL18,IL1RN,IL6R,LTB,MAPK10,MYD88,NFKB1,PIK3CD,PIK3CG,PIK3R1,PRKCA,PSMB10,PSMB8,PSMC4,TGFB1,TGFBR1,TGFBR2,TLR4,TLR7,TLR8,TNF
Cardiac Hypertrophy Signaling (Enhanced)	2.15E+01	-2.921	ATP,CD70,CSF2RB,CTNNB1,CYBB,EIF2B4,EIF4EBP1,FASLG,GNG4,HDAC3,IL15,IL18,IL21R,IL2RG,IL6R,ITGA2,ITGA4,ITGAL,LTB,MAP3K12,MAP3K5,MAP3K8,MAPK10,MYC,NFKB1,NRAS,PIK3CD,PIK3CG,PIK3R1,PRKCA,RPS6KB1,TGFB1,TGFBR1,TGFBR2,TNF,WNT10A,WNT2B
Chronic Myeloid Leukemia Signaling	1.98E+01	-0.784	AXIN1,BCL2,BLK,CASP9,CCND2,CCND3,CTNNB1,E2F3,HDAC3,HES1,IRF8,MDM2,MYC,NFKB1,NRAS,PIK3CD,PIK3CG,PIK3R1,RBL2,RPTOR,TCF3,TGFB1,TGFBR1,TGFBR2,TP53,WNT10A,WNT2B
Crosstalk between Dendritic Cells and Natural Killer Cells	1.90E+01	-3.638	CD80,CD86,CSF2RB,FASLG,HLA-DRB5,HLA-E,HLA-G,IL15,IL18,IL2RG,ITGAL,LTB,MICB,NFKB1,TLR4,TLR7,TLR9,TNF
PI3K/AKT Signaling	1.84E+01	-1.698	BCL2,CSF2RB,CTNNB1,EIF4EBP1,IL21R,IL2RG,IL6R,ITGA2,ITGA4,ITGAL,JAK1,JAK3,MAP3K5,MAP3K8,MDM2,NFKB1,NFKBIA,NRAS,PIK3CD,PIK3CG,PIK3R1,RPS6KB1,TP53
Hepatic Fibrosis Signaling Pathway	1.79E+01	-2.785	AXIN1,BCL2,CTNNB1,CYBB,IL18,IL1RN,ITGA2,ITGA4,ITGAL,JAK1,MAPK10,MYC,MYD88,NFKB1,NFKBIA,NRAS,PIK3CD,PIK3CG,PIK3R1,PRKCA,RPS6KB1,TCF3,TGFB1,TGFBR1,TGFBR2,TLR4,TNF,VEGFB,WNT10A,WNT2B
Systemic Lupus Erythematosus in B Cell Signaling Pathway	1.75E+01	-2.137	BCL2,CCND2,CCND3,CD19,CD5,CD70,CD79B,CTNNB1,FASLG,FCGR2B,FYN,IFIH1,IFNGR2,IL15,IL18,IL6R,IRF5,IRF9,JAK1,LCK,LILRB3,LTB,MYC,MYD88,NFKB1,NRAS,PIK3CD,PIK3CG,PIK3R1,PRKCA,TGFB1,TICAM1,TLR7,TLR8,TLR9,TNF,TRAF1
Natural Killer Cell Signaling	1.73E+01	-2.985	B2M,CD48,FASLG,FYN,HLA-E,HLA-G,IL15,IL18,ITGAL,JAK3,LCK,MAP3K12,MAP3K5,MAP3K8,MICB,MYD88,NFKB1,NRAS,PIK3CD,PIK3CG,PIK3R1,ULBP1
Coronavirus Pathogenesis Pathway	1.72E+01	-0.426	BCL2,CASP1,CASP8,CASP9,CCR2,E2F3,FASLG,HDAC3,IRF9,JAK1,MAPK10,NFKB1,NFKBIA,OAS2,RBL2,RIPK3,TGFB1,TGFBR1,TGFBR2,TICAM1,TLR7,TP53
Antigen Presentation Pathway	1.72E+01	NaN	B2M,CD74,HLA-DMA,HLA-DMB,HLA-DOB,HLA-DQA1,HLA-DQB1,HLA-DRB5,HLA-E,HLA-G,PSMB8,TAP2,TAPBP
Toll-like Receptor Signaling	1.72E+01	-2.714	EIF2AK2,IL18,IL1RN,LY96,MYD88,NFKB1,NFKBIA,TICAM1,TLR1,TLR2,TLR4,TLR7,TLR8,TLR9,TNF,TRAF1
Interferon gamma signaling	1.71E+01	-3.638	ATP,B2M,GBP3,GBP4,HLA-DQA1,HLA-DQB1,HLA-DRB5,HLA-E,HLA-G,IFNGR2,IRF2,IRF4,IRF5,IRF8,IRF9,JAK1,OAS2
NOD1/2 Signaling Pathway	1.67E+01	-2.837	CASP1,CD70,FASLG,IL15,IL18,IRF5,LTB,MAP3K8,MAPK10,MYD88,NFKB1,NFKBIA,TGFB1,TLR1,TLR2,TLR4,TLR7,TLR8,TLR9,TNF,TNFSF18
IL-12 Signaling and Production in Macrophages	1.65E+01	-1.46	CCR5,CD47,FCGR2B,HDAC3,IFIH1,IL18,IRF5,IRF8,LCK,MAP3K8,MYD88,NFIL3,NFKB1,PIK3CD,PIK3CG,PIK3R1,PRKCA,TBX21,TGFB1,TLR2,TLR4,TLR9,TNF
Costimulation by the CD28 family	1.65E+01	-3.5	ATP,CD274,CD3G,CD80,CD86,CTLA4,FYN,HLA-DQA1,HLA-DQB1,HLA-DRB5,ICOS,LCK,MAP3K8,PDCD1LG2,PIK3R1,TNFRSF14
Autism Signaling Pathway	1.63E+01	-2.6	ATP,AXIN1,B2M,CD70,CTNNB1,EIF4EBP1,FASLG,HLA-E,HLA-G,IL15,IL18,IL1RN,LTB,NF1,NRAS,PIK3CD,PIK3CG,PIK3R1,RPS6KB1,RPTOR,TCF3,TGFB1,TNF,WNT10A,WNT2B
Pancreatic Adenocarcinoma Signaling	1.63E+01	-1.732	BCL2,CASP9,E2F3,HDAC3,JAK1,JAK3,MAPK10,MDM2,NFKB1,PIK3CD,PIK3CG,PIK3R1,RBL2,TGFB1,TGFBR1,TGFBR2,TP53,VEGFB
Pulmonary Healing Signaling Pathway	1.62E+01	-1.528	BLK,CTNNB1,FYN,JAG1,LCK,MAPK10,MYC,MYD88,NFKB1,NFKBIA,NRAS,PECAM1,PRKCA,TCF3,TGFB1,TLR2,TLR4,TNF,VEGFB,WNT10A,WNT2B

ID1 Signaling Pathway	1.61E+01	-1.964	BCL2,BLK,CTNNB1,EGR1,FYN,IL6R,LCK,MDM2,MYC,NFKB1,NRAS,PIK3CD,PIK3CG,PIK3R1,TGFB1,TGFBR1,TGFBR2,TNF,TP53,TYMS,VEGFB
Erythropoietin Signaling Pathway	1.59E+01	-0.894	CCND2,CD70,CSF2RB,CTNNB1,FASLG,IL15,IL18,LTB,MDM2,NFKB1,NFKBIA,NRAS,PIK3CD,PIK3CG,PIK3R1,PRKCA,RPS6KB1,TGFB1,TNF,TP53
Role of PKR in Interferon Induction and Antiviral Response	1.57E+01	-3.3	CASP1,CASP8,CASP9,EIF2AK2,FASLG,IFIH1,IFNGR2,IL18,IRF9,JAK1,MAPK10,MYD88,NFKB1,NFKBIA,TLR4,TLR9,TNF,TP53
Regulation of the Epithelial Mesenchymal Transition by Growth Factors Pathway	1.54E+01	-1.789	CD70,EGR1,FASLG,IL6R,JAK1,JAK3,LTB,MAPK10,NFKB1,NRAS,PIK3CD,PIK3CG,PIK3R1,TCF3,TGFB1,TGFBR1,TGFBR2,TNF,TNFSF18,ZEB2
Activin Inhibin Signaling Pathway	1.54E+01	-2.837	CCND2,CTNNB1,IL18,IL1RN,MAPK10,MDM2,MYD88,NFKB1,NFKBIA,PIK3CD,PIK3CG,PIK3R1,TCF3,TGFB1,TLR1,TLR2,TLR4,TLR7,TLR8,TLR9,TNF
Ribonucleotide Reductase Signaling Pathway	1.51E+01	-1.606	ARID1A,ATP,BCL2,CASP9,CTNNB1,E2F3,EIF4EBP1,ESR1,MAPK10,MYC,NFKB1,NRAS,PARP4,PARP9,PIK3CD,PIK3CG,PIK3R1,RPTOR,TP53
Role of Tissue Factor in Cancer	1.48E+01	-1.342	BCL2,BLK,CSF1,EGR1,FYN,LCK,MAPK10,NFKB1,NRAS,PIK3CD,PIK3CG,PIK3R1,PRKCA,RBL2,RPS6KB1,TGFB1,TGFBR1,TGFBR2,TNF,TP53
Immunoregulatory interactions between a Lymphoid and a non-Lymphoid cell	1.47E+01	-4.025	B2M,CD19,CD1D,CD200R1,CD3G,CD96,FCGR2B,HLA-E,HLA-G,ICAM2,ITGA2,ITGA4,ITGAL,KLRG1,LILRA5,LILRB3,MICB,SELL,SLAMF7,ULBP1
Interleukin-4 and Interleukin-13 signaling	1.46E+01	-1.291	ATP,BCL2,CCL11,CD36,FASLG,IL18,IL2RG,IL6R,IRF4,JAK1,JAK3,MYC,PIK3R1,TGFB1,TNF,TP53
T Cell Exhaustion Signaling Pathway	1.46E+01	-0.688	CD274,CD3G,CD80,CD86,CTLA4,HLA-DMA,HLA-DMB,HLA-DOB,HLA-DQA1,HLA-DQB1,HLA-DRB5,HLA-E,HLA-G,IL6R,IRF4,IRF9,JAK1,JAK3,MAPK10,NRAS,PDCD1LG2,PIK3CD,PIK3CG,PIK3R1,PRDM1,TBX21,TGFB1,TGFBR1,TGFBR2,TNFRSF14
Osteoarthritis Pathway	1.45E+01	-1.886	ATP,CASP1,CASP8,CASP9,CTNNB1,HDAC3,HES1,IHH,ITGA2,ITGA4,ITGAL,JAG1,NFKB1,TCF3,TGFB1,TGFBR1,TGFBR2,TLR2,TLR4,TNF,VEGFB
Ovarian Cancer Signaling	1.45E+01	-2.449	AXIN1,BCL2,CTNNB1,E2F3,HDAC3,MLH1,NRAS,PIK3CD,PIK3CG,PIK3R1,PMS2,RBL2,RPS6KB1,TCF3,TP53,VEGFB,WNT10A,WNT2B
Role of Osteoblasts in Rheumatoid Arthritis Signaling Pathway	1.44E+01	0.218	AXIN1,BCL2,CD70,CTNNB1,CTSS,FASLG,IL15,IL18,IL6R,JAK1,JAK3,LTB,PIK3CD,PIK3CG,PIK3R1,TCF3,TGFB1,TNF,VEGFB,WNT10A,WNT2B
Tumor Microenvironment Pathway	1.44E+01	-2.065	BCL2,CD274,CSF1,CTLA4,FASLG,HLA-E,HLA-G,IL6R,MYC,NFKB1,NRAS,PDCD1LG2,PIK3CD,PIK3CG,PIK3R1,SLC2A1,TGFB1,TNF,VEGFB
Pyroptosis Signaling Pathway	1.42E+01	-3.357	CASP1,CASP8,CASP9,GBP3,GBP4,IL18,IRF2,NFKB1,TLR1,TLR2,TLR4,TLR7,TLR8,TLR9,TNF
Small Cell Lung Cancer Signaling	1.42E+01	-1.89	BCL2,CASP9,CCND2,CCND3,E2F3,HDAC3,MYC,NFKB1,NFKBIA,PIK3CD,PIK3CG,PIK3R1,RBL2,TP53,TRAF1
Dendritic Cell Maturation	1.41E+01	-4.271	B2M,CD1D,CD3G,CD80,CD86,FCGR2B,HLA-DMA,HLA-DMB,HLA-DOB,HLA-DQA1,HLA-DQB1,HLA-DRB5,HLA-E,HLA-G,IL15,IL18,IL1RN,IRF8,LTB,MAPK10,MYD88,NFKB1,NFKBIA,PIK3CD,PIK3CG,PIK3R1,TLR2,TLR4,TLR9,TNF
Production of Nitric Oxide and Reactive Oxygen Species in Macrophages	1.40E+01	-2.524	CYBB,IFNGR2,IRF8,JAK1,JAK3,MAP3K12,MAP3K5,MAP3K8,MAPK10,NFKB1,NFKBIA,PIK3CD,PIK3CG,PIK3R1,PRKCA,SIRPA,TLR2,TLR4,TNF
CDX Gastrointestinal Cancer Signaling Pathway	1.37E+01	0.229	CD70,CTNNB1,CYBB,FASLG,IL15,IL18,IL6R,JAK1,JAK3,LTB,NFKB1,PIK3CD,PIK3CG,PIK3R1,TCF3,TGFB1,TNF,WNT10A,WNT2B
Role of Hypercytokinemia/hyperchemokinemia in the Pathogenesis of Influenza	1.36E+01	-2.673	CASP1,EIF2AK2,IL18,IL1RN,IRF9,JAK1,MYD88,NFKB1,OAS2,TICAM1,TLR4,TLR7,TLR9,TNF
Regulation of the Epithelial-Mesenchymal Transition Pathway	1.30E+01	NaN	AXIN1,CTNNB1,EGR1,JAG1,JAK1,JAK3,NFKB1,NRAS,PIK3CD,PIK3CG,PIK3R1,TCF3,TGFB1,TGFBR1,TGFBR2,WNT10A,WNT2B,ZEB2
Pulmonary Fibrosis Idiopathic Signaling Pathway	1.29E+01	-1.964	BCL2,CTNNB1,EGR1,EIF4EBP1,HES1,ITGA2,JAG1,MAPK10,NFKB1,NRAS,PIK3CD,PIK3CG,PIK3R1,POLR2A,RPS6KB1,TCF3,TGFB1,TGFBR1,TGFBR2,TP53,WNT10A,WNT2B
TREM1 Signaling	1.29E+01	-3.051	CASP1,CD86,FCGR2B,IL18,MYD88,NFKB1,TLR1,TLR2,TLR4,TLR7,TLR8,TLR9,TNF
T Cell Receptor Signaling	1.28E+01	-3.9	B2M,CD3G,CD80,CD86,CTLA4,CTNNB1,FYN,HLA-DMA,HLA-DMB,HLA-DOB,HLA-DQA1,HLA-DQB1,HLA-DRB5,HLA-E,HLA-G,ICOS,ITGA2,ITGAL,LCK,NFKB1,NFKBIA,NRAS,PIK3CD,PIK3CG,PIK3R1,PTPRC,RPTOR,TCF3,TNF
NF-κB Activation by Viruses	1.28E+01	-2.309	CCR5,EIF2AK2,ITGA2,LCK,NFKB1,NFKBIA,NRAS,PIK3CD,PIK3CG,PIK3R1,PRKCA,TBP,TNFRSF14

MSP-RON Signaling in Macrophages Pathway	1.27E+01	0.775	HLA-DMA,HLA-DMB,HLA-DOB,HLA-DQA1,HLA-DQB1,HLA-DRB5,IFNGR2,NFKB1,NRAS,PIK3CD,PIK3CG,PIK3R1,SBNO2,TLR4,TNF
Interleukin-10 signaling	1.27E+01	-2.111	ATP,CCL20,CCR2,CCR5,CD80,CD86,CSF1,IL18,IL1RN,JAK1,TNF
Role of Osteoblasts, Osteoclasts and Chondrocytes in Rheumatoid Arthritis	1.27E+01	NaN	AXIN1,BCL2,CASP9,CSF1,CTNNB1,IL18,IL1RN,MAP3K5,MAPK10,NFKB1,NFKBIA,PIK3CD,PIK3CG,PIK3R1,TCF3,TGFB1,TNF,WNT10A,WNT2B
Glioblastoma Multiforme Signaling	1.26E+01	-1.155	AXIN1,CCND2,CCND3,CTNNB1,E2F3,MDM2,MYC,NF1,NRAS,PIK3CD,PIK3CG,PIK3R1,RPS6KB1,TCF3,TP53,WNT10A,WNT2B
Transcriptional regulation by RUNX3	1.26E+01	-2.496	ATP,CTNNB1,HES1,ITGA4,ITGAL,JAG1,KAT2B,MDM2,MYC,PSMB10,PSMB8,PSMC4,TGFB1,TP53
IL-33 Signaling Pathway	1.22E+01	-2.183	CASP1,CASP8,CASP9,CCL1,ICAM2,IL18,IL1RN,MAP3K8,MAPK10,MYD88,NFKB1,NFKBIA,PIK3CD,PIK3CG,PIK3R1,RPS6KB1,TNF
Necroptosis Signaling Pathway	1.21E+01	-1.807	ATP,CASP1,CASP8,CYBB,EIF2AK2,FASLG,GLUD1,IRF9,JAK1,MDM2,RBL2,RIPK3,TICAM1,TLR4,TNF,TP53
IL-17 Signaling	1.21E+01	-2.183	CCL11,CCL20,CD70,FASLG,IL15,IL18,JAK1,LTB,MAPK10,NFKB1,NRAS,PIK3CD,PIK3CG,PIK3R1,TGFB1,TNF,VEGFB
HMGB1 Signaling	1.19E+01	-2.111	CD70,FASLG,IFNGR2,IL15,IL18,KAT2B,LTB,MAPK10,NFKB1,NRAS,PIK3CD,PIK3CG,PIK3R1,TGFB1,TLR4,TNF
Interferon alpha/beta signaling	1.18E+01	-2.309	ATP,EGR1,HLA-E,HLA-G,IRF2,IRF4,IRF5,IRF8,IRF9,JAK1,OAS2,PSMB8
Myelination Signaling Pathway	1.17E+01	-2.4	ATP,AXIN1,BLK,CTNNB1,EIF4EBP1,FYN,HDAC3,ITGA2,LCK,NFKB1,NRAS,PIK3CD,PIK3CG,PIK3R1,RPS6KB1,RPTOR,SLC2A1,SREBF1,WNT10A,WNT2B,ZEB2
Neutrophil Extracellular Trap Signaling Pathway	1.17E+01	-2.294	BLK,CASP1,CASP8,CASP9,CCR5,CYBB,FYN,ITGA2,ITGAL,LCK,MAPK10,NFKB1,PIK3CD,PIK3CG,PIK3R1,PRKCA,RIPK3,SLC2A1,TLR2,TLR4,TLR7,TLR8,TNF
Prostate Cancer Signaling	1.17E+01	NaN	BCL2,CASP9,CTNNB1,E2F3,HDAC3,MDM2,NFKB1,NFKBIA,NRAS,PIK3CD,PIK3CG,PIK3R1,RBL2,TP53
Hepatic Cholestasis	1.15E+01	-1.698	ATP,CD70,ESR1,FASLG,IL15,IL18,IL1RN,IL6R,LTB,LY96,MAPK10,MYD88,NFKB1,NFKBIA,PRKCA,TGFB1,TLR4,TNF
S100 Family Signaling Pathway	1.12E+01	-2.556	ADORA2A,BCL2,CCL20,CCR2,CCR5,CCR9,CD36,CTNNB1,CYBB,ESR1,FCGR2B,IL18,JAK1,MAP3K8,MAPK10,MYD88,NFKB1,PIK3CD,PIK3CG,PIK3R1,PRKCA,TCF3,TGFB1,TLR4,TNF,TP53,Tpm1,VEGFB,WNT10A,WNT2B
Induction of Apoptosis by HIV1	1.11E+01	-2.111	BCL2,CASP8,CASP9,FASLG,MAP3K5,MAPK10,NFKB1,NFKBIA,TNF,TP53,TRAF1
Cell surface interactions at the vascular wall	1.10E+01	-3.153	ATP,CD2,CD47,CD48,CD74,CD84,FYN,ITGA2,ITGA4,ITGAL,LCK,NRAS,PECAM1,PIK3R1,SELL,SIRPA,TGFB1
Wound Healing Signaling Pathway	1.10E+01	-1.414	CD70,FASLG,IFNGR2,IL15,IL18,IL1RN,JAK1,LTB,MAPK10,NFKB1,NFKBIA,NRAS,PRKCA,TGFB1,TGFBR1,TGFBR2,TNF,VEGFB
Transcriptional Regulatory Network in Embryonic Stem Cells	1.09E+01	-0.258	CTNNB1,JAK1,JAK3,MYC,NRAS,PIK3CD,PIK3CG,PIK3R1,TCF3,TGFB1,TGFBR1,TGFBR2,TP53,WNT10A,WNT2B
HER-2 Signaling in Breast Cancer	1.08E+01	-1.698	BLK,CASP9,FYN,LCK,MDM2,MYC,NFKB1,NFKBIA,NRAS,PIK3CD,PIK3CG,PIK3R1,PRKCA,RBL2,RPS6KB1,RPTOR,TP53
Systemic Lupus Erythematosus in T Cell Signaling Pathway	1.08E+01	-3.138	B2M,CASP1,CASP8,CASP9,CD3G,CD70,CD80,CD86,ESR1,FASLG,HLA-DMA,HLA-DMB,HLA-DOB,HLA-DQA1,HLA-DQB1,HLA-DRB5,HLA-E,HLA-G,ICOS,IRF4,ITGAL,NRAS,PIK3CD,PIK3CG,PIK3R1,RPS6KB1,RPTOR
Senescence Pathway	1.07E+01	-1.213	CCND2,CCND3,E2F3,EIF4EBP1,KAT2B,MDM2,MRE11,NF1,NFKB1,NRAS,PIK3CD,PIK3CG,PIK3R1,RBL2,TGFB1,TGFBR1,TGFBR2,TLR2,TP53
Class I MHC mediated antigen processing and presentation	1.06E+01	-4.583	ATP,B2M,CD36,CTSS,CYBB,DTX3L,HERC6,HLA-E,HLA-G,LY96,MRC1,MYD88,PSMB10,PSMB8,PSMC4,TAP2,TAPBP,TLR1,TLR2,TLR4,UBA7
Germ Cell-Sertoli Cell Junction Signaling	1.05E+01	NaN	AXIN1,CTNNB1,ITGA2,MAP3K12,MAP3K5,MAP3K8,MAPK10,NRAS,PIK3CD,PIK3CG,PIK3R1,TGFB1,TGFBR1,TGFBR2,TNF
Human Embryonic Stem Cell Pluripotency	1.05E+01	-0.5	AXIN1,CTNNB1,JAK1,JAK3,MYC,NRAS,PIK3CD,PIK3CG,PIK3R1,PRKCA,TCF3,TGFB1,TGFBR1,TGFBR2,WNT10A,WNT2B
Sertoli Cell-Germ Cell Junction Signaling Pathway (Enhanced)	1.03E+01	-2.5	ATP,AXIN1,CTNNB1,ITGA2,MAP3K12,MAP3K5,MAP3K8,MAPK10,NFKB1,NRAS,PIK3CD,PIK3CG,PIK3R1,TGFB1,TGFBR1,TGFBR2,TNF
IL-8 Signaling	1.02E+01	-1.941	BCL2,CCND2,CCND3,CYBB,EIF4EBP1,GNG4,MAPK10,NFKB1,NFKBIA,NRAS,PIK3CD,PIK3CG,PIK3R1,PRKCA,RPS6KB1,VEGFB
Non-Small Cell Lung Cancer Signaling	1.02E+01	-0.378	CASP9,CCND2,CCND3,E2F3,HDAC3,NRAS,PIK3CD,PIK3CG,PIK3R1,PRKCA,RBL2,TP53
PTEN Signaling	1.02E+01	1.732	BCL2,CASP9,FASLG,ITGA2,ITGA4,ITGAL,NFKB1,NRAS,PIK3CD,PIK3CG,PIK3R1,RPS6KB1,TGFBR1,TGFBR2

IL-7 Signaling Pathway	1.00E+01	-0.905	BCL2,CCND3,FYN,IL2RG,JAK1,JAK3,MYC,PIK3CD,PIK3CG,PIK3R1,SLC2A1
GADD45 Signaling	1.00E+01	-0.632	CCND2,CCND3,MAPK10,MYC,NFKB1,TGFB1,TGFBR1,TGFBR2,TNF,TP53
Type I Diabetes Mellitus Signaling	9.94E+00	-1.387	BCL2,CASP8,CASP9,CD3G,CD80,CD86,FASLG,HLA-DMA,HLA-DMB,HLA-DOB,HLA-DQA1,HLA-DQB1,HLA-DRB5,HLA-E,HLA-G,IFNGR2,JAK1,MAP3K5,MAPK10,MYD88,NFKB1,NFKBIA,TNF
Endometrial Cancer Signaling	9.88E+00	-0.707	AXIN1,CASP9,CTNNB1,MLH1,MYC,NRAS,PIK3CD,PIK3CG,PIK3R1,TP53
CTLA4 Signaling in Cytotoxic T Lymphocytes	9.85E+00	3.4	B2M,BLK,CD3G,CD80,CD86,CTLA4,FYN,HLA-DMA,HLA-DMB,HLA-DOB,HLA-DQA1,HLA-DQB1,HLA-DRB5,HLA-E,HLA-G,ITGA2,ITGAL,LCK,MAPK10,NFKB1,NRAS,PIK3CD,PIK3CG,PIK3R1,TGFB1
TCR signaling	9.76E+00	-3.051	ATP,CD3G,HLA-DQA1,HLA-DQB1,HLA-DRB5,LCK,NFKB1,NFKBIA,PIK3R1,PSMB10,PSMB8,PSMC4,PTPRC
Toll-like Receptor Cascades	9.76E+00	-2.828	CD36,LY96,TLR1,TLR2,TLR4,TLR7,TLR8,TLR9
HOTAIR Regulatory Pathway	9.73E+00	-1.604	CTNNB1,ESR1,MDM2,MYC,NFKB1,NFKBIA,PIK3CD,PIK3CG,PIK3R1,TCF3,TGFB1,TLR4,WNT10A,WNT2B
Interleukin-2 family signaling	9.56E+00	-2.333	ATP,CSF2RB,IL21R,IL2RG,JAK1,JAK3,LCK,PIK3CD,PIK3R1
IL-13 Signaling Pathway	9.42E+00	-0.577	BLK,CCL11,CD36,CTNNB1,FYN,JAK1,LCK,PIK3CD,PIK3CG,PIK3R1,TGFB1,TP53
Role of Chondrocytes in Rheumatoid Arthritis Signaling Pathway	9.41E+00	-1.941	CASP1,CASP8,CASP9,EGR1,IL18,IL1RN,IL6R,NFKB1,RIPK3,RPTOR,TLR2,TNF,VEGFB
Hereditary Breast Cancer Signaling	9.41E+00	NaN	ARID1A,CCND2,CCND3,HDAC3,MLH1,MRE11,NRAS,PIK3CD,PIK3CG,PIK3R1,PMS2,POLR2A,TP53
IL-17A Signaling in Airway Cells	9.40E+00	-2.333	CCL11,CCL20,JAK1,JAK3,MAPK10,NFKB1,NFKBIA,PIK3CD,PIK3CG,PIK3R1
Inflammasome pathway	9.31E+00	-2.646	ATP,CASP1,CASP8,IL18,MYD88,NFKB1,TLR4
MYC Mediated Apoptosis Signaling	9.31E+00	-0.333	BCL2,CASP8,CASP9,FASLG,MDM2,MYC,NFKB1,TNF,TP53
IL-9 Signaling	9.29E+00	-1.414	IL2RG,JAK1,JAK3,NFKB1,PIK3CD,PIK3CG,PIK3R1,TNF
PIP3 activates AKT signaling	9.23E+00	-3.051	ATP,CASP9,CD19,CD80,CD86,ESR1,FYN,ICOS,LCK,MDM2,MYD88,PIK3CD,PIK3R1
HIF1α Signaling	9.19E+00	-0.775	CYBB,EIF4EBP1,IL6R,LDHB,MDM2,NRAS,PIK3CD,PIK3CG,PIK3R1,PRKCA,RPS6KB1,SLC2A1,TGFB1,TP53,VEGFB
Signaling by ALK	9.19E+00	-2.121	ATP,CD274,HDAC3,IL2RG,JAK3,MYC,PIK3R1,PRDM1
Endocannabinoid Cancer Inhibition Pathway	9.05E+00	0.277	CASP1,CASP8,CASP9,CCND2,CCND3,CTNNB1,MYC,PIK3CD,PIK3CG,PIK3R1,RPTOR,TCF3,VEGFB
UVA-Induced MAPK Signaling	9.01E+00	-1.342	CASP9,MAPK10,NRAS,PARP4,PARP9,PIK3CD,PIK3CG,PIK3R1,PRKCA,RPS6KB1,TP53
Cytosolic sensors of pathogen-associated DNA	9.00E+00	-1.667	ATP,CTNNB1,IFI16,MRE11,MYD88,NFKB1,NFKBIA,RIPK3,TICAM1
NF-κB Signaling	8.99E+00	-3.838	CASP8,CD3G,EIF2AK2,IL18,IL1RN,LCK,MAP3K8,MYD88,NFKB1,NFKBIA,NRAS,PIK3CD,PIK3CG,PIK3R1,TGFBR1,TGFBR2,TLR1,TLR2,TLR4,TLR7,TLR8,TLR9,TNF
Caveolar-mediated Endocytosis Signaling	8.92E+00	NaN	B2M,CD48,FLNB,FYN,HLA-E,HLA-G,ITGA2,ITGA4,ITGAL,PRKCA
Granulocyte Adhesion and Diapedesis	8.89E+00	NaN	CCL1,CCL11,CCL20,CCR2,CCR5,CCR9,Cxcl3,ICAM2,IL18,IL1RN,ITGA4,PECAM1,SELL,TNF
Inhibition of Angiogenesis by TSP1	8.89E+00	-0.707	CD36,CD47,FYN,MAPK10,TGFB1,TGFBR1,TGFBR2,TP53
IL-6 Signaling	8.88E+00	-1.732	IL18,IL1RN,IL6R,MAPK10,NFKB1,NFKBIA,NRAS,PIK3CD,PIK3CG,PIK3R1,TNF,TNFAIP6
CD27 Signaling in Lymphocytes	8.86E+00	-0.707	CASP8,CASP9,CD70,MAP3K12,MAP3K5,MAP3K8,MAPK10,NFKB1,NFKBIA
Autophagy	8.84E+00	-0.258	BCL2,MAPK10,MYC,MYD88,NFKB1,PIK3CD,PIK3CG,PIK3R1,RPTOR,SLC1A5,TGFB1,TICAM1,TLR4,TNF,TP53
Mouse Embryonic Stem Cell Pluripotency	8.78E+00	-0.905	AXIN1,CTNNB1,JAK1,JAK3,MYC,NRAS,PIK3CD,PIK3CG,PIK3R1,TCF3,TP53
Apoptosis Signaling	8.78E+00	-0.302	BCL2,CASP8,CASP9,FASLG,MAP3K5,NFKB1,NFKBIA,NRAS,PRKCA,TNF,TP53
Cardiac Hypertrophy Signaling	8.73E+00	-2.324	ATP,EIF2B4,GNG4,IL6R,MAP3K12,MAP3K5,MAP3K8,MAPK10,NRAS,PIK3CD,PIK3CG,PIK3R1,RPS6KB1,TGFB1,TGFBR1,TGFBR2
Glioma Signaling	8.73E+00	-1.342	CCND2,CCND3,E2F3,HDAC3,MDM2,NRAS,PIK3CD,PIK3CG,PIK3R1,PRKCA,RBL2,TP53
MSP-RON Signaling Pathway	8.72E+00	NaN	CCR2,CSF1,CSF2RB,PIK3CD,PIK3CG,PIK3R1,TLR2,TLR4,TNF
Hepatic Fibrosis / Hepatic Stellate Cell Activation	8.69E+00	NaN	BCL2,CCR5,CSF1,FASLG,IFNGR2,IL6R,LY96,NFKB1,TGFB1,TGFBR1,TGFBR2,TLR4,TNF,VEGFB

HGF Signaling	8.65E+00	-2.714	ITGA2,ITGA4,ITGAL,MAP3K12,MAP3K5,MAP3K8,MAPK10,NRAS,PIK3CD,PIK3CG,PIK3R1,PRKCA
Folate Signaling Pathway	8.65E+00	-1.897	ATP,BCL2,CD274,JAK1,JAK3,MYC,NFKB1,OAS2,TP53,TYMS
STAT3 Pathway	8.62E+00	-1.897	BCL2,CSF2RB,IL21R,IL2RG,IL6R,MAP3K12,MAPK10,MYC,NRAS,TGFB1,TGFBR1,TGFBR2
Phagosome Formation	8.58E+00	-4.2	ADORA2A,CCR2,CCR5,CCR9,CD36,FCGR2B,FYN,ITGA2,ITGA4,ITGAL,LCK,MRC1,MYD88,NRAS,PIK3CD,PIK3CG,PIK3R1,PRKCA,RPS6KB1,TLR1,TLR2,TLR4,TLR7,TLR8,TLR9
PEDF Signaling	8.54E+00	-2.333	BCL2,CASP8,FASLG,NFKB1,NFKBIA,NRAS,PIK3CD,PIK3CG,PIK3R1,TP53
LPS-stimulated MAPK Signaling	8.49E+00	-1	MAP3K5,MAPK10,NFKB1,NFKBIA,NRAS,PIK3CD,PIK3CG,PIK3R1,PRKCA,TLR4
Estrogen Receptor Signaling	8.42E+00	-2.357	BCL2,EIF2B4,EIF4EBP1,ESR1,GNG4,HDAC3,HES1,JAK1,JAK3,MYC,NFKB1,NRAS,PIK3CD,PIK3CG,PIK3R1,PRKCA,RPS6KB1,TP53,VEGFB
Sertoli Cell-Sertoli Cell Junction Signaling	8.39E+00	-1.807	AXIN1,CTNNB1,ITGA2,MAP3K12,MAP3K5,MAP3K8,MAPK10,NFKB1,NRAS,PIK3CD,PIK3CG,PIK3R1,TGFBR1,TGFBR2,TNF
Agranulocyte Adhesion and Diapedesis	8.33E+00	NaN	CCL1,CCL11,CCL20,CCR2,CCR5,CCR9,Cxcl3,ICAM2,IL18,IL1RN,ITGA4,PECAM1,SELL,TNF
CGAS-STING Signaling Pathway	8.30E+00	-1.732	CASP1,CCL20,CD274,CD70,FASLG,IL15,IL18,LTB,NFKB1,NFKBIA,TGFB1,TNF
Huntington's Disease Signaling	8.29E+00	0.707	CASP1,CASP8,CASP9,GLS,GNG4,HDAC3,PIK3CD,PIK3CG,PIK3R1,POLR2A,PRKCA,PSMB10,PSMB8,PSMC4,TBP,TP53
T Helper Cell Differentiation	8.26E+00	NaN	CD3G,CD80,CD86,HLA-DMA,HLA-DMB,HLA-DOB,HLA-DQA1,HLA-DQB1,HLA-DRB5,ICOS,IFNGR2,IL18,IL21R,IL2RG,IL6R,TBX21,TGFB1,TGFBR1,TGFBR2,TNF
Neutrophil degranulation	8.18E+00	-3.13	ALDOA,B2M,CD36,CD47,CLEC5A,CMTM6,CTSS,CYBB,GUSB,ITGAL,LILRB3,NFKB1,NRAS,PECAM1,PTPRC,SELL,SIRPA,STK11IP,TLR2,TNFAIP6
UFMylation Signaling Pathway	8.16E+00	-0.707	ATP,ESR1,MDM2,MRE11,MYC,NFKB1,TLR4,TNF,TP53
IL-4 Signaling	8.14E+00	-2.985	CCL11,CD3G,EIF4EBP1,HLA-DMA,HLA-DMB,HLA-DOB,HLA-DQA1,HLA-DQB1,HLA-DRB5,IL2RG,IRF4,JAK1,JAK3,NFIL3,NFKB1,NRAS,PIK3CD,PIK3CG,PIK3R1,RPS6KB1,RPTOR,TGFB1
Signaling by the B Cell Receptor (BCR)	8.13E+00	-2.496	ATP,BLK,CD19,CD79B,FYN,NFKB1,NFKBIA,NRAS,PIK3CD,PIK3R1,PSMB10,PSMB8,PSMC4
DAP12 interactions	8.13E+00	-2.121	ATP,B2M,CLEC5A,FYN,HLA-E,LCK,NRAS,PIK3R1
p38 MAPK Signaling	8.12E+00	-1	FASLG,IL18,IL1RN,MAP3K5,MYC,RPS6KB1,TGFB1,TGFBR1,TGFBR2,TNF,TP53
Role of JAK1 and JAK3 in γc Cytokine Signaling	8.10E+00	NaN	IL15,IL21R,IL2RG,JAK1,JAK3,NRAS,PIK3CD,PIK3CG,PIK3R1
Cell Cycle: G1/S Checkpoint Regulation	8.10E+00	-0.707	CCND2,CCND3,E2F3,HDAC3,MDM2,MYC,RBL2,TGFB1,TP53
Immunogenic Cell Death Signaling Pathway	8.06E+00	-1.897	ATP,BCL2,CASP1,CASP8,CASP9,IFNGR2,NFKB1,RIPK3,TLR4,TNF
Acute Myeloid Leukemia Signaling	8.06E+00	-2.333	CSF2RB,EIF4EBP1,MYC,NFKB1,NRAS,PIK3CD,PIK3CG,PIK3R1,RPS6KB1,TCF3
Acute Phase Response Signaling	8.02E+00	-1.387	IL18,IL1RN,IL6R,MAP3K5,MYD88,NFKB1,NFKBIA,NRAS,PIK3CD,PIK3CG,PIK3R1,TCF3,TNF
iNOS Signaling	7.98E+00	-1.414	IFNGR2,JAK1,JAK3,LY96,MYD88,NFKB1,NFKBIA,TLR4
FAT10 Cancer Signaling Pathway	7.98E+00	1.414	CTNNB1,NFKB1,NFKBIA,TGFB1,TGFBR1,TGFBR2,TNF,TP53
Death Receptor Signaling	7.97E+00	-1.897	BCL2,CASP8,CASP9,FASLG,MAP3K5,NFKB1,NFKBIA,PARP4,PARP9,TNF
Virus Entry via Endocytic Pathways	7.97E+00	NaN	B2M,FLNB,FYN,HLA-E,HLA-G,ITGA2,NRAS,PIK3CD,PIK3CG,PIK3R1,PRKCA
Role of NANOG in Mammalian Embryonic Stem Cell Pluripotency	7.97E+00	-0.707	AXIN1,CTNNB1,JAK1,JAK3,NRAS,PIK3CD,PIK3CG,PIK3R1,TP53,WNT10A,WNT2B
MyD88 dependent cascade initiated on endosome	7.95E+00	-2.646	ATP,LY96,MYD88,TICAM1,TLR4,TLR7,TLR9
Deubiquitination	7.93E+00	-1.807	ATP,AXIN1,ESR1,IFIH1,KAT2B,MDM2,MYC,NFKBIA,PSMB10,PSMB8,PSMC4,TGFB1,TGFBR1,TGFBR2,TP53
NGF Signaling	7.89E+00	-3.317	MAP3K12,MAP3K5,MAP3K8,MAPK10,NFKB1,NRAS,PIK3CD,PIK3CG,PIK3R1,RPS6KB1,TP53
Serotonin Receptor Signaling	7.87E+00	-2.683	BCL2,BLK,CD38,EIF4EBP1,FYN,GNG4,LCK,MAP3K5,MYC,NFKB1,NFKBIA,NRAS,PIK3CD,PIK3CG,PIK3R1,PRKCA,RBL2,RPS6KB1,TGFB1,TNF
Role of Osteoclasts in Rheumatoid Arthritis Signaling Pathway	7.86E+00	-1.5	BCL2,CASP9,CSF1,CTNNB1,FCGR2B,IL18,MAP3K5,MAPK10,NFKB1,NFKBIA,NRAS,PIK3CD,PIK3CG,PIK3R1,TLR4,TNF

UVB-Induced MAPK Signaling	7.84E+00	-2.121	EIF4EBP1,MAPK10,PIK3CD,PIK3CG,PIK3R1,PRKCA,RPS6KB1,TP53
FXR/RXR Activation	7.75E+00	1.387	CASP8,CD70,FASLG,IL15,IL18,IL1RN,LTB,MAPK10,NFKB1,PCK2,SREBF1,TGFB1,TNF
Interleukin-1 family signaling	7.72E+00	-2.53	ATP,CASP1,IL18,IL1RN,MAP3K8,MYD88,NFKB1,NFKBIA,PSMB10,PSMB8,PSMC4
Atherosclerosis Signaling	7.61E+00	NaN	CCL11,CCR2,CD36,CSF1,IL18,IL1RN,ITGA4,NFKB1,TGFB1,TNF,TNFRSF14
NAD Signaling Pathway	7.52E+00	-1.508	CD38,LDHB,NFKB1,PARP4,PARP9,PIK3CD,PIK3CG,PIK3R1,POLR2A,SREBF1,TGFB1,TP53
Interleukin-3, Interleukin-5 and GM-CSF signaling	7.52E+00	-1.414	ATP,CSF2RB,FYN,IL2RG,JAK1,JAK3,PIK3CD,PIK3R1
Lymphotoxin β Receptor Signaling	7.52E+00	-2.828	CASP9,LTB,NFKB1,NFKBIA,PIK3CD,PIK3CG,PIK3R1,TRAF1
CLEAR Signaling Pathway	7.43E+00	1.291	GUSB,NRAS,PRKCA,RPTOR,TGFB1,TGFBR1,TGFBR2,TLR1,TLR2,TLR4,TLR7,TLR8,TLR9,TNF,TP53
MSP-RON Signaling in Cancer Cells Pathway	7.42E+00	-2.714	CSF2RB,CTNNB1,MYC,NFKB1,NFKBIA,NRAS,PIK3CD,PIK3CG,PIK3R1,TCF3,VEGFB
Gap Junction Signaling	7.32E+00	-0.5	ATP,CTNNB1,MAP3K5,MAPK10,MYC,NFKB1,NRAS,PIK3CD,PIK3CG,PIK3R1,PRKCA,TCF3,TGFB1,TGFBR1,TLR4,TNF
Insulin Secretion Signaling Pathway	7.28E+00	-0.775	ATP,EIF2B4,EIF4EBP1,FYN,JAK1,JAK3,LCK,PC,PIK3CD,PIK3CG,PIK3R1,PRKCA,RPS6KB1,SLC2A1,TCF3
Insulin Receptor Signaling	7.23E+00	-1.897	ATP,EIF2B4,EIF4EBP1,FYN,JAK1,NRAS,PIK3CD,PIK3CG,PIK3R1,RPS6KB1,RPTOR
ERK/MAPK Signaling	7.20E+00	-1.732	DUSP2,EIF4EBP1,ESR1,FYN,ITGA2,ITGA4,ITGAL,MYC,NRAS,PIK3CD,PIK3CG,PIK3R1,PRKCA
IL-2 Signaling	7.11E+00	-2.121	IL2RG,JAK1,JAK3,LCK,NRAS,PIK3CD,PIK3CG,PIK3R1
WNT/SHH Axonal Guidance Signaling Pathway	7.08E+00	-0.905	CTNNB1,FYN,LCK,MAPK10,PIK3CD,PIK3CG,PIK3R1,PRKCA,TCF3,WNT10A,WNT2B
Cancer Drug Resistance by Drug Efflux	7.06E+00	-1.89	ATP,MDM2,NFKB1,NRAS,PIK3CD,PIK3CG,PIK3R1,TP53
PKCθ Signaling in T Lymphocytes	7.00E+00	-3.578	CD3G,CD80,CD86,FYN,HLA-DMA,HLA-DMB,HLA-DOB,HLA-DQA1,HLA-DQB1,HLA-DRB5,LCK,MAP3K12,MAP3K5,MAP3K8,NFKB1,NFKBIA,NRAS,PIK3CD,PIK3CG,PIK3R1
Factors Promoting Cardiogenesis in Vertebrates	6.99E+00	-0.302	AXIN1,CTNNB1,MAPK10,MYC,PRKCA,TCF3,TGFB1,TGFBR1,TGFBR2,WNT10A,WNT2B
RANK Signaling in Osteoclasts	6.99E+00	-2.828	MAP3K12,MAP3K5,MAP3K8,MAPK10,NFKB1,NFKBIA,PIK3CD,PIK3CG,PIK3R1
FAK Signaling	6.98E+00	-3.402	ADORA2A,CCR2,CCR5,CCR9,CD3G,CSF2RB,CTNNB1,FYN,IL21R,IL2RG,IL6R,ITGA2,ITGA4,ITGAL,LCK,MAPK10,MDM2,MYC,NFKB1,NRAS,PIK3CD,PIK3CG,PIK3R1,TCF3,TGFB1,TGFBR1,TGFBR2,TP53
CD40 Signaling	6.95E+00	-2.828	JAK3,MAPK10,NFKB1,NFKBIA,PIK3CD,PIK3CG,PIK3R1,TRAF1
PDGF Signaling	6.95E+00	-1.667	EIF2AK2,JAK1,JAK3,MYC,NRAS,PIK3CD,PIK3CG,PIK3R1,PRKCA
Role of NFAT in Cardiac Hypertrophy	6.93E+00	-1.265	ATP,GNG4,HDAC3,IL6R,MAPK10,NRAS,PIK3CD,PIK3CG,PIK3R1,PRKCA,TGFB1,TGFBR1,TGFBR2
Role of PI3K/AKT Signaling in the Pathogenesis of Influenza	6.90E+00	-2.449	CASP9,CCR5,MLH1,NFKB1,NFKBIA,PIK3CD,PIK3CG,PIK3R1
Trafficking and processing of endosomal TLR	6.90E+00	-2.236	ATP,CTSS,TLR7,TLR8,TLR9
MyD88:MAL(TIRAP) cascade initiated on plasma membrane	6.89E+00	-2.646	ATP,CD36,LY96,MYD88,TLR1,TLR2,TLR4
MIF Regulation of Innate Immunity	6.83E+00	-1.134	CD74,LY96,MAPK10,NFKB1,NFKBIA,TLR4,TP53
CD28 Signaling in T Helper Cells	6.82E+00	-2.111	CD3G,CD80,CD86,CTLA4,FYN,HLA-DMA,HLA-DMB,HLA-DOB,HLA-DQA1,HLA-DQB1,HLA-DRB5,LCK,MAPK10,NFKB1,NFKBIA,PIK3CD,PIK3CG,PIK3R1,PTPRC
TNFs bind their physiological receptors	6.82E+00	-1.633	CD70,FASLG,TNFRSF14,TNFRSF8,TNFRSF9,TNFSF18
p53 Signaling	6.75E+00	0.707	BCL2,CCND2,CTNNB1,KAT2B,MDM2,PIK3CD,PIK3CG,PIK3R1,TP53
Caspase activation via Death Receptors in the presence of ligand	6.74E+00	-2.236	CASP8,FASLG,LY96,TICAM1,TLR4
Interleukin-15 signaling	6.74E+00	-1.342	ATP,IL15,IL2RG,JAK1,JAK3
Aryl Hydrocarbon Receptor Signaling	6.71E+00	0.302	CCND2,CCND3,ESR1,FASLG,MDM2,MYC,NFKB1,RBL2,TGFB1,TNF,TP53
HEY1 Signaling Pathway	6.69E+00	-0.905	E2F3,JAG1,MDM2,PIK3CD,PIK3CG,PIK3R1,TGFB1,TGFBR1,TGFBR2,TP53,VEGFB
Opioid Signaling Pathway	6.61E+00	-1.732	ATP,BLK,CTNNB1,FYN,GNG4,LCK,MYC,NFKB1,NFKBIA,NRAS,PIK3CG,PRKCA,RPS6KB1,TCF3

Mitotic G1 phase and G1/S transition	6.58E+00	-1.265	ATP,CCND2,CCND3,E2F3,MYC,PSMB10,PSMB8,PSMC4,RBL2,TYMS
RAR Activation	6.50E+00	-0.243	ARID1A,CD70,CSF2RB,FASLG,IL15,IL18,KAT2B,LTB,MAPK10,NFKB1,PIK3CD,PIK3CG,PIK3R1,TGFB1,TGFBR1,TGFBR2,TNF
Type II Diabetes Mellitus Signaling	6.45E+00	-1.265	ATP,CD36,MAP3K5,MAPK10,NFKB1,NFKBIA,PIK3CD,PIK3CG,PIK3R1,PRKCA,TNF
WNT/β-catenin Signaling	6.45E+00	-1.265	AXIN1,CTNNB1,MDM2,MYC,TCF3,TGFB1,TGFBR1,TGFBR2,TP53,WNT10A,WNT2B
RAC Signaling	6.41E+00	-3	CYBB,ITGA2,ITGA4,ITGAL,NFKB1,NRAS,PIK3CD,PIK3CG,PIK3R1,RPS6KB1
4-1BB Signaling in T Lymphocytes	6.38E+00	-2.236	MAP3K5,MAPK10,NFKB1,NFKBIA,TNFRSF9,TRAF1
Thyroid Cancer Signaling	6.31E+00	-2.121	CTNNB1,MYC,NRAS,PIK3CD,PIK3CG,PIK3R1,TCF3,TP53
ICOS-ICOSL Signaling in T Helper Cells	6.27E+00	-3.742	CD3G,CD80,CD86,HLA-DMA,HLA-DMB,HLA-DOB,HLA-DQA1,HLA-DQB1,HLA-DRB5,ICOS,IL2RG,LCK,NFKB1,NFKBIA,PIK3CD,PIK3CG,PIK3R1,PTPRC
Role of MAPK Signaling in Inhibiting the Pathogenesis of Influenza	6.26E+00	-2.121	EIF2AK2,MAP3K5,MAPK10,MYD88,NFKB1,NFKBIA,TLR4,TNF
Docosahexaenoic Acid (DHA) Signaling	6.23E+00	-0.277	BCL2,CASP8,CASP9,CCL20,FASLG,PIK3CD,PIK3CG,PIK3R1,PRKCA,RPS6KB1,RPTOR,TGFB1,TNF
Altered T Cell and B Cell Signaling in Rheumatoid Arthritis	6.22E+00	NaN	CD3G,CD79B,CD80,CD86,CSF1,FASLG,HLA-DMA,HLA-DMB,HLA-DOB,HLA-DQA1,HLA-DQB1,HLA-DRB5,IL15,IL18,IL1RN,LTB,NFKB1,TGFB1,TLR1,TLR2,TLR4,TLR7,TLR8,TLR9,TNF
Sleep REM Signaling Pathway	6.15E+00	0.333	CD70,EGR1,FASLG,IL15,IL18,LTB,RPS6KB1,TGFB1,TNF
Role of IL-17A in Arthritis	6.13E+00	NaN	CCL20,MAPK10,NFKB1,NFKBIA,PIK3CD,PIK3CG,PIK3R1
TEC Kinase Signaling	6.12E+00	-2.138	BLK,CD3G,FASLG,FYN,GNG4,ITGA2,ITGA4,ITGAL,JAK1,JAK3,LCK,MAPK10,NFKB1,PIK3CD,PIK3CG,PIK3R1,PRKCA,TLR4,TNF
IL-17A Signaling in Fibroblasts	6.06E+00	0.707	BCL2,MAPK10,NFKB1,NFKBIA,P4HA1,PRKCA,TGFB1,TNF
BAG2 Signaling Pathway	6.06E+00	1	ATP,MDM2,MYC,NFKB1,PSMB10,PSMB8,PSMC4,TP53
RAF/MAP kinase cascade	6.05E+00	-1.941	ATP,CSF2RB,DUSP2,FYN,IL2RG,JAK1,JAK3,NF1,NRAS,PIK3R1,PSMB10,PSMB8,PSMC4
Primary Immunodeficiency Signaling	6.03E+00	NaN	CD19,ICOS,IL2RG,JAK3,LCK,PTPRC,TAP2
PAK Signaling	6.03E+00	-2.121	ITGA2,ITGA4,ITGAL,MAPK10,NRAS,PIK3CD,PIK3CG,PIK3R1,TNF
MyD88-independent TLR4 cascade	6.01E+00	-2.449	ATP,CASP8,LY96,RIPK3,TICAM1,TLR4
MicroRNA Biogenesis Signaling Pathway	5.90E+00	-0.905	ATP,ESR1,MYC,NRAS,PIK3CD,PIK3CG,PIK3R1,POLR2A,RPS6KB1,TGFB1,TP53
Leukocyte Extravasation Signaling	5.88E+00	-1.897	CTNNB1,CYBB,ITGA2,ITGA4,ITGAL,MAPK10,PECAM1,PIK3CD,PIK3CG,PIK3R1,PRKCA
Interleukin-7 signaling	5.87E+00	-1	ATP,IL2RG,JAK1,JAK3,PIK3R1
Degradation of beta-catenin by the destruction complex	5.81E+00	0	ATP,AXIN1,CTNNB1,MYC,PSMB10,PSMB8,PSMC4,TCF3
Interleukin-9 signaling	5.78E+00	-1	ATP,IL2RG,JAK1,JAK3
DHCR24 Signaling Pathway	5.78E+00	-0.632	ESR1,MDM2,NFKB1,NRAS,PIK3CD,PIK3CG,PIK3R1,PRKCA,SREBF1,TP53
C-type lectin receptors (CLRs)	5.75E+00	-1.667	ATP,CASP8,FYN,ICAM2,NFKB1,NFKBIA,NRAS,PSMB10,PSMB8,PSMC4
Acetylcholine Receptor Signaling Pathway	5.73E+00	0.302	BCL2,CASP1,CASP8,CASP9,FYN,NFKB1,PIK3CD,PIK3CG,PIK3R1,PRKCA,TNF
Renin-Angiotensin Signaling	5.71E+00	-1.667	ATP,MAPK10,NFKB1,NRAS,PIK3CD,PIK3CG,PIK3R1,PRKCA,TNF
MHC class II antigen presentation	5.68E+00	-3	ATP,CD74,CTSS,HLA-DMA,HLA-DMB,HLA-DOB,HLA-DQA1,HLA-DQB1,HLA-DRB5
Endothelin-1 Signaling	5.67E+00	-2.714	ATP,CASP1,CASP8,CASP9,MAPK10,MYC,NRAS,PIK3CD,PIK3CG,PIK3R1,PRKCA
SPINK1 General Cancer Pathway	5.66E+00	-1.89	IL6R,JAK1,JAK3,NRAS,PIK3CD,PIK3CG,PIK3R1
Gα12/13 Signaling	5.60E+00	-2.121	CTNNB1,MAP3K5,MAPK10,NFKB1,NFKBIA,NRAS,PIK3CD,PIK3CG,PIK3R1
DNA damage-induced 14-3-3σ Signaling	5.58E+00	-0.816	BCL2,CASP1,CASP8,CASP9,MDM2,TP53
IL-23 Signaling Pathway	5.58E+00	-1.633	NFKB1,NFKBIA,PIK3CD,PIK3CG,PIK3R1,TNF
TR/RXR Activation	5.57E+00	-1.667	HDAC3,MDM2,PIK3CD,PIK3CG,PIK3R1,RPS6KB1,SLC2A1,SREBF1,VEGFB

fMLP Signaling in Neutrophils	5.49E+00	-2.121	CYBB,GNG4,NFKB1,NFKBIA,NRAS,PIK3CD,PIK3CG,PIK3R1,PRKCA
VEGF Signaling	5.44E+00	-2.646	BCL2,EIF2B4,NRAS,PIK3CD,PIK3CG,PIK3R1,PRKCA,VEGFB
Signaling by SCF-KIT	5.41E+00	-0.816	ATP,FYN,LCK,NRAS,PIK3R1,PRKCA
Paxillin Signaling	5.38E+00	-2.646	ITGA2,ITGA4,ITGAL,MAPK10,NRAS,PIK3CD,PIK3CG,PIK3R1
Macropinocytosis Signaling	5.38E+00	-1.633	CSF1,MRC1,NRAS,PIK3CD,PIK3CG,PIK3R1,PRKCA
P2Y Purigenic Receptor Signaling Pathway	5.37E+00	-1.89	ATP,GNG4,MYC,NFKB1,NRAS,PIK3CD,PIK3CG,PIK3R1,PRKCA
Protein Kinase A Signaling	5.36E+00	1	ATP,CTNNB1,DUSP2,FLNB,GNG4,IHH,NFKB1,NFKBIA,PRKCA,PTPRC,SIRPA,TCF3,TGFB1,TGFBR1,TGFBR2
Telomerase Signaling	5.35E+00	-1.89	HDAC3,IL2RG,MYC,NRAS,PIK3CD,PIK3CG,PIK3R1,TP53
Breast Cancer Regulation by Stathmin1	5.33E+00	-2.324	ADORA2A,CCND2,CCND3,CCR2,CCR5,CCR9,E2F3,GNG4,NFKB1,NRAS,PIK3CD,PIK3CG,PIK3R1,PRKCA,RPS6KB1,TGFB1,TP53,VEGFB
Sumoylation Pathway	5.32E+00	-1.134	ATP,FASLG,MAP3K5,MAPK10,MDM2,NFKB1,NFKBIA,TP53
Angiopoietin Signaling	5.30E+00	NaN	CASP9,NFKB1,NFKBIA,NRAS,PIK3CD,PIK3CG,PIK3R1
Sirtuin Signaling Pathway	5.29E+00	0	ATP,GLS,GLUD1,GOT2,LDHB,MYC,NFKB1,PCK2,RPTOR,SLC2A1,SREBF1,TNF,TP53
Role of p14/p19ARF in Tumor Suppression	5.27E+00	1.342	MDM2,PIK3CD,PIK3CG,PIK3R1,TP53
IL-3 Signaling	5.27E+00	-1.134	CSF2RB,JAK1,NRAS,PIK3CD,PIK3CG,PIK3R1,PRKCA
Melanoma Signaling	5.26E+00	-1.633	MDM2,NRAS,PIK3CD,PIK3CG,PIK3R1,TP53
Regulation of eIF4 and p70S6K Signaling	5.24E+00	-2.646	EIF2B4,EIF4EBP1,ITGA2,ITGA4,ITGAL,NRAS,PIK3CD,PIK3CG,PIK3R1,RPS6KB1
Apelin Endothelial Signaling Pathway	5.22E+00	-2.121	GNG4,MAPK10,NFKB1,NRAS,PIK3CD,PIK3CG,PIK3R1,PRKCA,RPS6KB1
Regulation of TLR by endogenous ligand	5.20E+00	-2.236	CD36,LY96,TLR1,TLR2,TLR4
JAK/STAT Signaling	5.16E+00	-1.89	JAK1,JAK3,NFKB1,NRAS,PIK3CD,PIK3CG,PIK3R1
Bladder Cancer Signaling	5.12E+00	NaN	E2F3,HDAC3,MDM2,MYC,NRAS,RBL2,TP53,VEGFB
BEX2 Signaling Pathway	5.09E+00	-1.89	BCL2,CTNNB1,MAPK10,NFKB1,NFKBIA,TCF3,VEGFB
Graft-versus-Host Disease Signaling	5.07E+00	NaN	CD3G,CD80,CD86,FASLG,HLA-DMA,HLA-DMB,HLA-DOB,HLA-DQA1,HLA-DQB1,HLA-DRB5,HLA-E,HLA-G,IL18,IL1RN,TNF
Airway Pathology in Chronic Obstructive Pulmonary Disease	5.07E+00	NaN	CCL20,CD70,FASLG,IL15,IL18,LTB,TGFB1,TNF
GNRH Signaling	5.07E+00	-0.707	ATP,EGR1,GNG4,MAP3K12,MAP3K5,MAP3K8,MAPK10,NFKB1,NRAS,PRKCA
Transcriptional regulation by RUNX1	5.06E+00	-2.121	ARID1A,ATP,AXIN1,BLK,ESR1,PSMB10,PSMB8,PSMC4,TCF3
Signaling by Erythropoietin	5.06E+00	-2.236	ATP,NRAS,PIK3CD,PIK3CG,PIK3R1
PPARα/RXRα Activation	5.03E+00	0.333	ATP,CD36,GOT2,NFKB1,NFKBIA,NRAS,PRKCA,TGFB1,TGFBR1,TGFBR2
CNTF Signaling	5.02E+00	-1.633	JAK1,NRAS,PIK3CD,PIK3CG,PIK3R1,RPS6KB1
Signaling by NOTCH4	5.02E+00	-1.89	ATP,HES1,JAG1,KAT2B,PSMB10,PSMB8,PSMC4
Cyclins and Cell Cycle Regulation	5.02E+00	0.447	CCND2,CCND3,E2F3,HDAC3,RBL2,TGFB1,TP53
Regulation of the Epithelial Mesenchymal Transition in Development Pathway	4.99E+00	0	AXIN1,CTNNB1,JAG1,NFKB1,TCF3,WNT10A,WNT2B
NUR77 Signaling in T Lymphocytes	4.97E+00	-1.134	B2M,BCL2,CASP9,CD3G,CD80,CD86,FASLG,HLA-DMA,HLA-DMB,HLA-DOB,HLA-DQA1,HLA-DQB1,HLA-DRB5,HLA-E,HLA-G,PRKCA
Neuregulin Signaling	4.96E+00	-1.342	ITGA2,ITGA4,ITGAL,MYC,NRAS,PIK3R1,PRKCA,RPS6KB1
ILK Signaling	4.95E+00	-1.897	CTNNB1,FLNB,MAPK10,MYC,NFKB1,PIK3CD,PIK3CG,PIK3R1,TNF,VEGFB
Xenobiotic Metabolism Signaling	4.95E+00	NaN	CES3,MAP3K12,MAP3K5,MAP3K8,MGMT,NFKB1,NRAS,PIK3CD,PIK3CG,PIK3R1,PRKCA,TNF
IL-15 Production	4.93E+00	-1.414	BLK,FYN,IL15,JAK1,JAK3,LCK,NFKB1,TWF1
Oxytocin in Brain Signaling Pathway	4.93E+00	-0.632	CASP1,GNG4,NFKB1,NRAS,PIK3CD,PIK3CG,PIK3R1,PRKCA,TLR4,TNF
Fc Epsilon RI Signaling	4.91E+00	-1.134	FYN,MAPK10,NRAS,PIK3CD,PIK3CG,PIK3R1,PRKCA,TNF

EGF Signaling	4.89E+00	-0.816	JAK1,PIK3CD,PIK3CG,PIK3R1,PRKCA,RPS6KB1
Amyotrophic Lateral Sclerosis Signaling	4.88E+00	-2.121	BCL2,CASP1,CASP9,PIK3CD,PIK3CG,PIK3R1,TP53,VEGF B
Adrenomedullin signaling pathway	4.88E+00	-1.897	BCL2,IL18,IL1RN,MAPK10,NFKB1,NRAS,PIK3CD,PIK3CG, PIK3R1,TNF
Interferon Signaling	4.87E+00	0	BCL2,IFNGR2,IRF9,JAK1,PSMB8
ESR-mediated signaling	4.86E+00	-1.134	ATP,AXIN1,BCL2,ESR1,KAT2B,MYC,POLR2A,TBP
NLR signaling pathways	4.85E+00	-2.449	ATP,BCL2,CASP1,CASP8,CASP9,NFKB1
G-Protein Coupled Receptor Signaling	4.83E+00	-2.357	ADORA2A,ATP,CCR2,CCR5,CCR9,CTNNB1,FYN,GNG4,M AP3K12,MAP3K5,MAP3K8,MAPK10,NFKB1,NFKBIA,NRAS, PIK3CD,PIK3CG,PIK3R1,PRKCA
Transcriptional regulation of white adipocyte differentiation	4.83E+00	-0.378	CCND3,CD36,HDAC3,NFKB1,SREBF1,TGFB1,TNF
Role of JAK2 in Hormone-like Cytokine Signaling	4.81E+00	0	BCL2,CCND2,CCND3,JAK1,SIRPA,VEGFB
ROBO SLIT Signaling Pathway	4.81E+00	0	BLK,CTNNB1,FYN,HES1,LCK,TGFB1,TGFBR2,VEGFB
Endocannabinoid Developing Neuron Pathway	4.81E+00	-1.89	CTNNB1,GNG4,MAPK10,NRAS,PIK3CD,PIK3CG,PIK3R1,R PTOR
TWEAK Signaling	4.81E+00	-1.342	CASP8,CASP9,NFKB1,NFKBIA,TRAF1
AMPK Signaling	4.80E+00	-1.414	ARID1A,EIF4EBP1,GNG4,KAT2B,PCK2,PIK3CD,PIK3CG,PI K3R1,RPS6KB1,RPTOR,SLC2A1
ERBB Signaling	4.80E+00	-1.89	MAPK10,NRAS,PIK3CD,PIK3CG,PIK3R1,PRKCA,RPS6KB1
14-3-3-mediated Signaling	4.78E+00	-1.414	MAP3K5,MAPK10,NRAS,PIK3CD,PIK3CG,PIK3R1,PRKCA, TNF
eNOS Signaling	4.78E+00	-2.333	ATP,CASP8,CASP9,ESR1,PIK3CD,PIK3CG,PIK3R1,PRKCA ,VEGFB
Eicosanoid Signaling	4.77E+00	-1.732	CTNNB1,EGR1,GNG4,MYC,NFKB1,NRAS,PIK3CD,PIK3CG, PIK3R1,PRKCA,TCF3,VEGFB
Signaling by VEGF	4.76E+00	-0.707	ATP,CTNNB1,CYBB,FYN,NRAS,PIK3R1,PRKCA,VEGFB
LXR/RXR Activation	4.76E+00	1.134	CD36,IL18,IL1RN,LY96,NFKB1,SREBF1,TLR4,TNF
MIF-mediated Glucocorticoid Regulation	4.75E+00	-1.342	CD74,LY96,NFKB1,NFKBIA,TLR4
Pancreatic Secretion Signaling Pathway	4.74E+00	-2.111	ADORA2A,ATP,CD38,EIF4EBP1,IRF2,PIK3CD,PIK3CG,PIK 3R1,PRKCA,RPS6KB1,RPTOR
Ceramide Signaling	4.73E+00	1.134	BCL2,NFKB1,NRAS,PIK3CD,PIK3CG,PIK3R1,TNF
Activation of IRF by Cytosolic Pattern Recognition Receptors	4.69E+00	-0.816	IFIH1,IRF9,MAPK10,NFKB1,NFKBIA,TNF
ERB2-ERBB3 Signaling	4.69E+00	-2.449	JAK3,MYC,NRAS,PIK3CD,PIK3CG,PIK3R1
Prolactin Signaling	4.65E+00	-1.134	FYN,MYC,NRAS,PIK3CD,PIK3CG,PIK3R1,PRKCA
CXCR4 Signaling	4.64E+00	-1.414	ATP,EGR1,GNG4,MAPK10,NRAS,PIK3CD,PIK3CG,PIK3R1, PRKCA
Mismatch Repair in Eukaryotes	4.64E+00	-1	ATP,EXO1,MLH1,PMS2
Signaling by CSF1 (M-CSF) in myeloid cells	4.64E+00	-1.342	ATP,CSF1,FYN,PIK3CG,PIK3R1
Signaling by Rho Family GTPases	4.63E+00	-3	CYBB,GNG4,ITGA2,ITGA4,ITGAL,MAP3K12,MAPK10,NFKB 1,PIK3CD,PIK3CG,PIK3R1
Thrombopoietin Signaling	4.61E+00	-1.633	MYC,NRAS,PIK3CD,PIK3CG,PIK3R1,PRKCA
Fc epsilon receptor (FCERI) signaling	4.60E+00	-1.667	ATP,FYN,MAPK10,NFKB1,NFKBIA,NRAS,PIK3R1,PSMB10, PSMB8,PSMC4
Melanocyte Development and Pigmentation Signaling	4.59E+00	-2.449	ATP,BCL2,NRAS,PIK3CD,PIK3CG,PIK3R1,RPS6KB1
FLT3 Signaling	4.53E+00	-1.342	ATP,FYN,LCK,NRAS,PIK3R1
Adipogenesis pathway	4.53E+00	-2.121	CTNNB1,HDAC3,KAT2B,RPS6KB1,SREBF1,TGFB1,TNF,TP 53
GPVI-mediated activation cascade	4.48E+00	-1.342	ATP,FYN,LCK,PIK3CG,PIK3R1
Circadian Rhythm Signaling	4.46E+00	NaN	BLK,EIF4EBP1,FYN,GNG4,LCK,MDM2,MYC,NFIL3,NRAS,P RKCA,RPTOR
EIF2 Signaling	4.44E+00	-1.897	BCL2,EIF2AK2,EIF2B4,MYC,NRAS,PIK3CD,PIK3CG,PIK3R 1,RPL23,SREBF1
Basal Cell Carcinoma Signaling	4.40E+00	0.447	AXIN1,CTNNB1,TCF3,TP53,WNT10A,WNT2B
IGF-1 Signaling	4.40E+00	-1.134	CASP9,JAK1,NRAS,PIK3CD,PIK3CG,PIK3R1,RPS6KB1

Xenobiotic Metabolism General Signaling Pathway	4.40E+00	-2.121	MAP3K12,MAP3K5,MAP3K8,NRAS,PIK3CD,PIK3CG,PIK3R1,PRKCA
Mismatch Repair	4.38E+00	-1	ATP,EXO1,MLH1,PMS2
Tumoricidal Function of Hepatic Natural Killer Cells	4.31E+00	NaN	CASP8,CASP9,FASLG,ITGAL
TAK1-dependent IKK and NF-kappa-B activation	4.29E+00	-1.342	ATP,CASP8,NFKB1,NFKBIA,TP53
Apelin Pancreas Signaling Pathway	4.29E+00	-0.447	MAPK10,NFKB1,PIK3CD,PIK3CG,PIK3R1
Semaphorin Neuronal Repulsive Signaling Pathway	4.27E+00	2.828	FYN,ITGA2,ITGA4,ITGAL,PIK3CD,PIK3CG,PIK3R1,TP53
WNK Renal Signaling Pathway	4.25E+00	-0.447	ATP,MDM2,PIK3CD,PIK3CG,PIK3R1,PRKCA,TNF
Role of JAK family kinases in IL-6-type Cytokine Signaling	4.21E+00	-0.816	IL6R,JAK1,MAPK10,MYC,TGFB1,VEGFB
Role of BRCA1 in DNA Damage Response	4.17E+00	0	ARID1A,E2F3,MLH1,MRE11,RBL2,TP53
RAF-independent MAPK1/3 activation	4.16E+00	NaN	ATP,DUSP2,IL6R,JAK1
IL-17A Signaling in Gastric Cells	4.16E+00	NaN	CCL20,MAPK10,NFKB1,TNF
PTEN Regulation	4.15E+00	-1.89	ATP,EGR1,HDAC3,PSMB10,PSMB8,PSMC4,RPTOR,TP53
Role of MAPK Signaling in Promoting the Pathogenesis of Influenza	4.15E+00	-1.134	BCL2,MAP3K5,MAPK10,NFKB1,NFKBIA,NRAS,PRKCA
Signaling by NOTCH1	4.14E+00	-0.816	ATP,HDAC3,HES1,JAG1,KAT2B,MYC
Renal Cell Carcinoma Signaling	4.14E+00	-2	NRAS,PIK3CD,PIK3CG,PIK3R1,SLC2A1,TGFB1
OX40 Signaling Pathway	4.13E+00	-2.236	B2M,BCL2,CD3G,HLA-DMA,HLA-DMB,HLA-DOB,HLA-DQA1,HLA-DQB1,HLA-DRB5,HLA-E,HLA-G,MAPK10,NFKB1,NFKBIA
Ephrin Receptor Signaling	4.12E+00	-1.633	AXIN1,FYN,GNG4,ITGA2,ITGA4,ITGAL,NRAS,PIK3CG,VEGFB
TNFR1 Signaling	4.12E+00	-0.447	CASP8,CASP9,NFKB1,NFKBIA,TNF
DDX58/IFIH1-mediated induction of interferon-alpha/beta	4.11E+00	-1.633	ATP,CASP8,IFIH1,NFKB1,NFKBIA,UBA7
FLT3 Signaling in Hematopoietic Progenitor Cells	4.11E+00	-1.633	EIF4EBP1,NRAS,PIK3CD,PIK3CG,PIK3R1,RPS6KB1
Inhibition of ARE-Mediated mRNA Degradation Pathway	4.06E+00	-0.447	CD70,FASLG,LTB,PSMB10,PSMB8,PSMC4,TNF,TNFSF18
Signaling by NOTCH3	4.04E+00	-0.447	ATP,HES1,IKZF1,JAG1,KAT2B
CREB Signaling in Neurons	4.03E+00	-1.732	ADORA2A,ATP,CCR2,CCR5,CCR9,GNG4,NRAS,PIK3CD,PIK3CG,PIK3R1,POLR2A,PRKCA,TBP,TGFB1,TGFBR1,TGFBR2
Allograft Rejection Signaling	4.02E+00	NaN	B2M,CD3G,CD80,CD86,FASLG,HLA-DMA,HLA-DMB,HLA-DOB,HLA-DQA1,HLA-DQB1,HLA-DRB5,HLA-E,HLA-G,TNF
Integrin cell surface interactions	4.00E+00	-2.449	CD47,ICAM2,ITGA2,ITGA4,ITGAL,PECAM1
Estrogen-Dependent Breast Cancer Signaling	4.00E+00	-2.449	ESR1,NFKB1,NRAS,PIK3CD,PIK3CG,PIK3R1
Role of Cytokines in Mediating Communication between Immune Cells	4.00E+00	NaN	IL15,IL18,IL1RN,TGFB1,TNF
Role of MAPK Signaling in the Pathogenesis of Influenza	3.97E+00	NaN	BCL2,MAP3K5,MAPK10,NRAS,PRKCA,TNF
CSDE1 Signaling Pathway	3.92E+00	1.342	CCND3,CTNNB1,MYC,PUM1,TGFB1
Adrenergic Receptor Signaling Pathway (Enhanced)	3.90E+00	0.707	ATP,CD70,FASLG,IL15,IL18,LTB,PRKCA,TGFB1,TNF
mTOR Signaling	3.89E+00	-2.121	EIF4EBP1,NRAS,PIK3CD,PIK3CG,PIK3R1,PRKCA,RPS6KB1,RPTOR,VEGFB
VEGF Family Ligand-Receptor Interactions	3.89E+00	-1.633	NRAS,PIK3CD,PIK3CG,PIK3R1,PRKCA,VEGFB
Interleukin-1 processing	3.88E+00	NaN	CASP1,IL18,NFKB1
Regulation of Cellular Mechanics by Calpain Protease	3.86E+00	NaN	CCND2,CCND3,ITGA2,ITGA4,ITGAL,NRAS
Signaling by NTRK2 (TRKB)	3.86E+00	-1	ATP,FYN,NRAS,PIK3R1
B Cell Receptor Signaling	3.84E+00	-2.496	CD19,CD79B,EGR1,FCGR2B,MAP3K12,MAP3K5,MAP3K8,NFKB1,NFKBIA,NRAS,PIK3CD,PIK3CG,PIK3R1,PTPRC,RPS6KB1,TCF3
Intrinsic Pathway for Apoptosis	3.81E+00	-2.236	ATP,BCL2,CASP8,CASP9,TP53
Oxidative Stress Induced Senescence	3.81E+00	-2.449	ATP,E2F3,MAP3K5,MAPK10,MDM2,TP53

Pyroptosis	3.80E+00	-2	CASP1,IL18,IRF2,TP53
TNFR2 Signaling	3.80E+00	NaN	NFKB1,NFKBIA,TNF,TRAF1
Extra-nuclear estrogen signaling	3.79E+00	-2.449	ATP,BCL2,ESR1,GNG4,NRAS,PIK3R1
FAT10 Signaling Pathway	3.78E+00	NaN	ATP,PSMB10,PSMB8,PSMC4,TNF
Glutaminergic Receptor Signaling Pathway (Enhanced)	3.74E+00	-0.905	GLS,GLUD1,GOT2,NFKB1,PIK3CD,PIK3CG,PIK3R1,PRKCA,RPS6KB1,RPTOR,TGFB1
MAPK6/MAPK4 signaling	3.73E+00	NaN	ATP,CCND3,MYC,PSMB10,PSMB8,PSMC4
Regulation of TP53 Activity through Phosphorylation	3.73E+00	-1.633	ATP,EXO1,MDM2,MRE11,TBP,TP53
Thrombin Signaling	3.73E+00	-2.121	GNG4,NFKB1,NRAS,PIK3CD,PIK3CG,PIK3R1,PRKCA,RPS6KB1,TBP
Signaling by ERBB2	3.71E+00	-0.447	ATP,FYN,NRAS,PIK3R1,PRKCA
MTOR signalling	3.70E+00	-2	ATP,EIF4EBP1,RPS6KB1,RPTOR
Toll Like Receptor 3 (TLR3) Cascade	3.70E+00	-2	ATP,CASP8,RIPK3,TICAM1
CCR3 Signaling in Eosinophils	3.68E+00	-1.633	CCL11,GNG4,NRAS,PIK3CD,PIK3CG,PIK3R1,PRKCA
Reelin Signaling in Neurons	3.68E+00	-1.89	BLK,FYN,LCK,MAPK10,PIK3CD,PIK3CG,PIK3R1
IL-1 Signaling	3.66E+00	-1.342	ATP,GNG4,MAPK10,MYD88,NFKB1,NFKBIA
Response to elevated platelet cytosolic Ca^{2+}	3.61E+00	-0.378	ALDOA,ATP,CD36,PECAM1,PRKCA,TGFB1,VEGFB
Oncogene Induced Senescence	3.60E+00	NaN	ATP,E2F3,MDM2,TP53
Apelin Cardiomyocyte Signaling Pathway	3.55E+00	-1.633	MAPK10,PIK3CD,PIK3CG,PIK3R1,PRKCA,TGFB1
S Phase	3.52E+00	-1.633	ATP,MYC,PSMB10,PSMB8,PSMC4,RBL2
TNFR2 non-canonical NF-kB pathway	3.52E+00	-1.342	LTB,PSMB10,PSMB8,PSMC4,TNF
Polyamine Regulation in Colon Cancer	3.52E+00	-0.447	CTNNB1,MYC,OAZ1,RPTOR,TP53
PPAR Signaling	3.48E+00	0.816	IL18,IL1RN,NFKB1,NFKBIA,NRAS,TNF
ERBB4 Signaling	3.46E+00	-1.342	NRAS,PIK3CD,PIK3CG,PIK3R1,PRKCA
Glutamate and glutamine metabolism	3.46E+00	0	ATP,GLS,GLUD1,GOT2
Hedgehog ligand biogenesis	3.43E+00	-2.236	ATP,IHH,PSMB10,PSMB8,PSMC4
GM-CSF Signaling	3.43E+00	-2.236	CSF2RB,NRAS,PIK3CD,PIK3CG,PIK3R1
TP53 Regulates Transcription of DNA Repair Genes	3.40E+00	-1.342	ATP,MLH1,PMS2,POLR2A,TP53
MAP kinase activation	3.38E+00	-2	ATP,MAP3K8,MAPK10,NFKB1
RIPK1-mediated regulated necrosis	3.38E+00	-2	ATP,CASP8,FASLG,RIPK3
ISG15 antiviral mechanism	3.35E+00	-0.447	ATP,EIF2AK2,FLNB,JAK1,UBA7
April Mediated Signaling	3.34E+00	-2	MAPK10,NFKB1,NFKBIA,TRAF1
Signal regulatory protein family interactions	3.33E+00	NaN	ATP,CD47,SIRPA
Growth Hormone Signaling	3.32E+00	-1.342	PIK3CD,PIK3CG,PIK3R1,PRKCA,RPS6KB1
Regulation of TP53 Expression and Degradation	3.30E+00	-2	ATP,MDM2,PRDM1,TP53
RET signaling	3.30E+00	-1	ATP,PIK3CD,PIK3R1,PRKCA
B Cell Activating Factor Signaling	3.30E+00	NaN	MAPK10,NFKB1,NFKBIA,TRAF1
CDC42 Signaling	3.29E+00	-2	B2M,CD3G,HLA-DMA,HLA-DMB,HLA-DOB,HLA-DQA1,HLA-DQB1,HLA-DRB5,HLA-E,HLA-G,ITGA2,ITGA4,ITGAL,MAPK10
Synaptogenesis Signaling Pathway	3.29E+00	-1	ATP,CTNNB1,EIF4EBP1,FYN,LCK,NRAS,PIK3CD,PIK3CG,PIK3R1,RPS6KB1
Integrin Signaling	3.27E+00	-2.121	FYN,ITGA2,ITGA4,ITGAL,NRAS,PIK3CD,PIK3CG,PIK3R1
Axonal Guidance Signaling	3.25E+00	NaN	FYN,GNG4,ITGA2,ITGA4,ITGAL,NRAS,PIK3CD,PIK3CG,PIK3R1,PRKCA,VEGFB,WNT10A,WNT2B
Role of WNT/GSK-3β Signaling in the Pathogenesis of Influenza	3.24E+00	NaN	AXIN1,CTNNB1,TCF3,WNT10A,WNT2B

Relaxin Signaling	3.23E+00	-2.449	ATP,GNG4,NFKB1,NFKBIA,PIK3CD,PIK3CG,PIK3R1
Regulation of RUNX2 expression and activity	3.22E+00	-2.236	ATP,ESR1,PSMB10,PSMB8,PSMC4
GDNF Family Ligand-Receptor Interactions	3.22E+00	-2.236	MAPK10,NRAS,PIK3CD,PIK3CG,PIK3R1
Antioxidant Action of Vitamin C	3.21E+00	0.447	CSF2RB,MAPK10,NFKB1,NFKBIA,SLC2A1,TNF
Sperm Motility	3.21E+00	NaN	ATP,BLK,FYN,GNG4,JAK1,JAK3,LCK,PRKCA,TWF1
Role of RIG1-like Receptors in Antiviral Innate Immunity	3.18E+00	NaN	CASP8,IFIH1,NFKB1,NFKBIA
Antiproliferative Role of Somatostatin Receptor 2	3.17E+00	-2	GNG4,NRAS,PIK3CD,PIK3CG,PIK3R1
IL-15 Signaling	3.17E+00	-3.051	IL15,IL2RG,JAK1,JAK3,LCK,MYC,NFKB1,NRAS,PIK3CD,PIK3CG,PIK3R1,RPS6KB1,RPTOR
Autoimmune Thyroid Disease Signaling	3.16E+00	NaN	CD3G,CD80,CD86,FASLG,HLA-DMA,HLA-DMB,HLA-DOB,HLA-DQA1,HLA-DQB1,HLA-DRB5,HLA-E,HLA-G
Protein Ubiquitination Pathway	3.16E+00	NaN	ATP,B2M,HLA-E,HLA-G,MDM2,PSMB10,PSMB8,PSMC4,TAP2
Role of NFAT in Regulation of the Immune Response	3.15E+00	-3.771	CD3G,CD79B,CD80,CD86,FCGR2B,FYN,GNG4,HLA-DMA,HLA-DMB,HLA-DOB,HLA-DQA1,HLA-DQB1,HLA-DRB5,LCK,NFKB1,NFKBIA,NRAS,PIK3CD,PIK3CG,PIK3R1
FasL/ CD95L signaling	3.14E+00	NaN	CASP8,FASLG
Cholecystokinin/Gastrin-mediated Signaling	3.14E+00	-0.816	IL18,IL1RN,MAPK10,NRAS,PRKCA,TNF
Sphingosine-1-phosphate Signaling	3.14E+00	0	CASP1,CASP8,CASP9,PIK3CD,PIK3CG,PIK3R1
Signaling by Type 1 Insulin-like Growth Factor 1 Receptor (IGF1R)	3.13E+00	NaN	ATP,NRAS,PIK3R1
Neurotrophin/TRK Signaling	3.12E+00	-2.236	MAP3K5,NRAS,PIK3CD,PIK3CG,PIK3R1
Ephrin A Signaling	3.08E+00	NaN	FYN,PIK3CD,PIK3CG,PIK3R1
Chemokine Signaling	3.07E+00	-1.342	CCL11,CCR5,NRAS,PIK3CG,PRKCA
Gαq Signaling	3.04E+00	-1.342	GNG4,NFKB1,NFKBIA,PIK3CD,PIK3CG,PIK3R1,PRKCA
Oxytocin Signaling Pathway	3.02E+00	-2.333	CD36,GNG4,MAPK10,NFKB1,NRAS,PIK3CD,PIK3CG,PIK3R1,PRKCA
UVC-Induced MAPK Signaling	3.01E+00	-1	MAPK10,NRAS,PRKCA,TP53
Regulation of TP53 Activity through Methylation	3.01E+00	NaN	ATP,MDM2,TP53
Gastrin-CREB signalling pathway via PKC and MAPK	3.01E+00	NaN	ATP,NRAS,PRKCA
Pre-NOTCH Expression and Processing	3.01E+00	-1.342	ATP,E2F3,KAT2B,MFNG,TP53
Hedgehog 'on' state	3.01E+00	-2.236	ATP,IHH,PSMB10,PSMB8,PSMC4

*Z-score: positive score = activated pathway; negative score = inhibited pathway; NA = no activity pattern available.

References

1. Hallek M, Cheson BD, Catovsky D, et al. iwCLL guidelines for diagnosis, indications for treatment, response assessment, and supportive management of CLL. *Blood.* 2018;131(25):2745-2760.
2. Visentin A, Bonaldi L, Rigolin GM, et al. The combination of complex karyotype subtypes and IGHV mutational status identifies new prognostic and predictive groups in chronic lymphocytic leukaemia. *Br J Cancer.* 2019;121(2):150-156.

SUPPLEMENTAL METHODS

In vivo fludarabine treatment

Fludarabine phosphate (FLU) was purchased from Selleck Chemicals (Houston, TX). FLU was dissolved in 0.9% sodium chloride (Hospira; Lake Forest, IL) and administered intraperitoneally[4] at 34 mg/kg, as outlined in Supplemental Figure 18A.

Visualization of public ChIP-seq datasets

Chromatin immunoprecipitation sequencing analysis was retrieved from the GEO repository (accession ID: GSE183883)[8]. BigWig files were imported into Integrative Genomics Viewer v2.8.0[9] (IGV, Broad Institute) and aligned to reference genome hg19 to visualize specific loci.

Flow cytometry

A- Sample preparation

Human and murine cells (~1-2 e^6) were suspended in PBS/5% hi-FBS (100 uL) and incubated with fluorochrome-labelled antibodies at 4°C for 20 min. To monitor leukemic disease burden in mice, ~25 μL blood obtained from the submandibular vein. Whole blood was incubated with fluorochrome-labelled antibodies at 4°C for 20 min then lysed using RBC Lysis Buffer (BioLegend; San Diego, CA) per manufacturer protocol prior to flow cytometry analysis.

B- Antibody list

Mouse-specific antibodies					Experiment			
**Target***	**Fluorochrome**	**Clone**	**Host and Isotype**	**Supplier**	**T-cell prolif-eration**	**T-cell effector function**	**Immune IRs/TFs**	**Disease burden**
B220	BUV395	RA3-6B2	Rat IgG2a, κ	BD Biosc.			X	
B220	PerCP	RA3-6B2	Rat IgG2a, κ	BioLegend				X
CD107a	APC	1D4B	Rat IgG2a, κ	BioLegend		X		
CD127	FITC	A7R34	Rat IgG2a, κ	BioLegend				
CD160	PE	7H1	Rat IgG2a, κ	BioLegend			X	
CD19	Brilliant Violet 605	6D5	Rat IgG2a, κ	BioLegend			X	
CD19	PE	1D3	Rat IgG2a, κ	BD Biosc.				X
CD244.2	APC	m2B4 (B6)458.1	Mouse IgG1, κ	BioLegend			X	
CD3	Brilliant Violet 650	17A2	Rat IgG2b, κ	BioLegend		X		
CD3ε	BUV737	145-2C11	Armenian Hamster IgG1, κ	BD Biosc.			X	
CD3ε	PE/Cyanine7	145-2C11	Armenian Hamster IgG	BioLegend				X

CD4	PerCP/Cyanine5.5	RM4-5	Rat IgG2a, κ	BioLegend	X			
CD4	Brilliant Violet 785	RM4-5	Rat IgG2a, κ	BioLegend			X	
CD44	PE	IM7	Rat IgG2b, κ	BioLegend	X			
CD44	FITC	IM7	Rat IgG2b, κ	BioLegend		X	X	
CD45	Alexa Fluor 700	30-F11	Rat IgG2b, κ	BioLegend		X	X	
CD45	APC	30-F11	Rat IgG2b, κ	BioLegend				X
CD5	Brilliant Violet 711	53-7.3	Rat IgG2a, κ	BioLegend			X	
CD5	FITC	53-7.3	Rat IgG2a, κ	BD Biosc.				X
CD62L	APC	MEL-14	Rat IgG2a, κ	BioLegend	X			
CD62L	PE/Cyanine5	MEL-14	Rat IgG2a, κ	BioLegend		X	X	
CD8a	FITC	53-6.7	Rat IgG2a, κ	BioLegend	X			
CD8a	Brilliant Violet 510	53-6.7	Rat IgG2a, κ	BioLegend		X	X	
IFN-γ	PE/Dazzle 594	XMG1.2	Rat IgG1, κ	BioLegend		X		
IL-2	PE/Cyanine7	JES6-5H4	Rat IgG2b, κ	BioLegend		X		
IL-4	PE	11B11	Rat IgG1, κ	BioLegend		X		
KLRG1	PerCP/ Cyanine 5.5	2F1/ KLRG1	Syrian hamster IgG	BioLegend			X	
Ki67	PE	16A8	Rat IgG2a, κ	BioLegend		X		
LAG3	PE/Cyanine7	C9B7W	Rat IgG1, κ	BioLegend			X	
PD-1	PE/Dazzle 594	RMP1-30	Rat IgG2b, κ	BioLegend			X	
PD-1	APC	29F.1A12	Rat IgG2a, κ	BioLegend			X	
PD-1	PE/Cyanine7	29F.1A12	Rat IgG2a, κ	BioLegend			X	
PD-L1	Brilliant Violet 421	10F.9G2	Rat IgG2b, κ	BioLegend			X	
RUNX3	Alex Fluor 488	R3-5G4	Mouse IgG1, κ	BD Biosc			X	
TBET	PE	4B10	Mouse IgG1, κ	BioLegend			X	
TCF1	Brilliant Violet 421	S33-966	Mouse IgG1, κ	BD Biosc			X	
TIM3	Brilliant Violet 711	B8.2C12	Rat IgG1, κ	BioLegend			X	
TNF-α	Brilliant Violet 421	MP6-XT22	Rat IgG1, κ	BioLegend		X		
TOX	eFluor 660	TXRX10	Rat IgG2a, κ	Invitrogen			X	
VISTA	Brilliant Violet 650	MIH64	Rat IgG2a, κ	BD Biosc.			X	
Zombie NIR™ Fixable Viability Dye				BioLegend	X	X	X	
**Human-specific antibodies**					**Experiment**			
**Target***	**Fluorochrome**	**Clone**	**Host and Isotype**	**Supplier**	**T-cell prolife-ration**	**T-cell effector function**	**Immune receptors**	**T-cell TFs**
BATF	PE	9B5A13	Mouse IgG2b, κ	BioLegend				X
BTLA	FITC	MIH26	Mouse IgG2a, κ	BioLegend			X	
CCR7	PE/Dazzle 594	G043H7	Mouse IgG2a, κ	BioLegend		X		
CD101	APC	BB27	Mouse IgG1, κ	BioLegend			X	
CD107a	APC	H4A3	Mouse IgG1, κ	BioLegend		X		
CD127	Brilliant Violet 650	A019D5	Mouse IgG1, κ	BioLegend			X	X
CD160	PerCP/Cyanine5.5	BY55	Mouse IgM, κ	BioLegend			X	
CD19	APC	HIB19	Mouse IgG1, κ	BD Biosc.	X		X	
CD19	UV737	SJ25C1	Mouse IgG1, κ	BD Biosc.			X	X

CD19	Brilliant Violet 650	HIB19	Mouse IgG1, κ	BioLegend			X	X
CD244	Brilliant Violet 421	C1.7	Mouse IgG1, κ	BioLegend			X	
CD3	FITC	UCHT-1	Mouse IgG1, κ	Leinco		X		
CD4	Brilliant Violet 450	RPA-T4	Mouse IgG1, κ	BioLegend	X			
CD4	Brilliant Violet 785	RPA-T4	Mouse IgG1, κ	BioLegend			X	
CD4	Alexa Fluor 700	SK3	Mouse IgG1, κ	BioLegend				X
CD44	Alexa Fluor 700	C44Mab-5	Mouse IgG1, κ	BioLegend			X	X
CD45	Alexa Fluor 700	HI30	Mouse IgG1, κ	BioLegend	X			
CD45Ra	Brilliant Violet 421	HI100	Mouse IgG2b, κ	BioLegend		X		
CD62L	PerCP/Cy5.5	DREG-56	Mouse IgG1, κ	BioLegend			X	X
CD8	Brilliant Violet 510	SK1	Mouse IgG1, κ	BioLegend	X		X	X
CD8	PerCP	RPA-T8	Mouse IgG1, κ	BioLegend		X		
CD80	PE/Cyanine7	2D10	Mouse IgG1, κ	BioLegend			X	
CD86	PerCP	BU63	Mouse IgG1, κ	Invitrogen			X	
CTLA4	Brilliant Violet 421	BNI3	Mouse IgG2a, κ	BioLegend			X	
CXCR5	PE/Cyanine7	J252D4	Mouse IgG1, κ	BioLegend				
EOMES	BUV395	X4-83	Mouse IgG1, κ	BD Biosc.				X
GZMB	FITC	QA18A28	Rat IgG1, κ	BioLegend		X		
HLA-DR	Alexa Fluor 700	L243	Mouse IgG2a, κ	BioLegend			X	
ICOS	Brilliant Violet 605	C398.4A	Armenian Hamster IgG	BioLegend				
IFN-γ	Brilliant Violet 785	4S.B3	Mouse IgG1, κ	BioLegend		X		
IFN-γ	PE/Cyanine7	4S.B3	Mouse IgG1, κ	BioLegend		X		
IL-2	Alexa Fluor 700	MQ1-17H12	Rat IgG2a, κ	BioLegend		X		
KLRG1	Brilliant Violet 421	SA231A2	Mouse IgG2a, κ	BioLegend			X	X
LAG3	PE/Cyanine7	11C3C65	Mouse IgG1, κ	BioLegend			X	
Ly108	PE/Cyanine7	W19035D	Rat IgG2a, κ	BioLegend			X	X
PD-1	PE	EH12.2H7	Mouse IgG1, κ	BioLegend			X	
PD-1	Brilliant Violet 605	NAT105	Mouse IgG1, κ	BioLegend			X	X
PD-1	APC	EH12.2H7	Mouse IgG1, κ	BioLegend				X
PD-L1	PE/Dazzle 594	29E.2A3	Mouse IgG2b, κ	BioLegend			X	
RUNX3	Alex Fluor 488	R3-5G4	Mouse IgG1, κ	BD Biosc.				X
T-BET	Brilliant Violet 421	4B10	Mouse IgG1, κ	BioLegend				X
TCF-1	Alexa Fluor 647	7F11A10	Mouse IgG1, κ	BioLegend				X
TCF-1	PE	7F11A10	Mouse IgG1, κ	BioLegend				X
TIM3	FITC	F38-2E2	Mouse IgG1, κ	BioLegend			X	
TIM3	Brilliant Violet 711	F38-2E2	Mouse IgG1, κ	BioLegend			X	X
TNF-α	Brilliant Violet 510	MAb11	Mouse IgG1, κ	BioLegend		X		
TNF-α	PE Dazzle	MAb11	Mouse IgG1, κ	BioLegend		X		
TOX	eFluor 660	TXRX10	Rat IgG2a, κ	Invitrogen				X
LIVE/DEAD™ Fixable Near-IR Dead Cell Stain				Invitrogen	X			
Zombie NIR™ Fixable Viability Dye				BioLegend		X	X	X

* When appropriate, cells were fixed with Fixation Buffer, Cyto-Fast™ Fix/Perm Buffer Set for intracellular staining, or True-Nuclear™ Transcription Factor Buffer Set for intranuclear staining per manufacturer protocols (BioLegend). IR = inhibitory receptor, TF = transcription factor.

C- Flow cytometry gating strategies

Singlet lymphocytes were gated by forward and side scatter, and live cells were gated by Live/Dead Near IR (Invitrogen) negative staining. We ensured correct compensation, proper acquisition setup and set positive gates based on appropriate fluorescence minus one (FMO) controls. Results are expressed as median fluorescent intensity (MFI) or the proportion of cells expressing antigens of interest (percent gated positive).

Supplemental Methods Figure 1

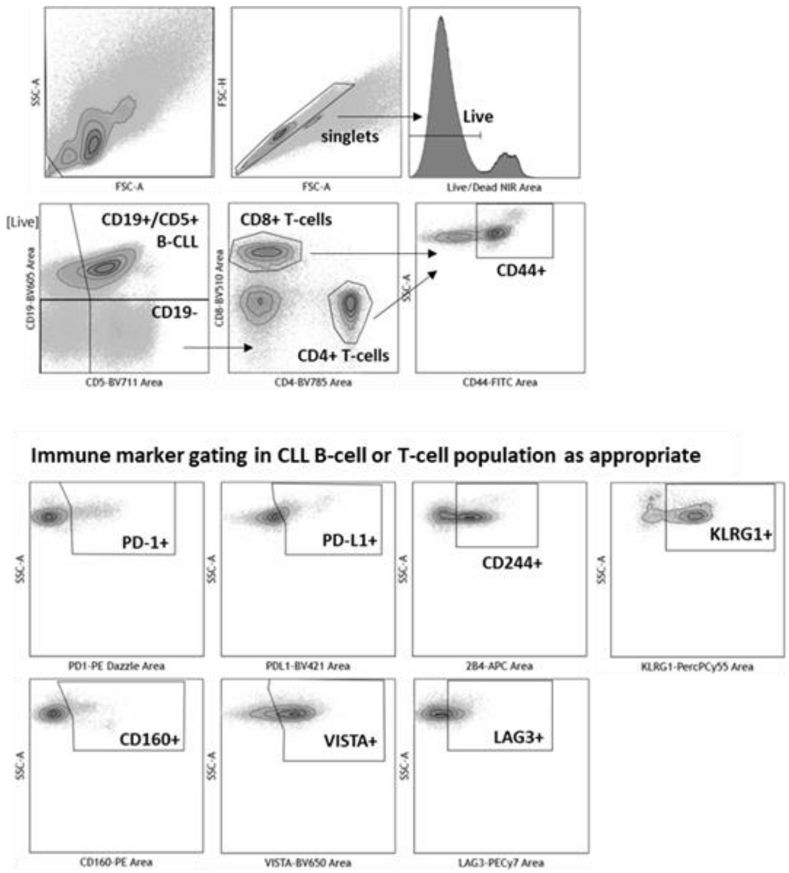

Supplemental Methods Figure 1. Flow cytometry gating strategy used to evaluate murine splenic immune marker expression on the indicated cell populations. Positive gates for PD-1, PD-L1, CD244, CD160, VISTA, LAG3, and KLRG1 were set on FMO controls.

Supplemental Methods Figure 2

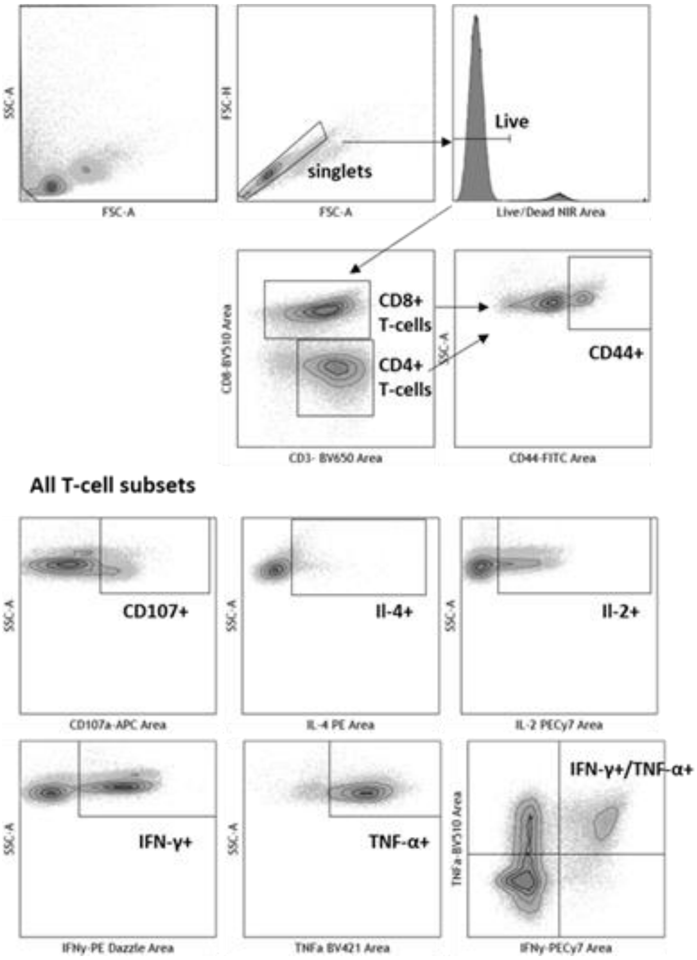

Supplemental Methods Figure 2. Flow cytometry gating strategy used to evaluate murine splenic T-cell CD107a membrane localization and intracellular cytokine production upon PMA/ionomycin stimulation. Positive gates for CD107a, IL-4, IL-2, IFN-γ, and TNF-α were set on FMO controls.

Supplemental Methods Figure 3

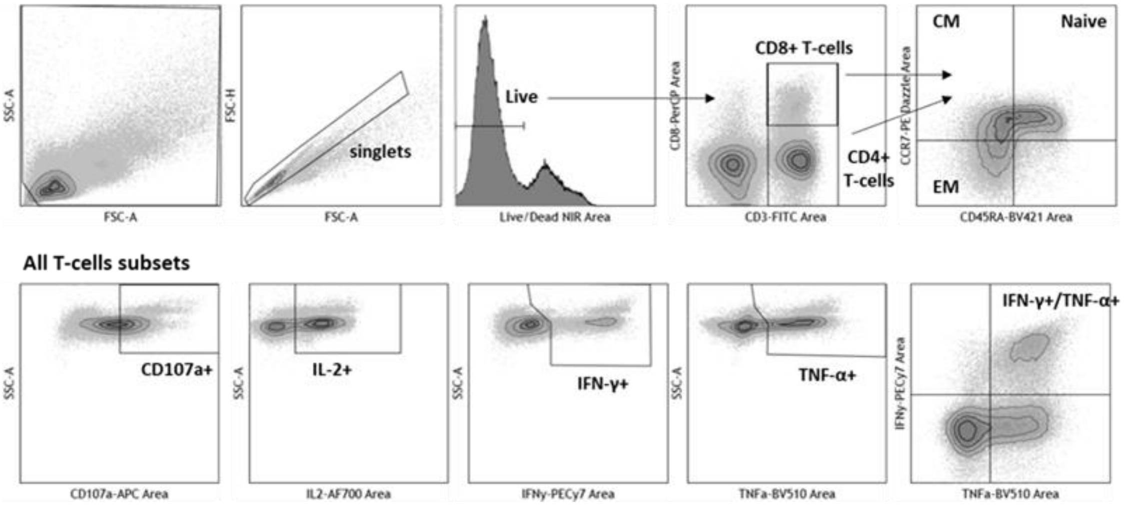

Supplemental Methods Figure 3. Flow cytometry gating strategy used to evaluate human T-cell CD107a membrane localization and intracellular cytokine production upon PMA/ionomycin stimulation. Positive gates for CD107a, IL-2, IFN-γ, and TNF-α were set on FMO controls.

Supplemental Methods Figure 4

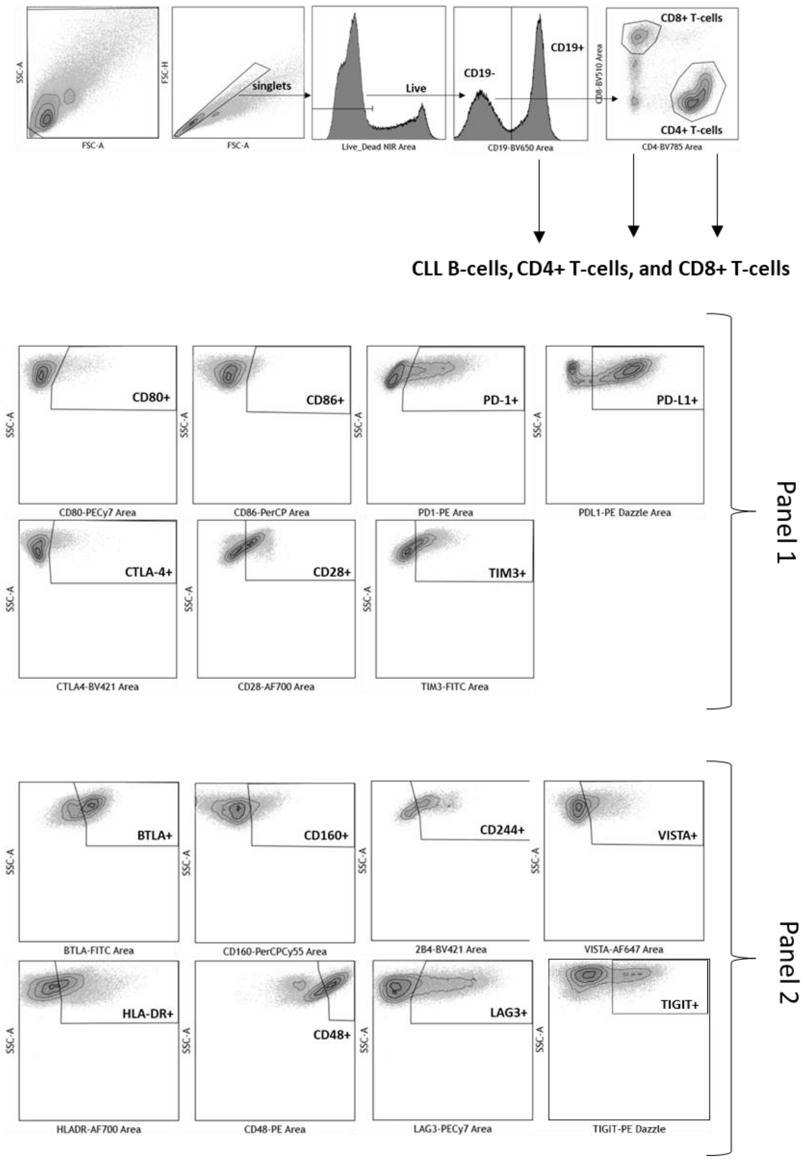

Supplemental Methods Figure 4. Flow cytometry gating strategy used to evaluate human immune receptor expression on the indicated cell populations. Positive gates for all immune inhibitory and stimulatory markers were set on FMO controls.

Supplemental Methods Figure 5

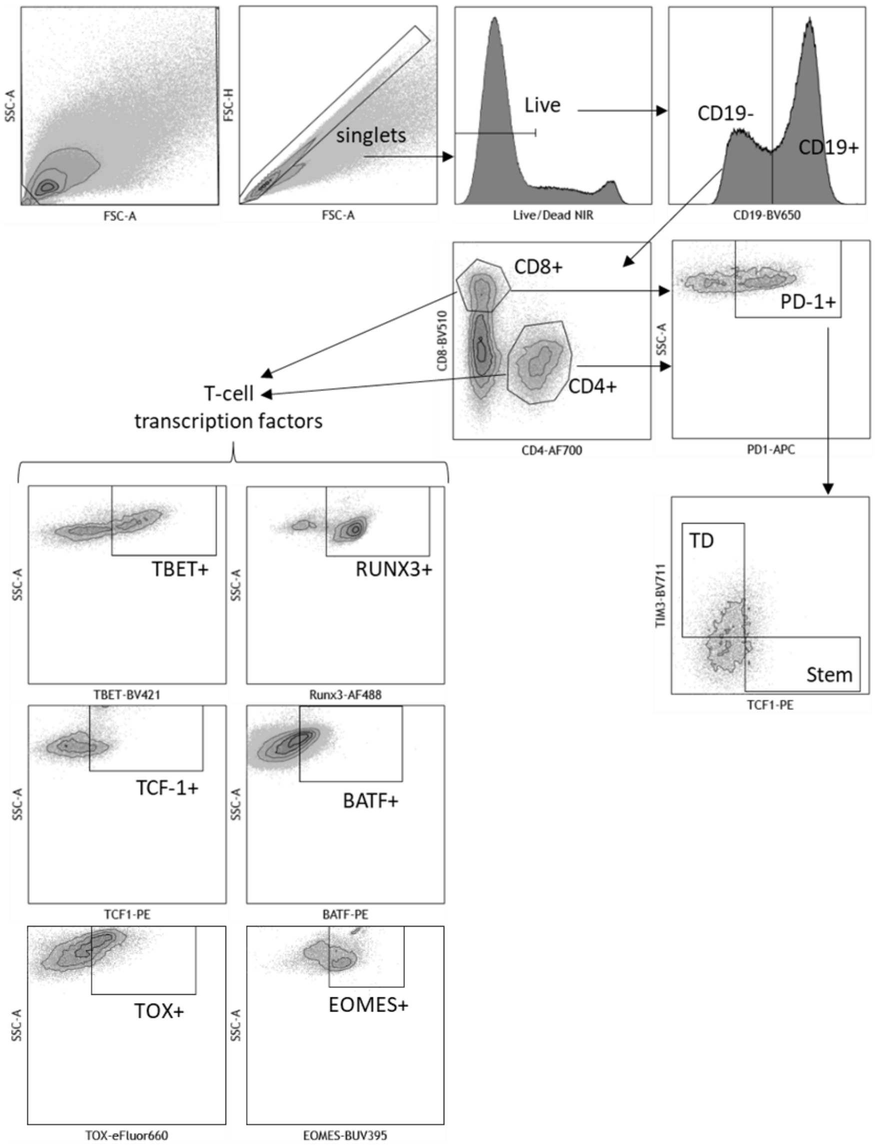

Supplemental Methods Figure 5. Flow cytometry gating strategy used to evaluate T-cell transcription factor expression. Example gating is shown on a vehicle-treated healthy donor T-cell/CLL B-cell co-culture. Similar gating was applied for CLL patient T-cells and murine T-cells. Positive gates for all markers were set on FMO controls.

Supplemental Methods Figure 6

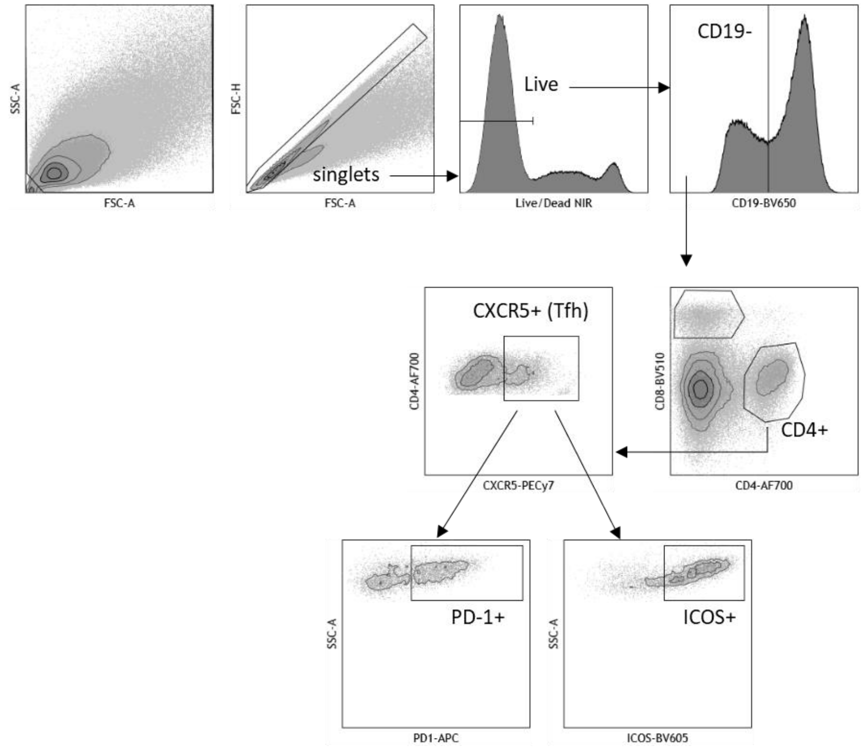

Supplemental Methods Figure 6. Flow cytometry gating strategy used to evaluate human T follicular helper (Tfh) cells and their expression of PD-1 and ICOS. Positive gates for all markers were set on FMO controls.

Supplemental Methods Figure 7

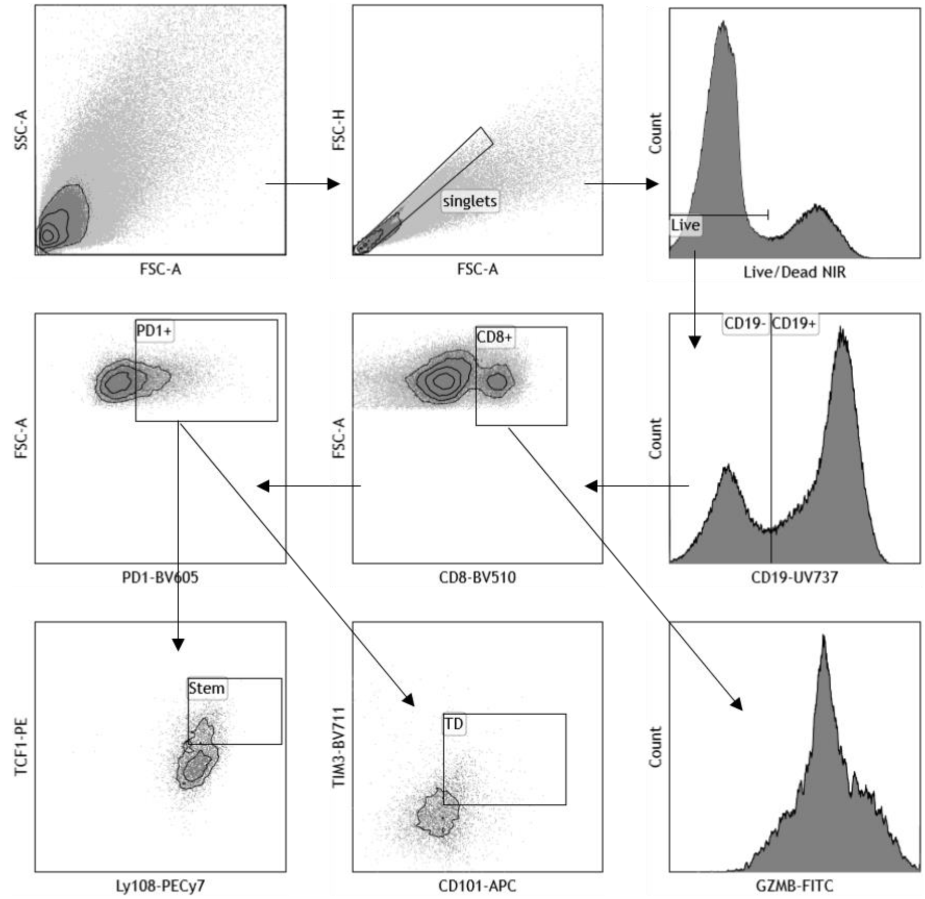

Supplemental Methods Figure 7. Flow cytometry gating strategy used to evaluate T-cell phenotype in time course co-cultures. $CD8^+$ T-cells were assayed for PD1, TCF1, Ly108, TIM3, and CD101 expression to confirm differences in stem-like vs. terminally differentiated exhausted T-cells. Granzyme B (GZMB) expression median fluorescent intensity was also evaluated on $CD8^+$ T-cells. Positive gates for all markers were set on FMO controls.

Supplemental Methods Figure 8

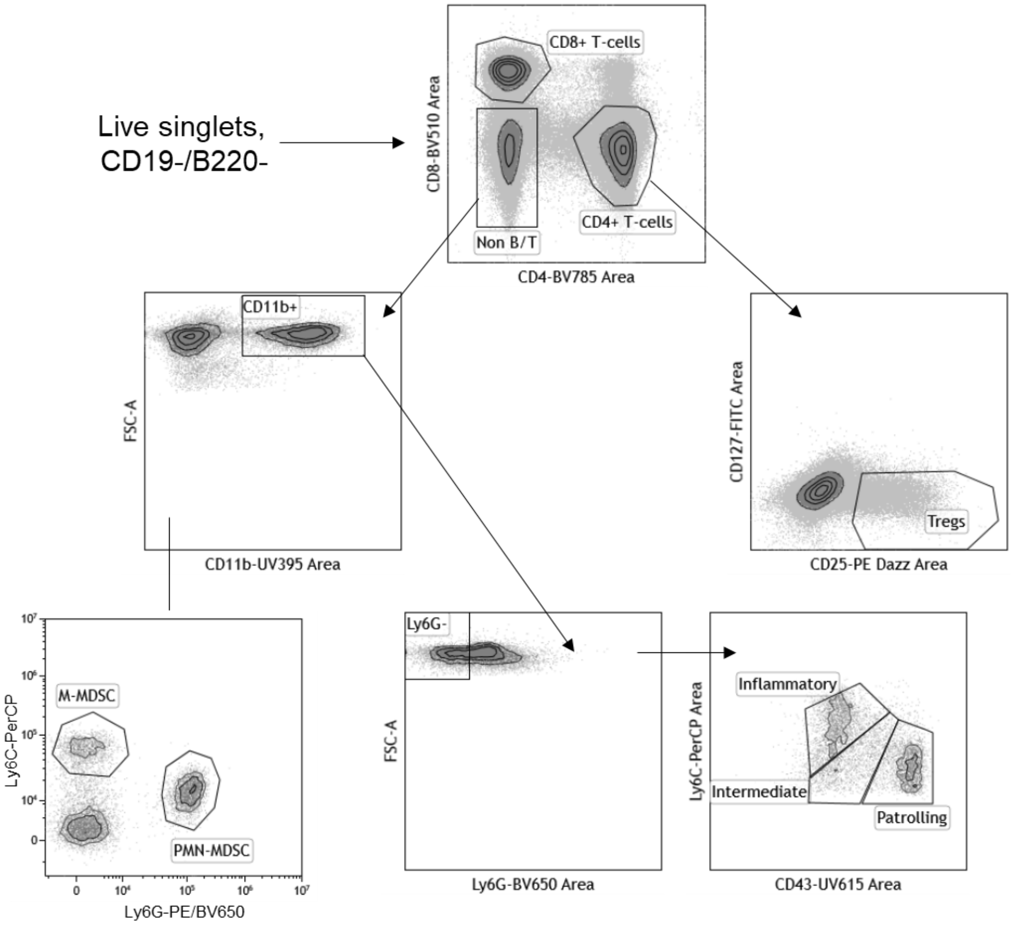

Supplemental Methods Figure 8. Flow cytometry gating strategy to identify additional immune populations in murine spleens. Regulatory T-cells (Tregs), monocytic myeloid-derived suppressor cells (M-MDSC), polymorphonuclear (PMN) MDSCs, and subpopulations of monocytes (inflammatory, intermediate, patrolling) were identified as detailed in Supplemental Figure 4.

References

1. Hallek M, Cheson BD, Catovsky D, et al. iwCLL guidelines for diagnosis, indications for treatment, response assessment, and supportive management of CLL. *Blood.* 2018;131(25):2745-2760.
2. Visentin A, Bonaldi L, Rigolin GM, et al. The combination of complex karyotype subtypes and IGHV mutational status identifies new prognostic and predictive groups in chronic lymphocytic leukaemia. *Br J Cancer.* 2019;121(2):150-156.
3. Ozer HG, El-Gamal D, Powell B, et al. BRD4 Profiling Identifies Critical Chronic Lymphocytic Leukemia Oncogenic Circuits and Reveals Sensitivity to PLX51107, a Novel Structurally Distinct BET Inhibitor. *Cancer Discov.* 2018;8(4):458-477.
4. Johnson AJ, Lucas DM, Muthusamy N, et al. Characterization of the TCL-1 transgenic mouse as a preclinical drug development tool for human chronic lymphocytic leukemia. *Blood.* 2006;108(4):1334-1338.
5. Kramer A, Green J, Pollard J, Jr., Tugendreich S. Causal analysis approaches in Ingenuity Pathway Analysis. *Bioinformatics.* 2014;30(4):523-530.
6. Liberzon A, Subramanian A, Pinchback R, Thorvaldsdottir H, Tamayo P, Mesirov JP. Molecular signatures database (MSigDB) 3.0. *Bioinformatics.* 2011;27(12):1739-1740.
7. Saadey AA, Yousif A, Osborne N, et al. Rebalancing TGFbeta1/BMP signals in exhausted T cells unlocks responsiveness to immune checkpoint blockade therapy. *Nat Immunol.* 2023;24(2):280-294.
8. Snyder KJ, Choe HK, Gao Y, et al. Inhibition of Bromodomain and Extra Terminal (BET) Domain Activity Modulates the IL-23R/IL-17 Axis and Suppresses Acute Graft-Versus-Host Disease. *Front Oncol.* 2021;11:760789.
9. Robinson JT, Thorvaldsdottir H, Winckler W, et al. Integrative genomics viewer. *Nat Biotechnol.* 2011;29(1):24-26.
10. Van Gassen S, Callebaut B, Van Helden MJ, et al. FlowSOM: Using self-organizing maps for visualization and interpretation of cytometry data. *Cytometry A.* 2015;87(7):636-645.
